# Future changes to the upper ocean Western Boundary Currents across two generations of climate models

**DOI:** 10.1038/s41598-021-88934-w

**Published:** 2021-05-05

**Authors:** Alex Sen Gupta, Annette Stellema, Gabriel M. Pontes, Andréa S. Taschetto, Adriana Vergés, Vincent Rossi

**Affiliations:** 1 Climate Change Research Centre, University of New South Wales, Sydney, Australia; 2 Australian Research Council Centre of Excellence for Climate Extremes, University of New South Wales, Sydney, Australia; 3 Centre for Marine Science and Innovation, University of New South Wales, Sydney, Australia; 4 Institute of Oceanography, University of São Paulo, São Paulo, Brazil; 5 Centre for Marine Science & Innovation and Evolution & Ecology Research Centre, School of Biological, Earth and Environmental Sciences, UNSW Australia, Sydney, NSW Australia; 6 Mediterranean Institute of Oceanography (UM 110, UMR 7294), CNRS, Aix Marseille Univ., Univ. Toulon, IRD, 13288 Marseille, France

**Keywords:** Climate-change ecology, Marine biology, Physical oceanography, Climate and Earth system modelling, Climate-change impacts

## Abstract

Western Boundary Currents (WBCs) are important for the oceanic transport of heat, dissolved gases and nutrients. They can affect regional climate and strongly influence the dispersion and distribution of marine species. Using state-of-the-art climate models from the latest and previous *Climate Model Intercomparison Projects*, we evaluate upper ocean circulation and examine future projections, focusing on subtropical and low-latitude WBCs. Despite their coarse resolution, climate models successfully reproduce most large-scale circulation features with ensemble mean transports typically within the range of observational uncertainty, although there is often a large spread across the models and some currents are systematically too strong or weak. Despite considerable differences in model structure, resolution and parameterisations, many currents show highly consistent projected changes across the models. For example, the East Australian Current, Brazil Current and Agulhas Current extensions are projected to intensify, while the Gulf Stream, Indonesian Throughflow and Agulhas Current are projected to weaken. Intermodel differences in most future circulation changes can be explained in part by projected changes in the large-scale surface winds. In moving to the latest model generation, despite structural model advancements, we find little systematic improvement in the simulation of ocean transports nor major differences in the projected changes.

## Introduction

Anthropogenic climate change manifests as increases in surface temperature and sea level, rainfall distribution changes and increasing frequency and intensity of certain extreme events^1^. An often underappreciated aspect of climate change relates to upper ocean circulation. Surface ocean currents play a crucial role in the redistribution of heat^2^, pollutants^3^, plastics^4^, biogeochemical tracers^5^ and species dispersal—particularly during passive early-life stages (e.g. eggs, larvae), influencing the distribution of marine species^6^.

Particularly important for heat transport, air-sea interactions and marine ecosystems are the energetic Western Boundary Currents (WBCs); narrow (~100–200 km), intense (~1 m/s) jets in the upper several hundred meters off the continental shelves in the western basins. Subtropical WBCs form the poleward flanks of the subtropical gyres. Their poleward extensions form high-variability regions where the WBCs often leave the coast. Equatorward flowing WBCs at low latitudes play an important role in tropical circulation, facilitating cross-equatorial and inter-basin connectivity.

The large-scale upper-ocean circulation is primarily driven by surface winds. In the subtropics, north-south surface wind gradients, associated with climatological high-pressure systems, drive an equatorward *Sverdrup* transport in the ocean interior^7^. To conserve potential vorticity this flow is largely compensated by intense, poleward WBCs forming partially closed subtropical gyres. In the Indian and Pacific basins, however, some water from the Agulhas Current (AC) and East Australian Current (EAC) leak from the subtropical gyres, escaping westward south of Africa (AC extension) and Australia (Tasman Leakage), respectively. Low-latitude WBCs (LLWBCs) also play a role in the leakage of water from the Pacific to Indian basins: the Mindanao Current (MC) and, to a lesser extent, the New Guinea Coastal Undercurrent (NGCU), feed the climatically important Indonesian Throughflow (ITF)^8^. The intense WBCs are typically associated with high levels of mesoscale activity that alter the characteristics of the WBCs; for example WBCs tend to be faster, narrower and better located in eddy-permitting/resolving models compared to models that cannot resolve mesoscale activity^9^.

Climate models project consistent future changes to the surface winds^1^ including a poleward expansion of the Hadley Circulation^10^, a poleward intensification of the subtropical westerlies^11^, and a slowdown of the Walker circulation and equatorial trade winds^12^. Such changes would modify the upper ocean circulation. Using multiple lines of evidence, previous work^13^ has identified a centennial scale poleward intensifications of the subtropical WBCs (excluding the Gulf Stream, GS), although natural variability can mask these changes even on multi-decadal timescales^14,15^. These studies link the WBC changes to the poleward intensification of the subtropical westerlies and the expansion of the Hadley Cell in both hemispheres, and the slowdown of the Atlantic meridional overturning circulation in the case of the GS. The intensified heat transport and/or poleward shift of these WBCs have led to hotspots that have warmed 2–3 times faster than the global average^16,17^. This warming has increased the occurrence of marine heatwaves^18^ and affected larval dispersal, causing dramatic modifications to marine ecosystems. For example, the range expansion of some consumers has led to extensive overgrazing of kelp forests, with major cascading impacts on entire ecosystems^19,20^.

The intensification of WBCs has been associated with the ‘tropicalisation’ of nearshore marine communities as warm-affinity species expand their distribution poleward^21^, impacting ecosystem functions like productivity^22^. Shifts in WBCs have already impacted fisheries in meaningful ways. For instance, a northward shift in the GS has been linked to the collapse of the Gulf of Maine cod fishery^23^. Future climate-induced changes in ocean circulation are also projected to alter coastal retention and larval dispersal pathways^24^.

Previous studies using ocean and coupled climate models from previous generations of the Coupled Model Intercomparison Project (CMIP) suggest robust future circulation changes in different basins^17,25–32^. Using the new CMIP6 ensemble, this study examines major WBCs and associated circulation features, including model representation of transport and seasonality in comparison to observations and reanalysis products, projected circulation changes and connections to surface wind changes. We also examine any changes compared to the CMIP5 model ensemble.

## Results

In the following, model transports and projected changes are expressed as ensemble interquartile ranges with individual model details provided in Tables S1-S10, reanalysis transport estimates are provided as the range across the three products examined (Table S11), and observational transports and associated references are provided in Table S12. Projections represent differences between the 1900–2000 *historical* mean and 2050–2100 means from the business-as-usual *SSP5-8.5* (*RCP8.5)* scenarios for CMIP6 (CMIP5).

### Indian Ocean

**Figure 1:**
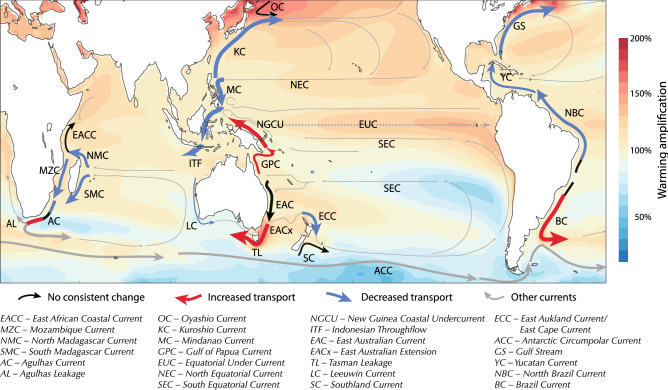
Schematic showing projected changes in WBC transport. Background colours show the multi-model mean projected change in sea surface temperature divided by the global mean change, e.g. 150% implies a warming rate 1.5 × the global average.

In the Indian Ocean, the South Equatorial Current (SEC) forms the northern limb of the subtropical gyre, carrying fresh ITF water to the western basin. The SEC bifurcates east of Madagascar, forming the Northeastern and Southeastern Madagascar Currents (NMC and SMC, Fig. 1). Along the African shelf, the NMC further splits southward through the Mozambique Channel (MZC) and northwards as the East African Coastal Current (EACC). Further south, the SMC and MZC transport combine into the Agulhas Current (AC). The AC extension continues westwards beyond the African cape where it retroflects, returning most water eastwards to the Indian basin^33^, while a part of this water (~ 21Sv^34^) escapes into the South Atlantic as Agulhas Leakage.

**Figure 2:**
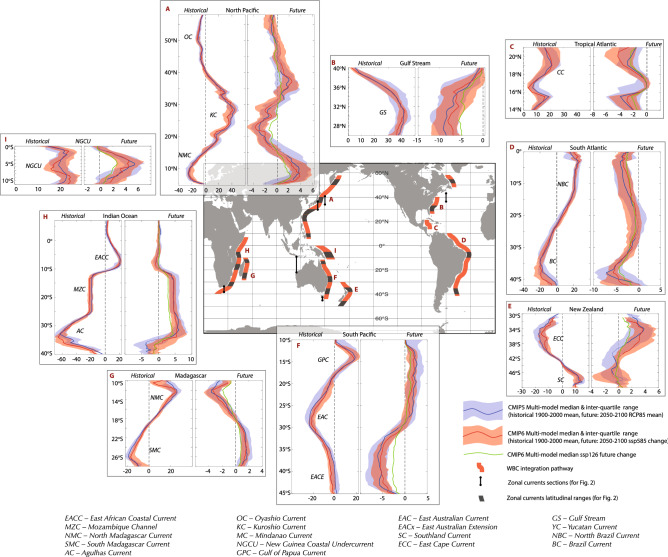
Historical meridional transport (left panels) and projected meridional transport change (right panels) by latitude along western boundaries shown in the map. Red/blue/green lines are multi-model median transport or transport change for CMIP6(SSP5-8.5)/CMIP5(RCP8.5)/CMIP6(SSP1-2.6) scenarios, associated shading indicates interquartile range (for high emission scenarios only). For projection panels lines are thickened where the multi-model median change is significant at the 95% level based on a two-sided Wilcoxon signed rank test. Black vertical lines and black polygons in the central map (along the WBC paths) show the location for the zonal and meridional transports presented in Fig. 3.

**Figure 3:**
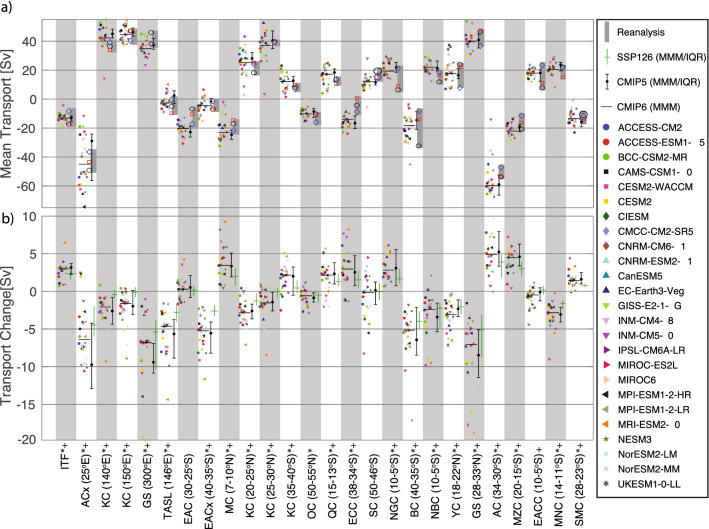
Upper panel: mean transport for selected currents averaged over the twentieth century for 25 CMIP6 models (see legend), with the horizontal black line indicating the multi-model median (MMM). The bar-whisker with black dots is the associated MMM and interquartile range for 28 CMIP5 models. Grey bars indicate the range in transports from three reanalysis products (ORAS5, GODAS and C-COR). Lower panel: associated change in transport between 2050–2100 and the twentieth century means based on SSP5-8.5 (symbols and horizontal black line), SSP1-2.6 (green bar and whisker) and RCP8.5 (black bar and whisker). Positive transports indicate northward or eastwards direction in both panels. */ + indicate transports for which the CMIP5/CMIP6 MMM projected change is significant at the 95% level based on a two-sided Wilcoxon signed rank test.

The EACC transport across the CMIP6 models (interquartile range: 16.5–19.9 Sv Fig. 2, Fig. 3a) is consistent with the observed 19 Sv peak near 5°S and lies within the broad range of reanalysis estimates (7.4–23 Sv). The simulated NMC (19.4–22.7 Sv) and SMC (−10.2 to −15.7 Sv) are generally weaker than the range of observations (27–48 Sv and 20– 30 Sv, respectively), based on multiple short-term estimates (Table S12), but span similar ranges to the reanalysis (Fig. 3). Conversely, the simulated transports through the MZC (17–24.6 Sv) are slightly stronger than observations and reanalysis (15-19 Sv and 11.8-21 Sv, respectively). The simulated MZC transport seasonality, which is maximum around austral autumn, agrees well with observations and reanalysis (Figure S1). Further south, the CMIP6 AC transport increases to 50.8–61.6 Sv near Africa’s southern tip, somewhat weaker than the observational (70–77 Sv) but overlapping the weaker reanalysis estimates (47.2–53.7 Sv). A recent 3-year campaign^34^ found AC transport at ~ 27°E to be strongest in austral summer and weakest in winter, although large interannual variability was evident. This seasonality is qualitatively consistent with the models and reanalysis, although the observed seasonal range ~ 15 Sv is considerably larger than in the models ~ 3 Sv (Figure S1).

Previous work^28^ showed a broad-scale projected slowdown of the south Indian Ocean circulation by the end of the twenty-first century in CMIP5 models. Their reported weakening of both the western boundary Agulhas system and eastern boundary Leeuwin Current system is consistent with our CMIP6 and CMIP5 results. There is near-unanimous agreement across CMIP6 for reduced transport for the MZC (3.3 to 5.3 Sv), SMC (0.9 to 1.8 Sv), NMC (−2.1 to −3.9 Sv) and AC (3.4–7.6 Sv) (Fig. 2, Fig. 3b). However, neither the CMIP5 nor CMIP6 models show a consistent change in the EACC. In contrast to the reduction in transport along much of the southern African coast, the westward flowing AC extension south of Africa intensifies in all models (−3.6 to −7.7 Sv at 25°E) – a ~ 15% strengthening.

### Atlantic Basin

At the northern extent of the South Atlantic subtropical gyre, the westward SEC bifurcates with most of its water entering the equatorward North Brazil Current (NBC)—responsible for large upper-ocean cross-equatorial heat transport^35^. The remainder flows southward from ~ 10°S forming the relatively weak Brazil Current (BC). In the North Atlantic, the poleward flow, partly fed by the NBC, follows the western boundary of Central America as the Caribbean and Yucatan Currents ultimately emerging via the Florida Straits to form the GS. The GS breaks away from the coast at ~ 40°N, feeding the north-eastward North Atlantic Current.

**Figure 4:**
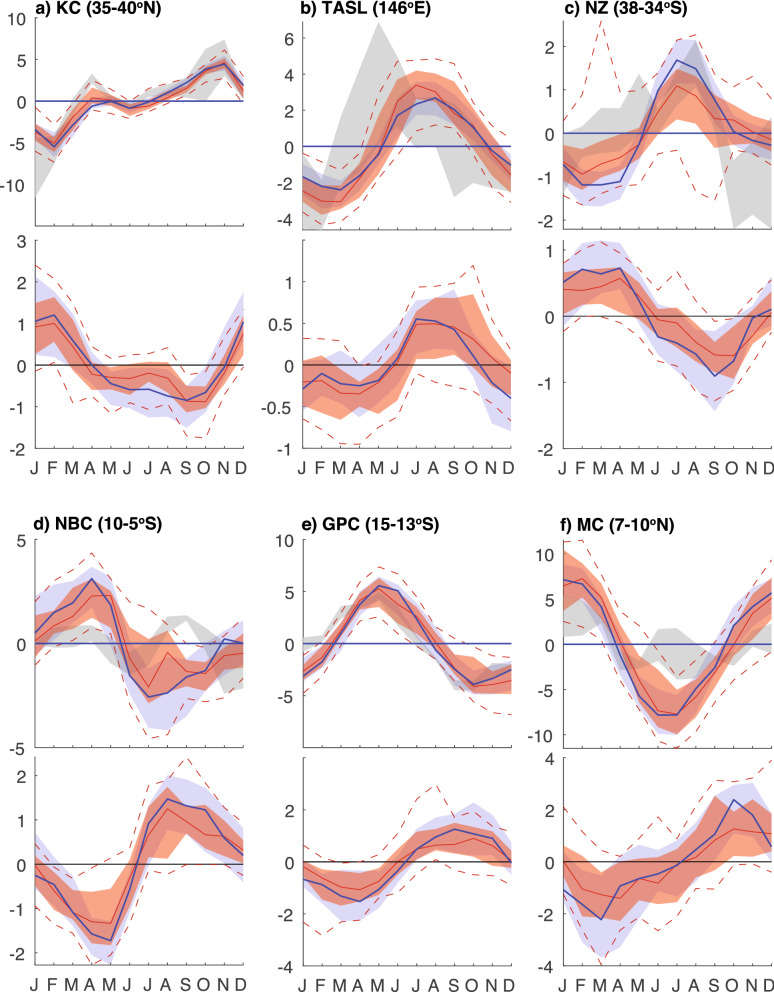
Seasonal cycle of mean transport (upper panels) and projected change (lower panels) for selected currents, where the annual mean transports have been removed. Red line/shading indicate multi-model median/interquartile range for CMIP6 models; blue line/shading/dashed line indicate multi-model median/interquartile range/interdecile range for CMIP5 models. Grey shading in upper panels indicates the range of three ocean reanalysis.

BC transport estimates from observations range from −19 and −23 Sv between 36 and 38°S. CMIP6 models generally simulate the maximum BC transport between about 35-40°S with values ranging from −13.9 to −25.8 Sv, which lies in the very broad range of reanalysis transports (−8.7 to −32.5 Sv). The observed NBC transport (23–26 Sv) is slightly underestimated by the ensemble (19–22 Sv, 5-10°S) with even weaker estimates from reanalysis (11.5–18 Sv). The models simulate maximum (minimum) transport in July-Aug (April–May, Fig. 4) (observed NBC seasonality estimates are not available at the latitudes examined). The BC and NBC forms at ~ 10°S^37^ just north of the basin-averaged zero wind-stress curl latitude. This bifurcation typically sits about 10° too far south in the CMIP models, in part related to a systematic southward bias in the model Atlantic wind field (Fig. 2, Figure S2).

In the Northern Hemisphere, the complex circulation of the Caribbean Sea and Gulf of Mexico is represented very differently across the coarse resolution models. Compared to observations, most models (and reanalysis) underestimate the LLWBC transport of the Yucatan Current (30 Sv) with a model range of 13.5–23.3 Sv (reanalysis: 8.5–25.6 Sv). The GS transport intensifies moving northwards (> 90 Sv) where it diverges from the coast, with the strongest transport occurring in boreal fall^38^. This northward intensification is absent in the models and reanalysis: northward transports peaks at 38 to 42.6 Sv between about 28-33°N (reanalysis: 37.1–46.7 Sv). The simulated winter intensification of the GS is consistent from the western margin to the extension region (Figure S1). However, the models are generally out of phase with the observations that indicate maximum transports during summer at 26.5°N^39^ and in the extension region^38^. While the reanalysis seasonality matches the models along the coast, there is poor agreement in the extension region.

In the Southern Hemisphere, the BC is projected to intensify (4.2 to 6.0 Sv), especially south of 30ºS, associated with an increased northward basin interior transport^27^. This intensification is consistent with intensified westerlies across the Indian Ocean basin (Figure S3, Figure S4), which can increase northward Ekman transport and intensify the Indian Ocean input to the Atlantic via Agulhas Leakage^31^.

Conversely, WBC transports weaken northwards of ~ 15°S. The cross-equatorial NBC flow is projected to weaken (−1.7 to −4.7 Sv). Similarly, the GS reduces at all latitudes with a −4.9 to −10.8 Sv (~ 15%) decrease around the GS maximum. These changes are poorly explained by surface wind changes and are likely associated with a weakened Atlantic Meridional Overturning Circulation (see below).

### Pacific Basin

In the South Pacific, the broad westward SEC bifurcates at the Australian margin forming the poleward EAC and equatorward Gulf of Papua Current (GPC). The EAC partially separates from the coast near 30ºS forming the Tasman Front, which continues southward to the east of New Zealand as the East Auckland Current and East Cape Currents (ECC). The remaining EAC water feeds a series of eddies that move southwards, forming the EAC extension and Tasman Leakage that provides a high-latitude pathway of water to the Indian Basin. The northward flowing GPC feeds the NGCU that exits the Northern Solomon Sea via multiple straits providing water to the subsurface Equatorial Undercurrent (EUC)^17^. In the Northern Hemisphere, the MC also feeds the EUC and forms the primary source of the ITF that transports warm water into the Indian Ocean^17^. Further north, the Kuroshio Current (KC) extends northwards from ~ 15ºN along eastern Japan, where it eventually separates and continues eastward.

The observed EAC transport reaches about −22 Sv at 27°S^40^, with maximum/minimum transport in austral winter/summer^41^. CMIP6 transports are generally similar in strength (−20.3 to −23.4 Sv) and seasonality (Figure S1) to observations. While the seasonality is similar for the reanalysis products, they tend to underestimate the transport (−7 to −17 Sv). The EAC extension transport (~ 7 Sv) and Tasman Leakage (~ 8 Sv) are, however, systematically underestimated in the models (−1.4 to −7 Sv and −0.9 to −4 Sv, respectively), with some models simulating an EAC extension with northward mean flow, related to a poor representation of regional winds^25^.

In the Northern Hemisphere, the KC intensifies from about 15 Sv at 18°N to over 20 Sv between 25 and 30°N. The CMIP6 models systematically overestimate the transport with an interquartile range of 30.1 to 44.2 Sv, which encompasses the reanalysis estimates of 38.6 to 39 Sv. Observations suggest that KC strength is weakest during winter, to the east of Taiwan^42^ while models and reanalysis display minimum transports earlier in autumn (Figure S1). Further north (28°N) observed transport is minimum in autumn^43^, while the models show no distinct seasonality. In the extension region, surface transport is weakest in winter/spring and strongest in summer/autumn^44^; while the model transports tend to peak in spring.

At low latitudes, observed MC transport estimates varies considerably (15 to 35 Sv, Table S12). Model and reanalysis transports lie within these estimates (−18 to −25.7 Sv and −14.6 to −21.8 Sv, respectively). Observational estimates of NGCU transport decrease from 29 Sv at 12°S to ~ 20 Sv at 1–2°N, with a large seasonality that is strongest (weakest) in austral winter (summer). In agreement with observations, the CMIP6 NGCU transports between 5–10°S are 17.4 to 25.5 Sv, with a large seasonality that peaks from July–October. Reanalysis transports are generally weaker (6–20.7 Sv) with seasonality matching the climate models. The inter-model spread in ITF transport is small compared to most other currents −11.9 to  −13.4 Sv (Fig. 3). This is slightly underestimated compared to the observed transport (15 Sv)^45^, with sub-1000 m transport accounting for ~ 0.5 Sv of this discrepancy. Flow strengths through the multiple ITF straits each have different seasonality, largely controlled by local monsoonal wind changes and remote oceanic forcing, resulting in a bimodal seasonality in the total ITF transport, peaking in January and July^45^. In the models, which do not simulate realistic flow through multiple straits, there is a single annual maximum around July, with a much larger (~ 10 Sv) seasonal range compared to observations, but consistent with reanalysis (Figure S1).

While the EAC core shows no consistent future change, the EAC extension and Tasman Leakage project large intensifications: −4.6 to −7.0 Sv (35–40°S) and −4.3 to −7.4 Sv (at 146°E), respectively. Previous studies have shown a negative low-frequency relationship between the EAC extension and the Tasman Front^46^. Consistent with this, most CMIP models project a weakening of the ECC that is fed by the Tasman Front. In the Northern Hemisphere, there is a projected weakening of the KC and Kuroshio extension across most models, but the changes are small relative to the mean transport.

In the tropics, both the GPC and NGCU project unanimous model intensifications: 0.6 to 2.8 Sv and 1.9 to 4.9 Sv, respectively. In contrast, the MC and the ITF (which the MC feeds) decrease in all models (2.3–5.6 Sv and 2.4–3.2 Sv, respectively). Similar LLWBC changes in the CMIP3 models were linked to projected basin-wide negative wind stress curl anomalies flanking the equator^26^. These curl anomalies are also evident in the CMIP5 and CMIP6 models (Figure S3). Conversely, the ITF weakening in CMIP5 models^25^ and in an eddy-permitting ocean projection^29^ could not be explained by regional wind changes. Instead, these studies found that the changes are related to a slowdown in deep ocean waters entering the South Pacific.

For the majority of currents examined across the basins, there is no significant difference in ensemble mean historical transports between CMIP5 and CMIP6. Only the Tasman Leakage and SMC demonstrate significantly different MMM transports (Table S13). In the case of the Tasman Leakage the MMM flow reverses direction from weakly eastwards in CMIP5 to weakly westward in CMIP6. This constitutes an important regional improvement although 20% of models still have spurious eastward flow in CMIP6 (compared to 57% for CMIP5). Only the MMM projected change in the AC extension transport is significantly different between model ensembles, with the CMIP6 suggesting a 40% smaller intensification compared to CMIP5.

### Seasonal changes

As described, many currents exhibit seasonal transport changes that are consistent across models (Figure S1). While seasonal timing is realistic for many currents, some simulated currents, for example the GS and Kuroshio system, poorly simulate the observed seasonal phase or amplitude. For some currents, comparison is hampered by uncertainties in the observed seasonality due to short observational records and large internal variability^37,47,48^. Both basin wide and local winds are important in setting transport seasonality, although the influence of remote winds may be lagged due to the slow propagation of ocean waves. The seasonal phase of wind stress curl in the models is broadly similar to the ERA5 reanalysis, although large discrepancies are evident, particularly near the boundaries of regions with strong seasonality differences (Figure S5a-c). We note that CMIP transports that show poor agreement with the observations or reanalysis seasonality (e.g. GS and KC extensions, NBC) are often associated with substantial biases in wind stress curl seasonality in the regions extending eastwards of the WBCs (Figure S5c).

A subset of currents also exhibits consistent projected changes in transport seasonality across both model generations (Fig. 4). In the South Indian Ocean, most models project a reduced seasonal cycle for the NMC and SMC (Figure S1). Likewise, in the South Atlantic, the models consistently simulate a substantial weakening of NBC seasonality. In the South Pacific, both the EAC extension and Tasman Leakage show an amplification in seasonality. Conversely, east of New Zealand, the seasonality of the ECC is projected to decrease. In the North Pacific, the seasonality in meridional transport where the KC separates from the coast shows consistent increases (decreases) in boreal winter (summer).

These currents with modified seasonality generally occur at latitudes where the phase of the projected wind stress curl seasonality also show large projected changes (except for the MC where the changes are just upstream of the MC latitudes examined; Figure S5e). These projected changes in wind stress curl seasonality are zonally oriented, occurring at transition zones where the historical wind stress curl seasonality changes rapidly with latitude (Figure S5b), suggesting that the changes are associated with a poleward expansion of the wind fields, a well-established consequence of anthropogenic climate change^10^, and their associated seasonality (Figure S5f.).

### Emergent constraints

For a subset of currents, there appears to be a significant inter-model relationship between historical and projected transports (Figure S6). These relationships may provide emergent constraints to narrow the uncertainty associated with the large spread in projections, although such constraints may be biased by common structural errors^49^. For example, for the EAC extension and Tasman Leakage, models that underestimate mean transport or that have flow in the wrong direction tend to project the largest increases in southward or westward flow, respectively. Given observed EAC extension transports (~ 7 Sv, Table S12), a more moderate future change (~ 5 Sv) may therefore be more credible than the more extreme changes projected by some models. Similarly, given an observed mean transport of ~ 15 Sv, it is likely that the ITF decrease would be 3-4 Sv rather than the more extreme model estimates.

### Connections to surface wind changes

Neglecting friction, non-linear processes and interactions with deep ocean circulation, the depth-integrated meridional transport away from the western boundaries can be related to gradients in the surface wind field via Sverdrup dynamics^7^. In particular, a positive (negative) wind stress curl drives northward (southward) flow in the ocean interior. WBCs provide a return flow for much of this meridional transport and a significant part of the inter-model differences in the historical mean WBC transport can be related to differences in interior transport (Table 1, Figure S7). The offset between the WBC-interior regression lines and the one-to-one line in Figure S7 for certain currents relate to inter-basin leakage or flow compensation in the deep ocean as part of the overturning circulation. For example, the ~ 10 Sv offsets for the EAC, EAC extension, GPC and NGCU result from a leakage of water via the ITF. The offset is ~ 5 Sv less than the ITF transport as there is also a net upwelling into the upper Pacific from below 1000 m. The offsets for the Atlantic basin currents, including the GS and NBC, result from the deep return flow below 1000 m.

**Table 1:** Correlation between: interior (to the east of the WBC) and WBC transport (column 2), interior and derived Sverdrup transport (column 3), WBC and Sverdrup transport (column 4). Associated correlations for projected changes shown in columns 4, 5 and 6. Outliers (values exceeding 3 × scaled median deviations) are removed prior to the calculation of correlations. ^+1^EAC extension includes transport to the east of New Zealand. ^+2^MAD includes the WBC to the east and west of Madagascar. Scatter plots of Interior vs WBC and interior vs Sverdrup transport for the combined CMIP5 & 6 ensemble shown in *Figure S7*. Bold correlations indicate significant correlations at 95% level, based on Spearman Rank correlation.

	Historical			Projected change		
	Interior vs WBC	Interior vs Sverdrup	WBC vs Sverdrup	Interior vs WBC	Interior vs Sverdrup	WBC vs Sverdrup
EAC core	** − 0.91**	**0.76**	** − 0.68**	** − 0.81**	**0.49**	** − 0.58**
EAC extension^+1^	** − 0.87**	**0.85**	** − 0.72**	** − 0.96**	**0.8**	** − 0.7**
MC	** − 0.82**	**0.53**	** − 0.66**	** − 0.91**	**0.6**	** − 0.66**
KC South	** − 0.93**	**0.47**	** − 0.34**	** − 0.93**	**0.76**	** − 0.79**
KC Central	** − 0.98**	0.2	** − 0.15**	** − 0.94**	**0.41**	** − 0.35**
KC North	** − 0.77**	**0.62**	** − 0.46**	** − 0.98**	**0.76**	** − 0.77**
GPC	** − 0.95**	**0.49**	** − 0.45**	** − 0.86**	**0.83**	** − 0.6**
NGCU	** − 0.76**	**0.75**	** − 0.75**	** − 0.92**	**0.89**	** − 0.76**
BC	** − 0.95**	**0.34**	** − 0.32**	** − 0.78**	0.22	− 0.05
NBC	** − 0.42**	**0.84**	** − 0.38**	− 0.2	**0.86**	** − 0.35**
GS	** − 0.54**	**0.57**	− 0.04	** − 0.78**	**0.48**	** − 0.35**
AC	** − 0.8**	**0.55**	− 0.26	** − 0.85**	**0.42**	** − 0.29**
MAD^+2^	** − 0.58**	**0.61**	** − 0.45**	** − 0.58**	**0.57**	** − 0.31**
EACC	** − 0.75**	**0.53**	** − 0.48**	** − 0.8**	**0.72**	** − 0.76**

For many currents intermodel difference in interior transports can be explained to some degree by differences in the surface wind field via Sverdrup dynamics (Table 1). As a result, up to 50% of the intermodel variance in WBC transports can be related to differences in the surface winds. Other factors, including different overturning rates, different inter-basin transports and non-linear dynamics must be invoked to explain the wide range of mean WBC transports.

Similarly, a significant fraction of intermodel projected WBC differences can be related to changes in surface wind stress curl, for most WBCs investigated (Table 1). In general, WBC whose mean differences are well explained by differences in their surface winds tend to be those whose projected transport differences are also well explained by differences in surface wind changes. The particularly poor relationship noted for the BC probably relates to the fact that the Sverdrup calculation becomes poorly defined as the eastern boundary lies at the southern tip of Africa. Other weak relationships in the Atlantic likely stem from large projected changes in the Atlantic overturning circulation^50^. Indeed, projected NBC decreases in CMIP5 are largely compensated by a weakening of North Atlantic Deep Water transport^27^.

### Near-surface transport

WBCs affect the distribution of marine species via the dispersal of early-life stages and modulation of local thermal regimes^24,51^. However, ecosystem impacts will be most sensitive to near-surface circulation changes within the euphotic zone where most marine life thrives. As such, we also examine WBC transport changes in the top 100 m of the water column.

For most currents examined, the change in the near-surface flow is of the same sign as the 1000 m integrated transport. An exception is the KC system, where the full-depth WBC is projected to weaken slightly along most of its length, while the near-surface flow is projected to intensify weakly north of 25°N (Figure S8, Figure S9). Previous work suggested that this intensification is associated with differences in warming rates across the KC, leading to an enhanced baroclinic flow^52^. As a tight connection between the state of the KC and the regional marine food webs has been documented^53^, this surface intensification may have consequences for the ecosystem. In contrast, the full-depth MC, which is projected to weaken, typically intensifies near the surface south of 7°S.

In general, when the direction of a WBC is aligned with the warming signal (e.g. in the subtropics), a poleward intensified WBC will assist species dispersal at poleward range edges. In contrast, when the WBC flow opposes climate change velocities (e.g. in the tropics), strengthening would hinder dispersal at the poleward edges with greater propagule dispersal at the warming, equatorward edges^24^. Weakened/strengthened WBCs are likely to directly modify larval transport and thermal regimes, affecting rates of poleward range shifts^51^. In addition, other more subtle changes such as WBC broadening or modified coastal retention or dispersal pathways may also impact marine life^24^.

## Summary and conclusions

We have examined projected changes to the upper ocean western boundary circulation and associated currents. Many circulation features examined exhibit consistent projected changes across climate models (Fig. 1) despite large differences in model structure, e.g. bathymetry (Figure S10), resolution, parameterisation of unresolved processes. This suggests common large-scale drivers of change. Comparing CMIP5 and CMIP6, we found little robust differences in either mean current strengths (including their seasonality) or their projected changes between the model generations. A significant fraction of the inter-model differences in the projected changes can be related to differences in projected wind stress curl changes through linear dynamics. As such, the strong similarity between regional surface wind changes from CMIP5 and CMIP6 (Figure S3, Figure S4) would suggest little inter-generational difference in the circulation projections. However, other model differences including inter-basin exchanges (e.g. via the ITF) and overturning circulation changes (e.g. the slowdown of the Atlantic overturning circulation) will also be important.

Basin-scale changes in the wind field can drive changes to the WBCs on the multi-decadal timescales required for planetary waves to propagate across basins and establish a steady state. However, in a few regions, we also found consistent seasonality changes. These changes likely result from regional rather than basin-scale surface winds change, particularly at higher latitudes, given the months to years for oceanic planetary waves triggered by remote winds to propagate to the western boundaries or from changes to the overturning circulation. While we did not perform a detailed analysis, currents with projected seasonality changes were often located at or to the west of regions with substantial projected wind stress curl seasonal phase changes. These changes likely relate to seasonal wind regimes shifting poleward.

Such seasonal changes may be particularly relevant for understanding how future climate will impact marine life as many key biological processes (e.g. spawning, migrations) exhibit marked phenology. Circulation driven changes in temperature seasonality are also likely to be important. For example, winter, rather than annually averaged, temperatures in the Gulf Stream best explains the inter-annual variation in species’ distribution and biomass^54^.

It is interesting to note that the projected changes along the WBCs do not typically align with the historical-mean currents: it would be wrong to conceptualise these circulation changes as increases or decreases in the speed of a conveyor belt. For example, the core EAC exhibits little future change, while the EAC extension shows large consistent increases in transport. The main Agulhas system shows a similar projected decrease in transport along its length, while south of Africa, the AC extension intensifies.

A major criticism of coarse resolution climate models is that WBCs are poorly simulated e.g. flow speeds are too weak and too wide. Furthermore, WBCs are usually associated with strong mesoscale eddy activity not resolved in CMIP models. Despite this, simulations carried out in eddy-resolving or eddy-permitting ocean models find projected transport changes in the Agulhas system^31^, EAC system^30,55^, ITF^29^ and globally^24^ that are broadly consistent with the CMIP models. This suggests that mesoscale processes are of secondary importance and that changes in basin-scale winds^30,31^, or deep circulation in some cases like the ITF^25,29,56^, explain a large component of the projected changes in transport. Indeed, while mesoscale processes affect certain characteristics of WBCs, transport projections for these narrow WBCs may be more reliable than projections for other variables that depend critically on local processes. This is not to say that other aspects of WBC changes may be unrealistic, including changes in current speeds or changes in eddy activity. Indeed, previous eddy-resolving ocean-biogeochemical model simulation^55^ found projected increases in Tasman Sea eddy activity, nutrient pumping and productivity associated with the enhanced EAC extension, contrary to projected changes in a coarse resolution control model. It will be important to examine how the projections from coarse resolutions models discussed here differ from the high-resolution climate projections from HighResMIP^57^ in progress (at the time of writing no output is available for the second half of the twenty-first century).

These future circulation changes could have important implications for ecosystems and the fisheries that rely on them. The intensification of the EAC extension has already caused profound shifts in the distribution of fish^58^ and almost complete giant kelp forests losses, with major impacts on valuable fisheries^59^. Conversely, new fisheries are emerging as a result of range expansions of high-value species^60^. Future intensified warming and circulation increases in this region are likely to exacerbate these changes, with implications for conservation and management^6^.

Climate-associated shifts in stocks of commercial species, such as those related to WBC changes, pose major governance challenges as species move beyond national and international boundaries, which can lead to conflict over shared resources^61^. Improving our understanding of future WBC changes will be key to credible forecasts of the distribution of commercially-important species, enabling the development of governance solutions^62^. While CMIP-class models may provide credible forecasts of transport changes, the associated changes in current speeds, eddy activity and cross shelf processes will require the use of global or regional models that can represent mesoscale and smaller processes.

## Materials and methods

We use zonal and meridional ocean velocity, wind stress and sea surface temperature from 28 CMIP5 (Table S1) and 25 CMIP6 (Table S2) models. Meridional western boundary transports are integrated from the surface to 1000 m (or to 100 m) and from the coast to 7° offshore (15° offshore for the NGCU) except where the boundary currents do not run close to the coast. In such cases, we manually identified the western edge of WBCs by an examination of the historical mean meridional transport for each model. Unlike in the real ocean, WBCs in coarse resolution climate models can have widths of several hundreds of kilometres. Transports are relatively insensitive to the boundary current width used as WBC flow is generally strongest towards the shore (Figure S11). Zonal transports are calculated at selected longitudes to capture the flow associated with WBC extensions (e.g. AC extension, Tasman Leakage and the ITF). The northern and southern extents of the currents were manually identified based on examination of the historical mean meridional transport (for approximate locations for all sections, see Fig. 2).

We compare transports averaged over 1900 to 2000 (*historical* scenario) with averaged transports for 2050 to 2100 (*RCP8.5* scenario for CMIP5; *SSP5-8.5* for CMIP6). These long timeframes ensure that differences are primarily associated with the imposed anthropogenic forcing, as much of the intrinsic variability is removed when considering the long-term average. Given the large spread in the number of available ensemble members, our results are based on a single ensemble member from each model. Testing with selected models indicates negligible differences in climatological transports based on different ensemble members. We use the ‘business as usual’ scenario as it provides the highest signal to noise ratio. Results from lower emission scenarios produce commensurately smaller projected changes; multi-model median results for SSP1-2.6, which is commensurate with the policy relevant 2°C warming by the end of the century, are presented for comparison for a subset of analysis (Fig. 2, Fig. 3, Figure S8, Figure S9). In the manuscript text, we quote interquartile transport ranges across the CMIP6 ensemble, individual model transports for the upper 1000 m (and 100 m) and interquartile ranges for CMIP5 and CMIP6 are shown in Fig. 2, Fig. 3 and Table S1-10.

The significance of projected changes was tested using a non-parametric two-sided Wilcoxon signed rank test. Inferences based on these statistical tests likely overestimate the level of significance since models share common components and are so not truly independent of each other.

Where possible model transports are compared to estimates from three ocean reanalysis products (Table S11): Ocean Reanalysis System 5 (ORAS5)^63^, Global Ocean Data Assimilation System (GODAS)^64^ and CMCC Global Ocean Reanalysis System (C-GLORS)^65^; and observations cited in the literature (Table S12). While the latter is not a like-for-like comparison as locations, integration depths and time periods vary, this provides a useful order of magnitude evaluation of the models. Model winds are compared against the ERA5 reanalysis^66^.

All figures generated in MATLAB 2020b update 3. Labels, legends and schematics were done using Adobe Illustrator 25.2. Coastlines generated using m_map toolbox^67^.

## Supplementary Information

Supplementary Information
