# Supplementary material for: Future changes to the upper ocean Western Boundary Currents across two generations of climate models
Source: Sci Rep. 2021 May 5;11:9538. doi: 10.1038/s41598-021-88934-w (PMC8099859; doi:10.1038/s41598-021-88934-w)
Supplement: Supplementary file 1 — Supplementary Information [file 41598_2021_88934_MOESM1_ESM.pdf]

**Supplementary Information**

# Future Changes to the upper ocean Western Boundary Currents across two generations of climate models


*Alex Sen Gupta[1,2,3]
Annette Stellema[1,2,3]
Gabriel Pontes[4]
Andréa S. Taschetto[1,2]
Adriana Vergés[3,5]
Vincent Rossi[6]

1. Climate Change Research Centre, University of New South Wales, Sydney, Australia
2. Australian Research Council Centre of Excellence for Climate Extremes, University of New South Wales, Sydney, Australia
3. Centre for Marine Science and Innovation, University of New South Wales, Sydney, Australia
4. Institute of Oceanography, University of São Paulo, Brazil
5. Centre for Marine Science & Innovation and Evolution & Ecology Research Centre, School of Biological, Earth and Environmental Sciences, UNSW Australia, Sydney, New South Wales, Australia
6. Mediterranean Institute of Oceanography (UM 110, UMR 7294), CNRS, Aix Marseille Univ., Univ. Toulon, IRD, 13288, Marseille, France

## Supplementary Figures

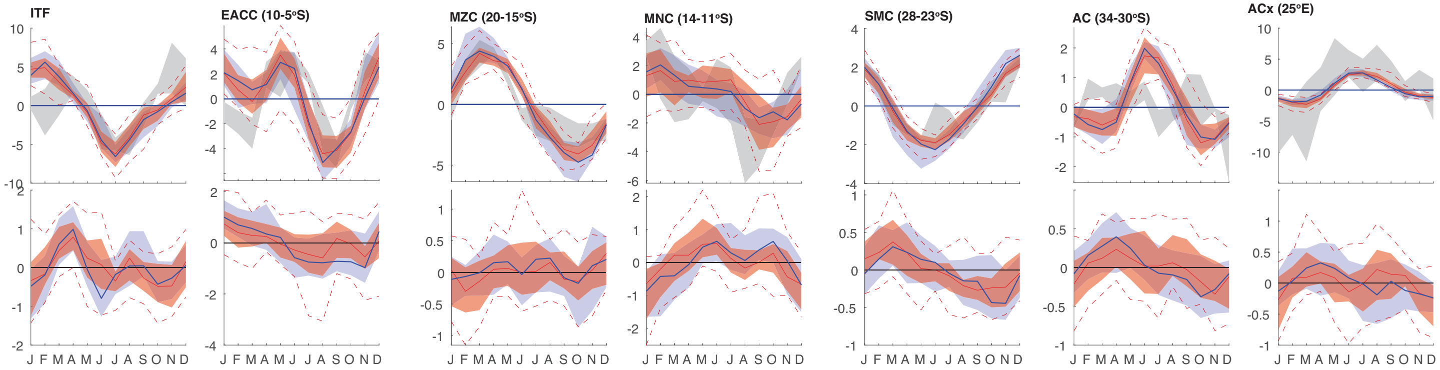


*Figure S1 Seasonal cycle of mean transport (upper panels) and projected change (lower panels) for selected currents, where the annual mean transports have been removed. Red line/shading indicate multi-model median/ interquartile range for CMIP6 models; blue line/shading/dashed line indicate multi-model median/ interquartile range /interdecile range for CMIP5 models. Grey shading in upper panels indicates range of three ocean reanalysis products. (Indian Ocean)*

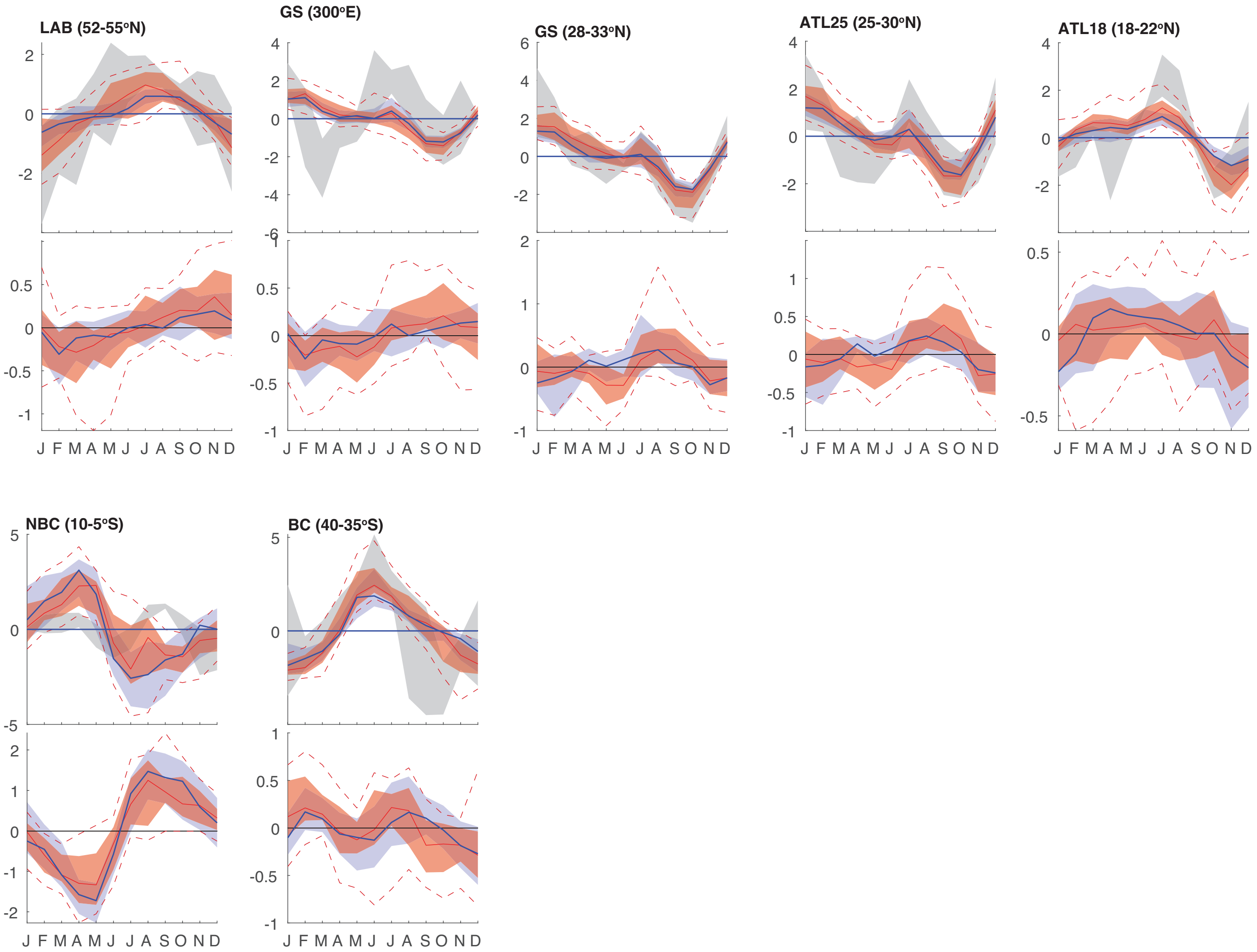


*Figure S1 continued (Atlantic Ocean)*

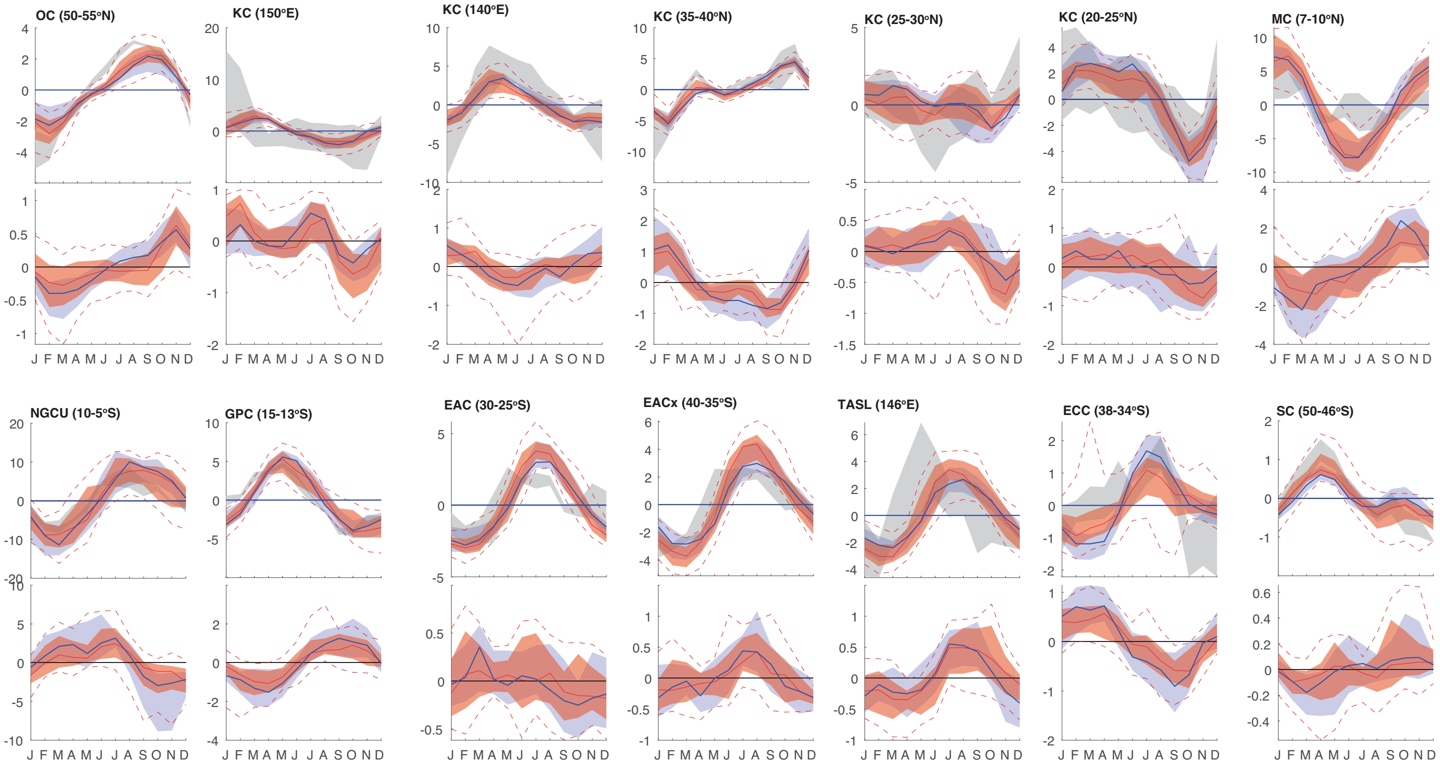


*Figure S1 continued (Pacific Ocean)*

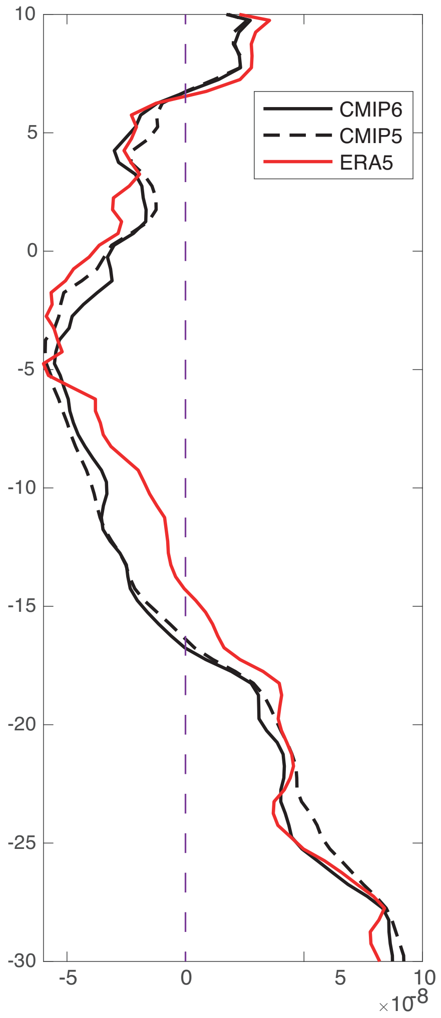


*Figure S 2 Atlantic basin averaged wind stress curl, for CMIP5, CMIP6 and ERA5*

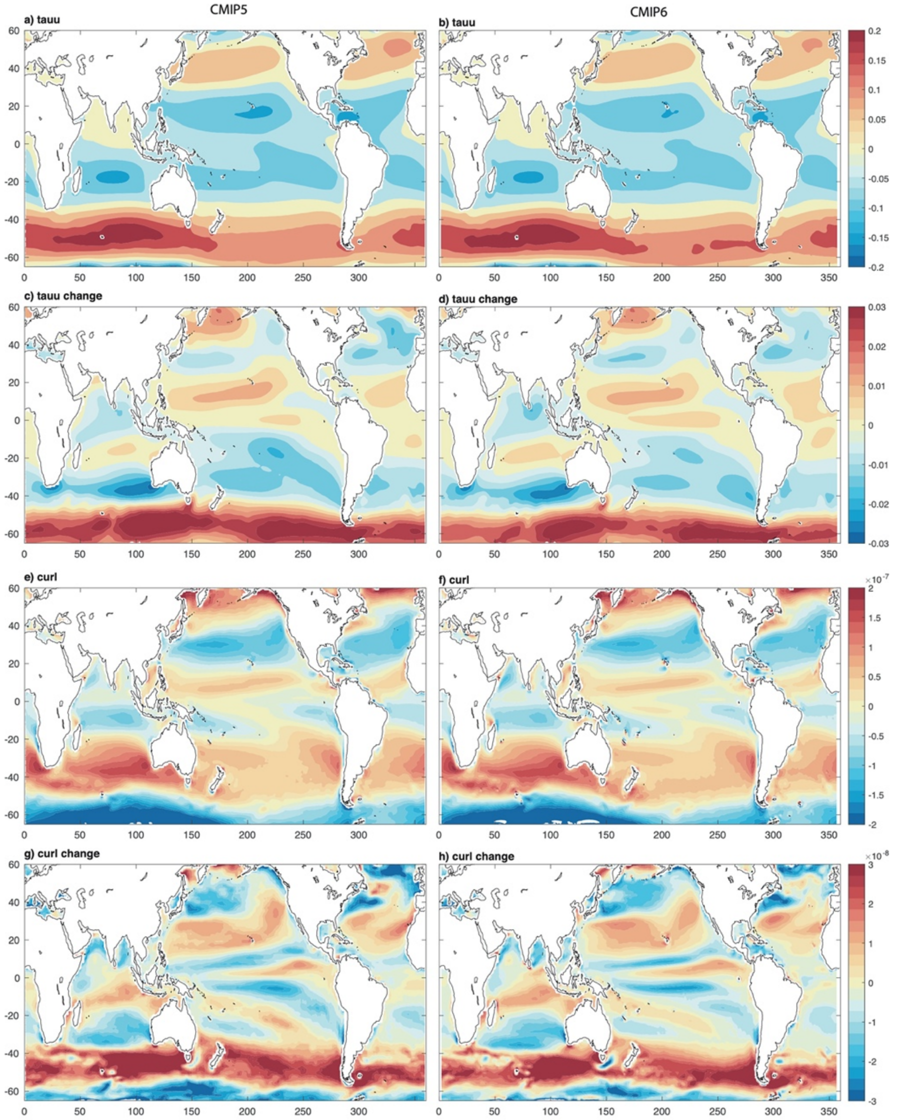


*Figure S 3* Multi-model mean a,b) historical zonal wind stress and c,d) projected change; e,f) historical wind-stress curl and g,h) projected change for CMIP5 models (left) and CMIP6 models (right)

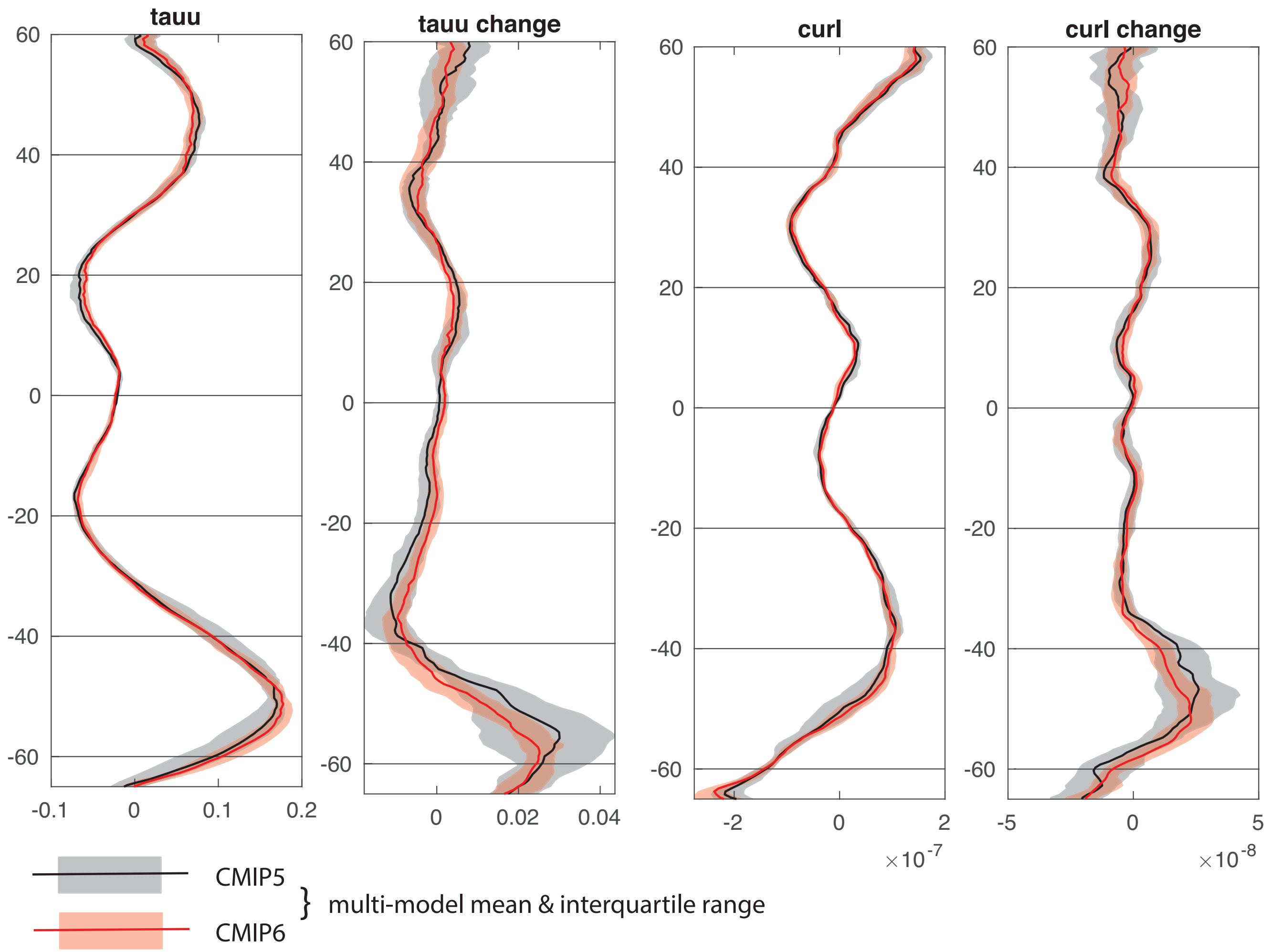


*Figure S 4* Multi-model median, zonally averaged a) zonal wind stress, b) zonal wind stress change, c) wind stress curl and d) wind stress curl change.

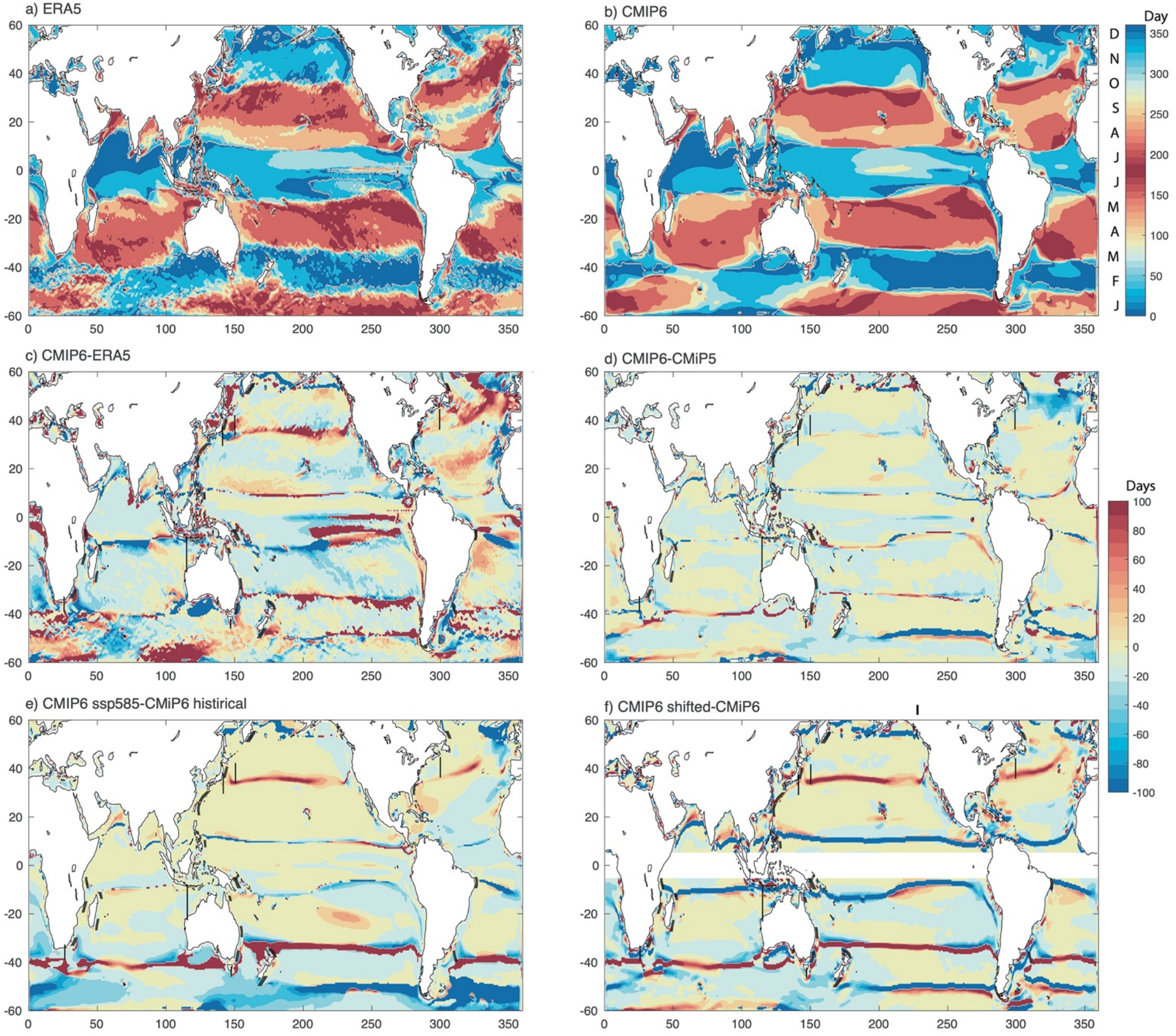


*Figure S 5 Timing (day of year) of the peak in the seasonal cycle in wind stress curl (based on the best-fit annual, 1st harmonic) for a) ERA5 and b) historical CMIP6 MMM. Subsequent panels show the timing difference between c) ERA5 and CMIP6, d) CMIP5 and CMIP6, e) historical and SSP585 projections and f) CMIP6 and CMIP6 with northern and southern hemisphere wind fields displaced 2° poleward.*

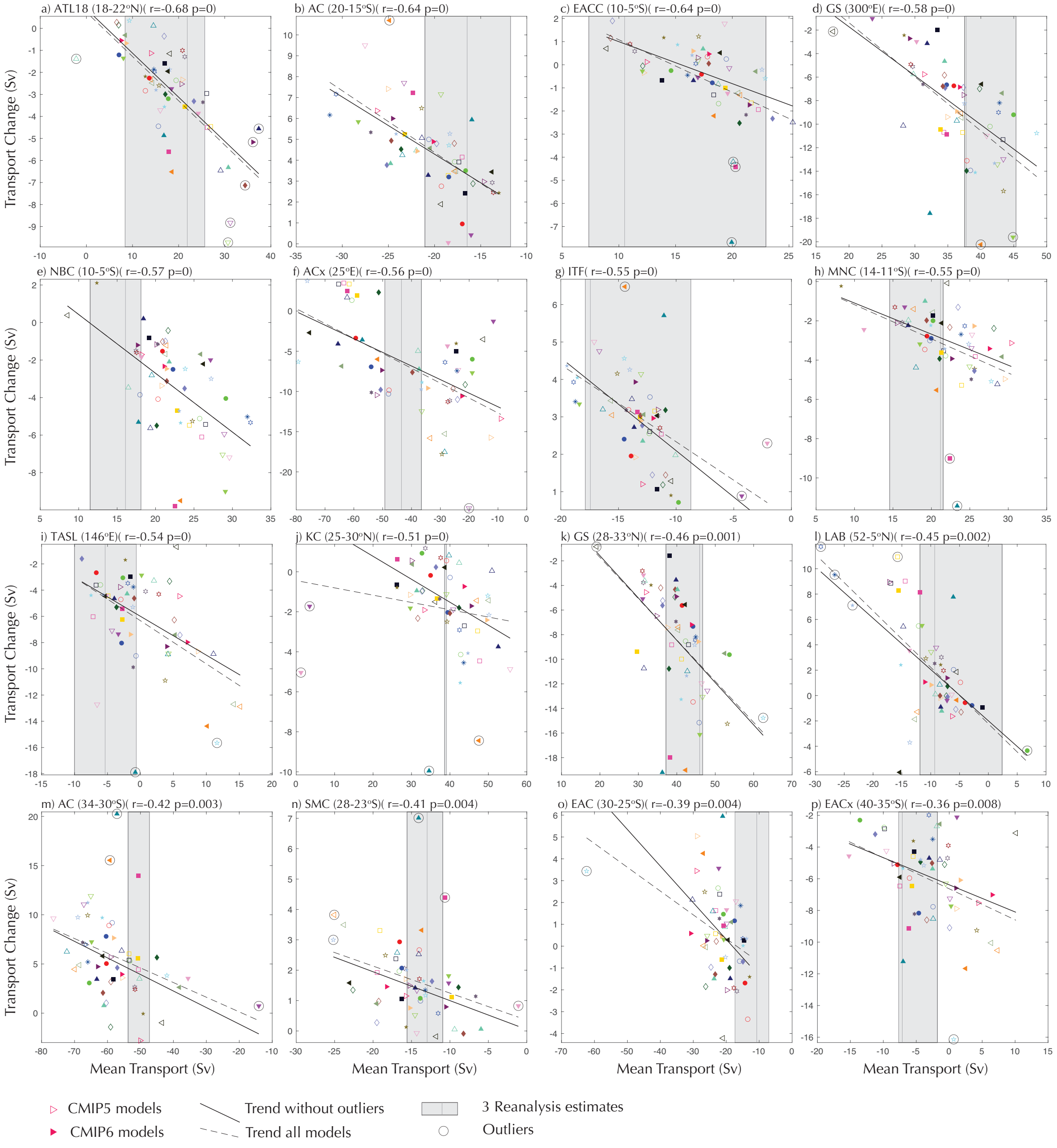


*Figure S 6* Emergent constraints. Mean historical transport versus projected transport change from CMIP6 (filled) and CMIP5 (unfilled) models, for currents with significant relationships ($p<0.01$, assumes independent models). Regression line for combined model ensemble superimposed - all models (dashed), outliers removed (solid). Transports estimated from three reanalysis superimposed (grey band/vertical lines). Correlation coefficient (r) and associated p-value shown in title. Model outliers (circled; values > three scaled median absolute deviations) are removed prior to calculation of correlations and regression lines.

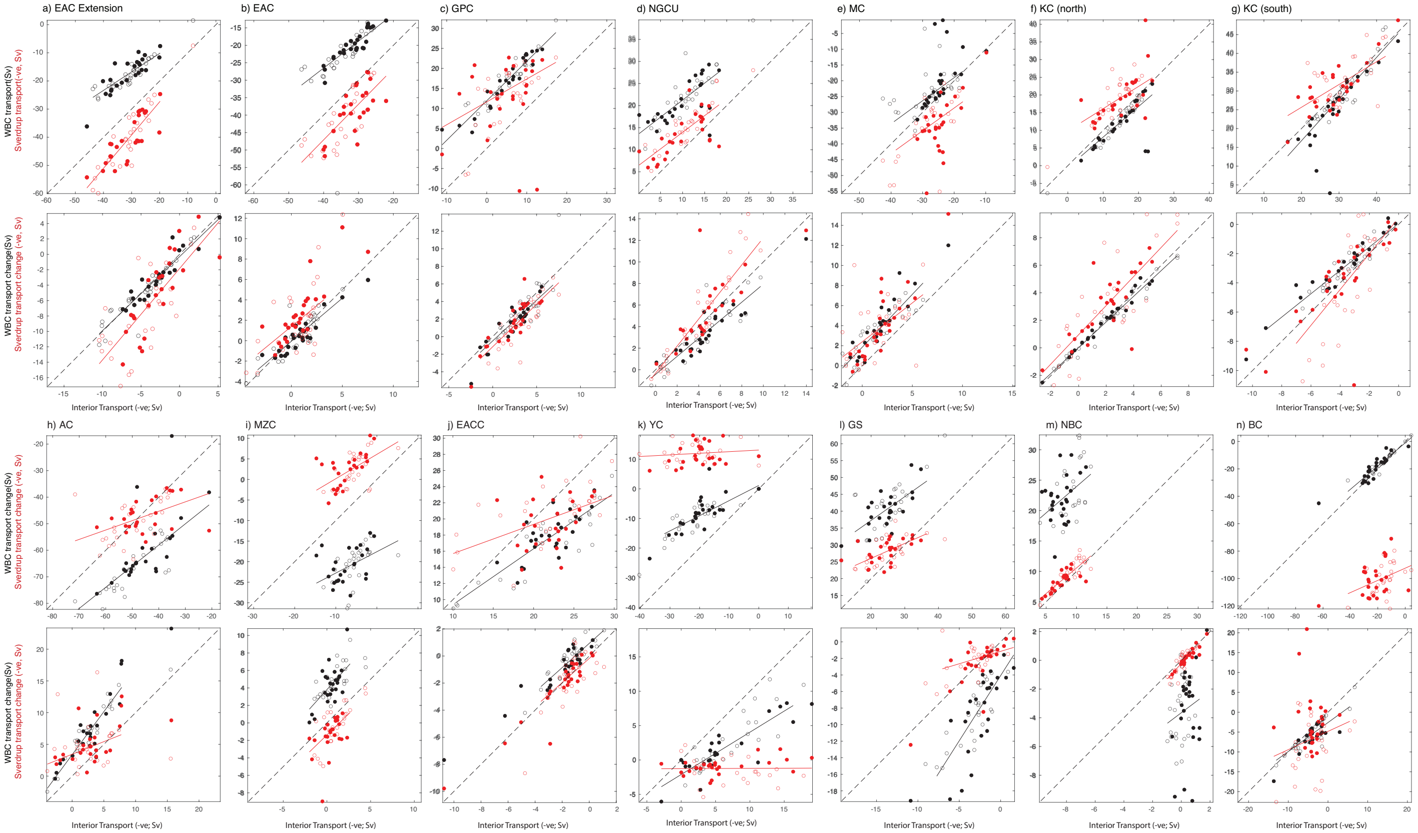


*Figure S7* Rows 1 and 3: interior transport (x-1) versus WBC transport (black) or derived Sverdrup transport (red, x-1) for CMIP5 (open circles) and CMIP6 (filled circles) for selected currents shown on Figure 2 (upper 1000m integrated transport). Rows 2 and 4 associated projected changes. Solid lines show linear best fit to the combined CMIP5+6 values. One-to-one line: dashed. Outliers (values exceeding 3x scaled median deviations) are removed prior to calculation of trend lines.

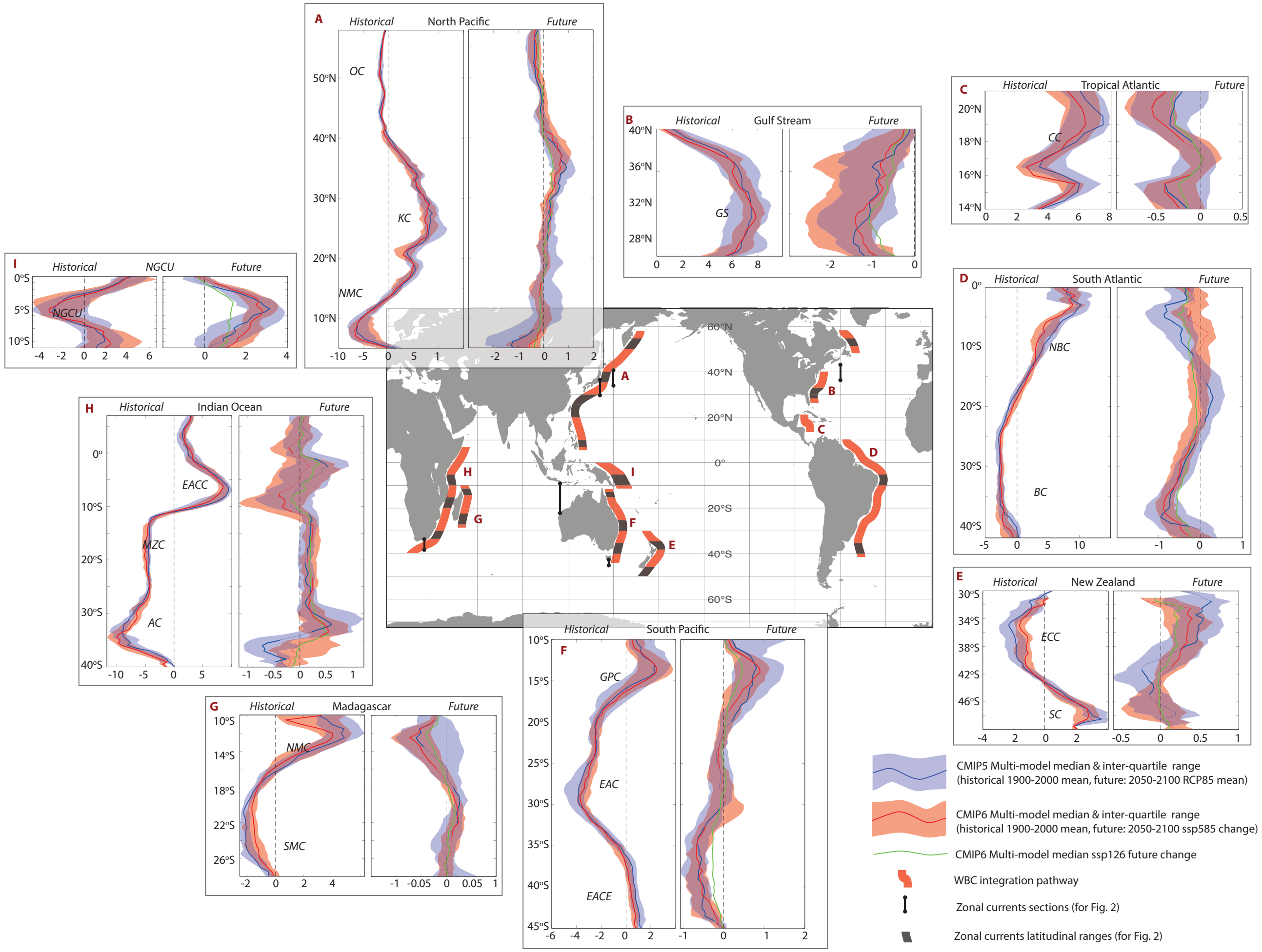


*EACC – East African Coastal Current*
*MZC – Mozambique Channel*
*NMC – North Madagascar Current*
*SMC – South Madagascar Current*
*AC – Agulhas Current*

*OC – Oyashio Current*
*KC – Kuroshio Current*
*MC – Mindanao Current*
*NGCU – New Guinea Coastal Undercurrent*
*GPC – Gulf of Papua Current*

*EAC – East Australian Current*
*EACx – East Australian Extension*
*SC – Southland Current*
*ECC – East Cape Current*

*GS – Gulf Stream*
*YC – Yucatan Current*
*NBC – Nortth Brazil Current*
*BC – Brazil Current*

*Figure S 8 As per Figure 2, but for 0-100m integrated transport*

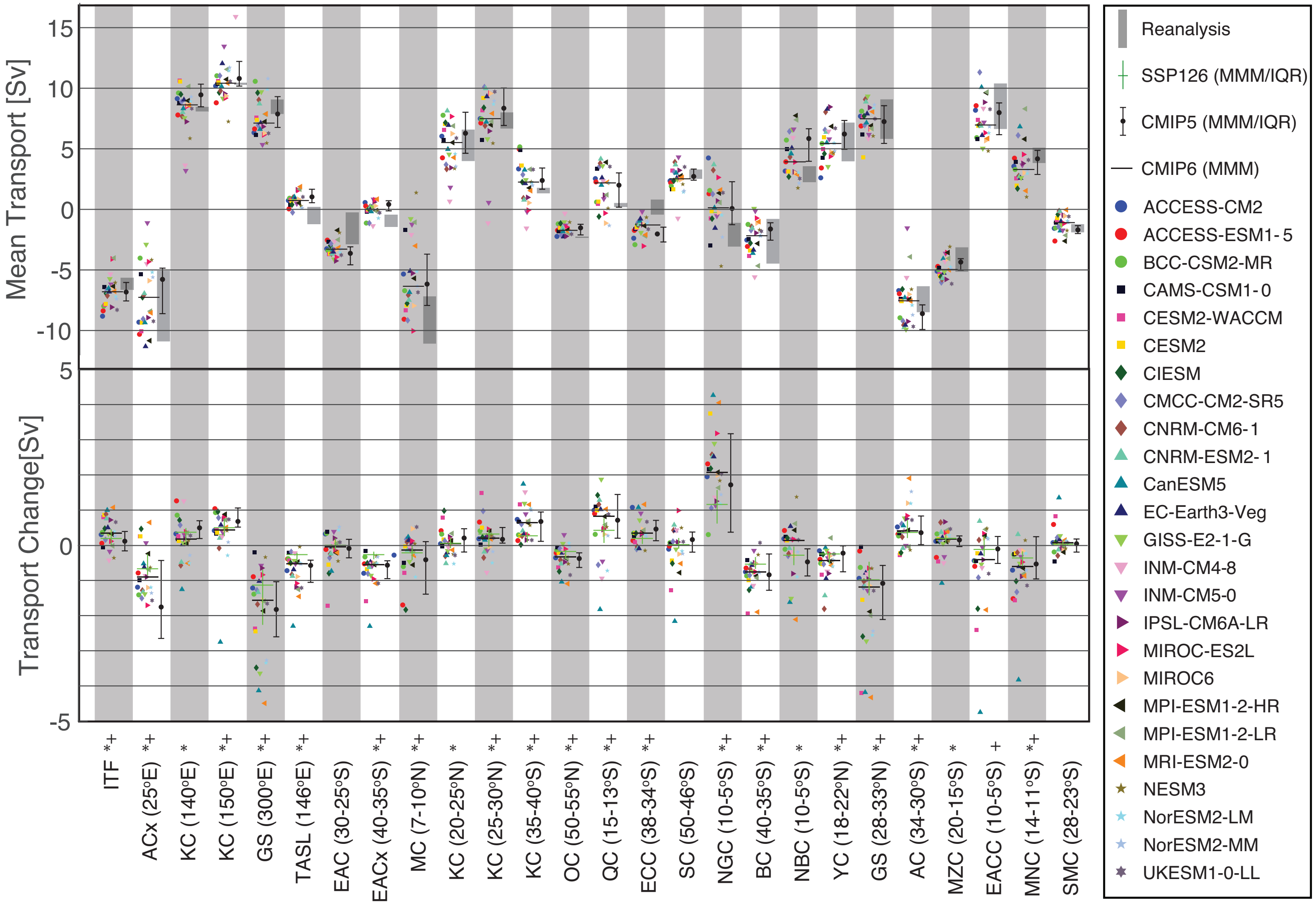


*Figure S 9 As per Figure 3, but for 0-100m integrated transport*

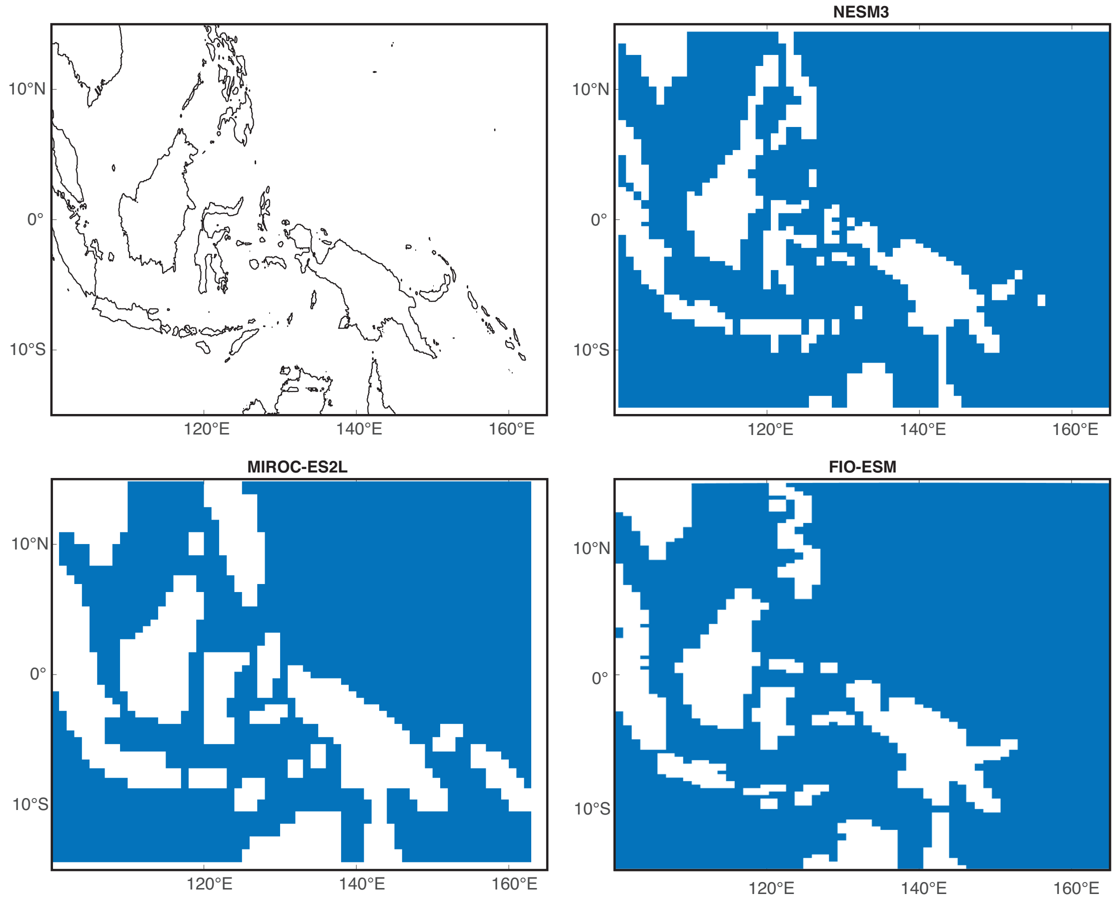


*Figure S 10 Model representation of coastlines. Land-ocean masks over the maritime continent for three CMIP6 models (NESM3, MIROC-ES2L, FIO-ESM) in comparison to the coastline derived from the ETOPO2 dataset[1] (top left).*

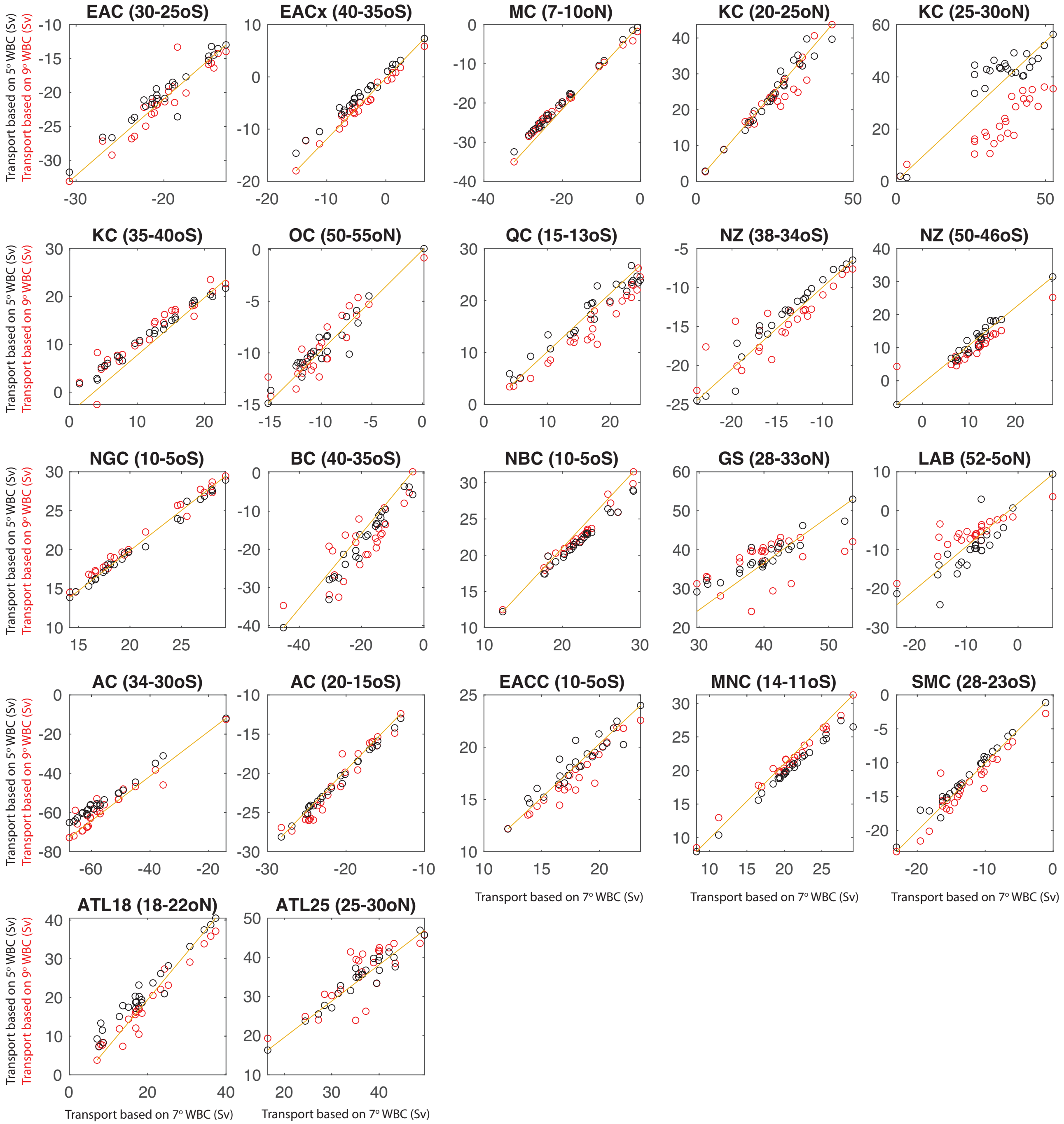


*Figure S 11 Transport sensitivity test. Transport calculated using 7° threshold vs transport calculated using 5° (red circles) or 9° (black circles) threshold for major currents. One-to-one line superimposed.*

*Table S1 0-1000m integrated historical transports for currents described in the text (CMIP5 models)*

| | ACCESS1-0 | ACCESS1-3 | CCSM4 | CESM1-BGC | CESM1-CAM5-1-FV2 | CESM1-CAM5 | CMCC-CESM | CMCC-CMS | CMCC-CM | CNRM-CM5 | CanESM2 | FIO-ESM | GFDL-CM3 | GFDL-ESM2G | GFDL-ESM2M | HadGEM2-AO | IPSL-CM5A-LR | IPSL-CM5A-MR | IPSL-CM5B-LR | MIROC-ESM-CHEM | MIROC-ESM | MIROC5 | MPI-ESM-LR | MPI-ESM-MR | MRI-CGCM3 | MRI-ESM1 | NorESM1-ME | NorESM1-M | **Median** | **25th percentile** | **75th percentile** | **Reanalysis mean** |
|---|---|---|---|---|---|---|---|---|---|---|---|---|---|---|---|---|---|---|---|---|---|---|---|---|---|---|---|---|---|---|---|---|
| ITF | -12.3 | -14.1 | -12.3 | -12.3 | -11.3 | -11.9 | -11.2 | -12.1 | -10.9 | -10.1 | -16.4 | -13.8 | -13.0 | -17.1 | -16.7 | -11.6 | -12.9 | -13.5 | -10.4 | -15.6 | -15.6 | -15.1 | -14.5 | -14.0 | -11.6 | -11.4 | -18.9 | -18.7 | **-13.0** | **-15.2** | **-11.6** | **-14.7** |
| ACx(25°E) | -48.0 | -47.8 | -60.7 | -65.3 | -63.5 | -61.6 | -21.4 | -24.0 | -25.6 | -37.7 | -28.5 | -62.3 | -36.4 | -23.8 | -20.1 | -52.1 | -9.0 | -12.5 | -15.1 | -28.4 | -33.5 | -29.4 | -79.3 | -76.2 | -26.7 | -27.4 | -28.4 | -24.2 | **-29.0** | **-54.2** | **-24.2** | **-43.1** |
| KC(140°E) | 41.7 | 40.3 | 44.5 | 44.5 | 44.9 | 45.2 | 51.0 | 44.4 | 47.7 | 42.9 | 47.6 | 44.6 | 50.6 | 49.0 | 48.3 | 34.5 | 45.8 | 46.0 | 45.8 | 51.7 | 50.1 | 40.8 | 55.1 | 48.3 | 39.6 | 39.3 | 41.4 | 42.9 | **45.0** | **42.6** | **48.3** | **36.0** |
| KC(150°E) | 40.9 | 43.1 | 41.7 | 41.7 | 45.1 | 45.1 | 48.6 | 47.1 | 45.5 | 46.3 | 49.3 | 58.9 | 49.3 | 43.9 | 46.2 | 42.6 | 46.6 | 45.3 | 50.6 | 56.2 | 54.9 | 45.0 | 53.3 | 49.0 | 46.1 | 46.7 | 42.2 | 43.0 | **46.2** | **43.7** | **49.1** | **44.3** |
| GS(300°E) | 38.4 | 37.9 | 41.4 | 43.4 | 34.4 | 37.2 | 39.8 | 37.4 | 34.5 | 29.8 | 36.4 | 28.2 | 42.5 | 40.9 | 43.4 | 37.4 | 31.4 | 34.9 | 17.6 | 37.1 | 37.0 | 43.4 | 48.5 | 37.5 | 29.7 | 29.4 | 42.7 | 42.8 | **37.4** | **34.5** | **41.7** | **40.2** |
| TASL(146°E) | -0.7 | -3.0 | -6.1 | -6.7 | -7.2 | -4.9 | 4.4 | 6.0 | 5.1 | 2.1 | 4.2 | 11.1 | 4.3 | -6.6 | -4.3 | -3.1 | 5.9 | 8.7 | 5.5 | 14.2 | 15.1 | 3.8 | 11.6 | -1.2 | 0.8 | 2.8 | -1.9 | -1.1 | **2.4** | **-3.0** | **5.6** | **-5.3** |
| EAC(30-25°S) | -15.9 | -13.5 | -22.5 | -22.0 | -23.2 | -23.2 | -26.2 | -24.6 | -23.0 | -20.4 | -24.0 | -30.2 | -25.8 | -17.1 | -19.8 | -19.8 | -29.0 | -28.9 | -21.0 | -26.2 | -26.9 | -26.5 | -62.4 | -20.2 | -16.8 | -17.7 | -15.0 | -15.6 | **-22.7** | **-26.2** | **-19.3** | **-11.7** |
| EACx(40-35°S) | -2.5 | -6.0 | -9.9 | -9.8 | -7.5 | -5.5 | -0.7 | 0.1 | -3.4 | -1.9 | -2.4 | -1.4 | -0.5 | -9.5 | 0.1 | -8.4 | 4.4 | 1.1 | 10.1 | 6.4 | 7.3 | 4.2 | 0.7 | -3.9 | -0.3 | -0.1 | -3.1 | -2.5 | **-1.7** | **-4.3** | **0.2** | **-5.5** |
| MC(7-10°N) | -23.0 | -28.2 | -28.7 | -27.3 | -32.2 | -31.7 | -24.4 | -24.7 | -24.8 | -35.8 | -18.0 | -25.6 | -21.7 | -18.1 | -26.4 | -22.2 | -22.5 | -19.6 | -22.3 | -27.3 | -26.2 | -9.0 | -3.5 | -17.8 | -22.1 | -21.7 | -29.9 | -30.1 | **-24.5** | **-27.6** | **-21.7** | **-17.9** |
| KC(20-25°N) | 31.2 | 27.1 | 27.7 | 28.5 | 31.5 | 27.7 | 21.6 | 22.7 | 22.5 | 32.9 | 29.6 | 37.5 | 29.2 | 27.1 | 30.5 | 21.7 | 25.3 | 23.2 | 34.5 | 46.9 | 46.3 | 35.6 | 41.2 | 29.7 | 24.9 | 22.0 | 27.0 | 27.5 | **28.1** | **25.2** | **31.9** | **22.0** |
| KC(25-30°N) | 40.0 | 39.1 | 42.8 | 43.8 | 47.7 | 47.2 | 32.9 | 32.7 | 31.6 | 42.4 | 39.7 | 50.9 | 39.1 | 55.7 | 40.9 | 30.0 | 33.4 | 29.6 | 36.2 | 50.1 | 47.0 | 39.2 | 49.9 | 50.2 | 33.6 | 37.6 | 42.5 | 43.6 | **40.4** | **35.5** | **47.0** | **38.8** |
| KC(35-40°S) | 15.5 | 19.8 | 17.0 | 15.5 | 15.0 | 16.4 | 13.8 | 11.4 | 12.0 | 12.1 | 15.9 | 10.1 | 11.8 | 15.2 | 15.1 | 13.2 | 4.9 | 9.5 | -7.8 | 18.7 | 18.3 | 5.1 | 19.9 | 10.6 | 5.2 | 5.0 | 7.7 | 6.5 | **12.7** | **9.1** | **15.6** | **8.1** |
| OC(50-55°N) | -9.2 | -9.1 | -7.4 | -7.3 | -3.6 | -11.5 | -5.8 | -6.3 | -6.4 | -14.8 | -14.6 | -17.1 | -7.3 | -5.7 | -6.3 | -8.8 | -9.3 | -9.5 | -11.8 | -15.1 | -15.1 | -5.4 | -7.6 | -8.2 | -13.4 | -13.8 | -6.5 | -6.7 | **-8.5** | **-12.2** | **-6.5** | **-12.8** |
| GPC(15-13°S) | 11.4 | 18.0 | 18.6 | 18.6 | 22.9 | 20.8 | 15.7 | 8.6 | 10.7 | 18.6 | 22.6 | 32.0 | 20.9 | 18.2 | 21.0 | 25.5 | 12.7 | 14.0 | 5.8 | 11.3 | 10.8 | 9.4 | 18.9 | 20.6 | 20.8 | 20.8 | 13.7 | 15.2 | **18.4** | **12.4** | **20.8** | **12.2** |
| NZ(38-34°S) | -16.5 | -15.5 | -11.5 | -11.7 | -11.6 | -13.4 | -26.2 | -21.7 | -18.6 | -16.7 | -25.3 | -26.7 | -20.7 | -6.5 | -17.9 | -12.9 | -18.6 | -18.7 | -10.5 | -29.8 | -29.8 | -18.9 | -15.1 | -19.9 | -11.2 | -11.5 | -10.3 | -9.1 | **-16.6** | **-20.1** | **-11.6** | **-4.4** |
| NZ(50-46°S) | 9.0 | 7.9 | 11.3 | 13.6 | 7.3 | 11.3 | 11.1 | 10.4 | 10.9 | 16.2 | 21.4 | 24.5 | 5.3 | 11.8 | 9.8 | 13.6 | 10.2 | 12.1 | 2.6 | 27.9 | 25.8 | 9.6 | 4.4 | 13.2 | 13.7 | 13.3 | 15.2 | 13.7 | **11.6** | **9.7** | **13.7** | **16.2** |
| NGC(10-5°S) | 25.3 | 25.7 | 21.0 | 20.0 | 15.5 | 14.3 | 14.6 | 21.4 | 25.3 | 26.2 | 24.1 | 39.3 | 22.9 | 17.3 | 20.7 | 22.2 | 22.6 | 21.5 | 17.1 | 36.7 | 36.6 | 19.5 | 31.9 | 20.7 | 14.9 | 14.2 | 24.1 | 23.3 | **21.9** | **19.0** | **25.3** | **11.8** |
| BC(40-35°S) | -18.7 | -18.6 | -26.0 | -24.1 | -29.2 | -29.1 | -10.2 | -10.2 | -10.8 | -16.2 | -13.6 | -23.6 | -16.4 | 0.0 | 4.5 | -8.7 | -8.7 | -10.1 | -8.0 | -16.0 | -15.5 | -15.8 | -28.5 | -23.1 | -7.2 | -6.6 | -6.5 | -9.5 | **-14.5** | **-19.8** | **-8.7** | **-18.5** |
| NBC(10-5°S) | 18.0 | 20.3 | 25.7 | 26.5 | 26.0 | 24.4 | 21.6 | 21.3 | 20.9 | 16.5 | 19.5 | 19.3 | 28.7 | 29.6 | 28.9 | 20.1 | 18.2 | 20.8 | 8.5 | 21.3 | 21.4 | 24.8 | 23.9 | 27.2 | 17.4 | 17.5 | 32.3 | 31.9 | **21.3** | **19.5** | **26.1** | **15.2** |
| GS(28-33°N) | 45.8 | 44.2 | 42.3 | 43.0 | 38.7 | 41.2 | 39.5 | 36.3 | 35.8 | 31.6 | 42.7 | 31.5 | 46.7 | 46.5 | 47.9 | 34.8 | 32.0 | 37.6 | 19.1 | 40.3 | 40.4 | 53.2 | 62.5 | 44.7 | 31.3 | 31.0 | 44.8 | 44.9 | **40.8** | **35.5** | **44.8** | **43.3** |
| LAB(52-5°N) | -6.0 | -4.8 | -12.1 | -16.9 | -14.4 | -15.7 | -7.3 | -5.7 | -4.7 | -9.2 | -8.4 | -14.8 | -9.9 | -6.4 | -9.1 | -17.2 | -6.3 | -8.3 | -5.5 | -12.7 | -12.3 | -10.9 | | -13.6 | -8.2 | -7.8 | -29.1 | -26.7 | **-9.2** | **-14.0** | **-6.8** | **-6.3** |
| AC(34-30°S) | -58.6 | -59.5 | -53.2 | -53.5 | -50.6 | -53.5 | -59.1 | -60.4 | -59.0 | -50.3 | -72.3 | -55.7 | -65.1 | -76.4 | -67.2 | -59.0 | -50.0 | -55.5 | -43.4 | -68.5 | -70.0 | -66.0 | -42.0 | -69.0 | -51.7 | -51.6 | -66.7 | -66.0 | **-59.0** | **-66.2** | **-52.9** | **-51.6** |
| AC(20-15°S) | -20.6 | -19.3 | -17.8 | -17.4 | -17.0 | -18.5 | -15.8 | -19.8 | -17.9 | -22.0 | -23.5 | -21.4 | -19.3 | -27.5 | -23.3 | -14.7 | -26.2 | -25.1 | -19.3 | -17.7 | -17.8 | -21.4 | -18.4 | -18.3 | -13.7 | -13.6 | -30.7 | -31.4 | **-19.3** | **-22.3** | **-17.8** | **-16.4** |
| EACC(10-5°S) | 19.5 | 17.8 | 18.8 | 18.4 | 22.3 | 21.7 | 12.1 | 9.4 | 9.3 | 18.8 | 20.1 | 25.3 | 17.0 | 19.3 | 19.7 | 17.0 | 12.5 | 12.2 | 8.9 | 20.5 | 20.7 | 17.0 | 22.9 | 22.6 | 11.4 | 11.0 | 16.8 | 16.1 | **18.1** | **12.4** | **20.2** | **13.6** |
| MNC(14-11°S) | 19.8 | 19.1 | 21.9 | 21.6 | 25.7 | 23.9 | 28.1 | 23.5 | 22.4 | 17.0 | 18.0 | 28.6 | 25.0 | 25.3 | 25.2 | 15.5 | 30.4 | 29.5 | 22.1 | 20.2 | 17.7 | 25.6 | 27.8 | 24.9 | 16.2 | 15.3 | 24.3 | 23.9 | **23.7** | **19.6** | **25.4** | **19.1** |
| SMC(28-23°S) | -13.8 | -14.0 | -16.6 | -17.0 | -19.5 | -19.1 | -22.6 | -19.5 | -19.3 | -9.4 | -16.9 | -14.1 | -14.5 | -14.3 | -14.3 | -15.2 | -15.7 | -15.8 | -11.8 | -23.8 | -25.1 | -18.4 | -13.2 | -25.2 | -15.9 | -14.0 | -11.5 | -13.2 | **-15.7** | **-19.1** | **-14.0** | **-13.2** |
| ATL18(18-22°N) | 15.6 | 12.8 | 19.5 | 26.1 | 26.3 | 27.0 | 7.0 | 16.9 | 6.6 | -2.2 | 14.5 | 29.1 | 30.7 | 16.0 | 31.2 | 20.6 | 14.0 | 20.8 | 18.1 | 14.3 | 14.0 | 15.7 | 9.3 | 18.0 | 21.3 | 20.8 | 14.8 | 14.6 | **16.5** | **14.2** | **21.0** | **18.6** |
| ATL25(25-30°N) | 42.0 | 40.5 | 39.6 | 39.7 | 37.8 | 37.9 | 27.7 | 29.1 | 28.2 | 33.8 | 37.8 | 31.5 | 41.2 | 43.9 | 39.9 | 34.0 | 25.0 | 37.2 | 16.5 | 38.6 | 38.6 | 42.2 | 43.8 | 41.0 | 28.9 | 28.5 | 47.3 | 47.1 | **38.3** | **30.9** | **41.1** | **42.3** |

*Table S2 0-1000m integrated projected transport change (RCP8.5) for currents described in the text (CMIP5 models). Median change is significant at 95% level using a two-sided Wilcoxon signed rank test unless in parenthesis*

| | ACCESS1-0 | ACCESS1-3 | CCSM4 | CESM1-BGC | CESM1-CAM5-1-FV2 | CESM1-CAM5 | CMCC-CESM | CMCC-CMS | CMCC-CM | CNRM-CM5 | CanESM2 | FIO-ESM | GFDL-CM3 | GFDL-ESM2G | GFDL-ESM2M | HadGEM2-AO | IPSL-CM5A-LR | IPSL-CM5A-MR | IPSL-CM5B-LR | MIROC-ESM-CHEM | MIROC-ESM | MIROC5 | MPI-ESM-LR | MPI-ESM-MR | MRI-CGCM3 | MRI-ESM1 | NorESM1-ME | NorESM1-M | **Median** | **25th percentile** | **75th percentile** | |
|---|---|---|---|---|---|---|---|---|---|---|---|---|---|---|---|---|---|---|---|---|---|---|---|---|---|---|---|---|---|---|---|---|
| ITF | 3.5 | 3.2 | 2.6 | 2.6 | 2.5 | 3.2 | 1.2 | 1.4 | 1.4 | 2.0 | 3.2 | 3.5 | 4.1 | 5.0 | 4.8 | 3.2 | 1.2 | 1.9 | 1.3 | 3.4 | 3.0 | 4.2 | 4.6 | 4.3 | 3.0 | 2.7 | 3.9 | 3.4 | **3.2** | **2.4** | **3.6** | |
| ACx(25°E) | -10.3 | -9.8 | 1.3 | 3.4 | 3.5 | 3.4 | -9.2 | -11.0 | -9.7 | -5.0 | -17.5 | 1.7 | -12.5 | -7.3 | -24.5 | -10.4 | -13.4 | -15.7 | -3.5 | -15.3 | -15.8 | -17.8 | -6.3 | 3.8 | -11.1 | -11.3 | -6.4 | -7.4 | **-9.7** | **-12.7** | **-4.6** | |
| KC(140°E) | 0.0 | -0.9 | -3.2 | -2.1 | -4.5 | -2.6 | -2.4 | -4.6 | -3.2 | 0.4 | -0.2 | -0.7 | -5.4 | -0.9 | -2.6 | 2.3 | -3.0 | -4.2 | -5.3 | -3.6 | -4.7 | -3.9 | -1.9 | -0.8 | -0.8 | 0.2 | -4.9 | -6.5 | **-2.6** | **-4.3** | **-0.8** | |
| KC(150°E) | -0.9 | -1.6 | -2.9 | -1.7 | -4.9 | -2.2 | 0.1 | -1.4 | -2.9 | 1.2 | 1.1 | 1.8 | -3.1 | -0.8 | -2.4 | 2.2 | -3.2 | -3.1 | -5.1 | -2.3 | -2.5 | -5.2 | -1.6 | 1.5 | 0.1 | 1.6 | -5.3 | -6.4 | **-2.0** | **-3.1** | **0.1** | |
| GS(300°E) | -13.9 | -13.1 | -10.2 | -11.3 | -10.7 | -10.7 | -7.0 | -6.9 | -5.8 | -5.8 | -9.7 | -10.1 | -13.4 | -11.0 | -13.0 | -7.5 | -5.8 | -9.4 | -2.1 | -9.4 | -9.1 | -15.7 | -10.8 | -8.2 | -5.1 | -4.9 | -9.0 | -8.2 | **-9.4** | **-10.8** | **-7.0** | |
| TASL(146°E) | -9.0 | -4.7 | -3.6 | -3.6 | -6.0 | -4.4 | -3.3 | -7.4 | -6.3 | -3.3 | -8.9 | -8.9 | -8.8 | -12.7 | -7.1 | -3.8 | -4.5 | -8.7 | -0.7 | -12.7 | -12.9 | -10.9 | -15.7 | -5.3 | -4.1 | -4.3 | -3.5 | -3.8 | **-5.7** | **-8.9** | **-3.8** | |
| EAC(30-25°S) | -0.7 | -3.3 | 2.7 | 2.4 | 1.6 | 0.6 | -1.9 | -1.0 | 0.2 | 0.9 | 1.6 | 2.1 | 0.5 | 2.1 | 1.0 | -2.0 | 3.4 | 5.0 | -4.2 | 0.0 | 0.0 | 2.5 | 3.4 | -0.5 | -2.1 | -1.9 | 0.3 | 1.9 | **(0.5)** | **-0.8** | **2.1** | |
| EACx(40-35°S) | -7.8 | -6.0 | -2.8 | -2.9 | -6.5 | -4.6 | -5.1 | -9.1 | -8.6 | -2.7 | -8.5 | -4.8 | -6.3 | -4.2 | -7.2 | -5.0 | -7.5 | -7.9 | -3.1 | -10.1 | -10.5 | -9.3 | -16.1 | -5.2 | -4.7 | -3.9 | -2.0 | -3.5 | **-5.6** | **-8.0** | **-4.2** | |
| MC(7-10°N) | 1.8 | 4.1 | 3.3 | 3.2 | 2.2 | 4.3 | 0.1 | 3.8 | 5.2 | 4.9 | 5.5 | -1.9 | 6.6 | 4.4 | 5.9 | 7.3 | 6.6 | 5.6 | 2.6 | 3.0 | 3.8 | -0.2 | 1.2 | 3.4 | 2.6 | 2.0 | -0.7 | -0.3 | **3.3** | **2.0** | **5.0** | |
| KC(20-25°N) | -3.1 | -1.5 | -0.5 | -1.5 | -1.9 | -1.9 | -2.4 | -3.4 | -4.3 | -2.2 | -2.5 | -0.9 | -5.8 | -4.6 | -4.1 | -2.7 | -3.9 | -3.0 | -4.6 | -2.9 | -3.7 | -3.6 | -2.7 | -2.5 | -0.5 | -0.7 | -1.5 | -2.1 | **-2.6** | **-3.6** | **-1.8** | |
| KC(25-30°N) | -0.3 | 0.6 | -4.1 | -2.7 | -4.5 | -3.0 | -0.9 | -1.0 | -2.3 | 0.4 | 0.8 | 0.0 | -1.1 | -4.9 | -1.9 | 0.6 | -1.9 | -1.2 | -1.5 | -1.4 | -1.4 | -2.5 | -1.2 | -2.2 | 1.2 | 0.7 | -2.9 | -4.5 | **-1.4** | **-2.5** | **-0.2** | |
| KC(35-40°S) | 2.2 | -1.4 | -0.6 | -0.4 | 1.6 | -1.3 | 4.3 | 6.7 | 6.5 | -0.6 | -1.5 | -0.1 | 2.2 | 2.3 | 1.9 | -0.8 | 2.9 | 5.7 | 3.6 | 2.3 | 3.6 | 2.5 | 4.3 | 2.5 | 0.3 | -0.1 | -0.7 | 0.1 | **2.0** | **-0.5** | **3.1** | |
| OC(50-55°N) | -0.8 | -1.0 | -1.0 | -0.9 | 0.1 | 0.1 | 0.0 | -0.4 | -0.2 | -1.3 | -3.0 | -3.3 | -0.4 | -1.9 | -1.0 | -1.4 | -1.1 | -0.8 | -0.5 | -1.5 | -2.0 | 0.0 | -2.4 | -2.8 | -0.2 | -0.4 | 0.6 | 0.2 | **-0.9** | **-1.4** | **-0.2** | |
| GPC(15-13°S) | 3.5 | 3.5 | -0.4 | 1.0 | 0.2 | 0.7 | 4.0 | 6.0 | 4.5 | 2.3 | -0.4 | 2.1 | 3.5 | 3.6 | 2.5 | 1.7 | 6.1 | 2.4 | 5.8 | 2.4 | 1.7 | 1.9 | 13.9 | 5.3 | 1.0 | 1.1 | -0.2 | 0.5 | **2.3** | **1.0** | **3.7** | |
| NZ(38-34°S) | 3.8 | 4.5 | 5.3 | 5.3 | 4.4 | 3.7 | -1.6 | 1.4 | 0.2 | 0.1 | 5.9 | 5.8 | 3.1 | 2.6 | 3.0 | 2.1 | 1.8 | 2.5 | -2.9 | 1.2 | 1.2 | 5.3 | 6.0 | 5.0 | 0.2 | 0.9 | -0.2 | 0.6 | **2.5** | **0.8** | **4.6** | |
| NZ(50-46°S) | -0.6 | -2.0 | -4.9 | -5.9 | -3.7 | -1.6 | 3.3 | 1.4 | 1.3 | 0.9 | -1.7 | -4.6 | 0.6 | 1.6 | 1.2 | 0.3 | 1.6 | 0.5 | 3.5 | 0.4 | 1.8 | -1.5 | -1.8 | -2.5 | 0.5 | 0.0 | -0.7 | -1.2 | **0.1** | **-1.7** | **1.2** | |
| NGC(10-5°S) | 1.8 | 5.1 | 0.2 | 0.8 | 6.3 | 4.1 | 11.6 | 6.3 | 2.4 | -1.5 | 5.4 | -0.7 | 1.8 | 3.4 | 2.8 | 2.6 | 1.3 | 0.3 | 4.8 | 5.6 | 6.4 | 8.6 | 7.8 | 0.7 | 5.4 | 4.6 | -0.2 | 0.0 | **3.1** | **0.8** | **5.5** | |
| BC(40-35°S) | -8.6 | -6.5 | -9.0 | -9.9 | -4.7 | -6.7 | -0.8 | -3.6 | -2.9 | -1.6 | -4.2 | -7.5 | -9.2 | -8.1 | -11.0 | -9.0 | -2.2 | -2.7 | -1.0 | -8.2 | -8.3 | -7.5 | -6.4 | -13.4 | -1.9 | -2.1 | 0.8 | 6.3 | **-6.4** | **-8.4** | **-2.2** | |
| NBC(10-5°S) | -3.9 | -4.1 | -5.1 | -5.4 | -6.1 | -5.5 | -0.4 | -1.0 | -1.0 | -3.5 | -2.8 | -5.6 | -7.0 | -7.2 | -5.9 | -1.2 | -1.7 | -3.4 | 0.4 | -1.7 | -1.2 | -5.3 | -2.5 | -3.0 | -1.5 | -1.6 | -5.3 | -5.0 | **-3.4** | **-5.4** | **-1.6** | |
| GS(28-33°N) | -15.2 | -13.5 | -8.5 | -8.8 | -8.8 | -10.0 | -4.9 | -5.2 | -4.2 | -3.6 | -11.0 | -10.7 | -13.1 | -12.0 | -12.6 | -6.5 | -4.6 | -7.4 | -0.8 | -7.6 | -7.4 | -15.2 | -14.8 | -8.8 | -3.8 | -2.8 | -8.4 | -8.2 | **-8.5** | **-11.2** | **-5.2** | |
| LAB(52-5°N) | 2.0 | 1.0 | 5.5 | 8.9 | 9.0 | 10.9 | 0.0 | -1.1 | -1.3 | 0.1 | 0.9 | 5.4 | 3.4 | 0.1 | 3.8 | 8.9 | -1.6 | -0.2 | 1.9 | -1.9 | -1.3 | 2.9 | 0.0 | -3.7 | 3.0 | 2.0 | 11.7 | 9.5 | **1.9** | **-0.1** | **5.5** | |
| AC(34-30°S) | 9.2 | 8.9 | 5.3 | 5.4 | 4.4 | 6.0 | -1.4 | 1.0 | 3.2 | 3.5 | 6.2 | 6.4 | 11.9 | 9.6 | 11.1 | 5.7 | -2.8 | 3.6 | -1.0 | 4.9 | 4.5 | 9.9 | 3.8 | 9.7 | 2.6 | 2.4 | 7.0 | 5.2 | **5.3** | **3.4** | **7.5** | |
| AC(20-15°S) | 5.0 | 2.8 | 3.9 | 3.9 | 4.2 | 3.3 | 2.9 | 4.8 | 4.8 | 4.5 | 4.2 | 5.1 | 7.2 | 9.5 | 7.7 | 3.0 | 6.4 | 7.4 | 1.9 | 3.5 | 3.5 | 6.5 | 4.7 | 5.3 | 2.9 | 2.5 | 7.2 | 6.2 | **4.6** | **3.4** | **6.2** | |
| EACC(10-5°S) | 0.2 | 0.3 | -1.7 | -1.3 | -1.9 | -1.7 | -0.1 | 1.9 | 1.2 | -0.8 | -4.2 | -2.5 | 0.2 | 1.8 | -0.1 | 0.3 | 0.1 | -0.3 | 0.7 | -1.2 | -1.3 | 1.2 | -0.6 | -0.2 | 0.6 | 0.9 | 0.1 | -0.5 | **(-0.1)** | **-1.2** | **0.4** | |
| MNC(14-11°S) | -3.0 | -3.5 | -3.8 | -3.5 | -3.8 | -5.3 | -2.4 | -1.3 | -2.3 | -2.0 | -4.4 | -5.2 | -4.3 | -2.7 | -3.4 | -2.1 | -3.1 | -5.0 | -0.1 | -1.6 | -1.4 | -4.5 | -5.1 | -3.2 | -2.1 | -1.3 | -2.3 | -2.7 | **-3.1** | **-3.9** | **-2.1** | |
| SMC(28-23°S) | 1.0 | 2.7 | 2.0 | 2.4 | 1.9 | 3.3 | 1.3 | 0.3 | 1.0 | 0.1 | 2.6 | 2.5 | 0.5 | -0.1 | 1.3 | 1.5 | 1.2 | 1.7 | -0.2 | 3.5 | 3.8 | 2.5 | 1.2 | 3.0 | 2.1 | 1.7 | 0.6 | 1.3 | **1.6** | **1.0** | **2.5** | |
| ATL18(18-22°N) | -4.4 | -2.8 | -2.4 | -3.0 | -4.5 | -4.5 | 0.1 | -0.4 | 0.3 | -1.4 | 0.1 | -6.5 | -9.7 | -3.7 | -8.8 | -2.5 | -1.1 | -2.3 | -1.2 | -2.5 | -2.3 | -2.6 | 0.3 | -1.9 | -1.3 | -1.0 | -2.0 | -1.9 | **-2.3** | **-3.1** | **-1.1** | |
| ATL25(25-30°N) | -13.9 | -11.6 | -8.1 | -7.8 | -8.5 | -8.8 | -2.7 | -3.8 | -2.8 | -5.9 | -10.5 | -10.6 | -12.9 | -13.2 | -10.8 | -6.8 | -4.8 | -7.7 | -1.7 | -9.3 | -8.9 | -12.0 | -9.1 | -9.1 | -3.2 | -2.2 | -9.5 | -9.1 | **-8.8** | **-10.6** | **-5.6** | |

*Table S3 0-1000m integrated historical transport for currents described in the text (CMIP6 models)*

| **Historical** | ACCESS-CM2 | ACCESS-ESM1-5 | BCC-CSM2-MR | CAMS-CSM1-0 | CESM2-WACCM | CESM2 | CIESM | CMCC-CM2-SR5 | CNRM-CM6-1 | CNRM-ESM2-1 | CanESM5 | EC-Earth3-Veg | GISS-E2-1-G | INM-CM4-8 | INM-CM5-0 | IPSL-CM6A-LR | MIROC-ES2L | MIROC6 | MPI-ESM1-2-HR | MPI-ESM1-2-LR | MRI-ESM2-0 | NESM3 | NorESM2-LM | NorESM2-MM | UKESM1-0-LL | **Median** | **25th percentile** | **75th percentile** | **Reanalysis mean** |
|---|---|---|---|---|---|---|---|---|---|---|---|---|---|---|---|---|---|---|---|---|---|---|---|---|---|---|---|---|---|
| ITF | -14.5 | -13.9 | -9.8 | -11.6 | -13.4 | -13.1 | -10.9 | -12.9 | -13.1 | -12.9 | -11.1 | -13.6 | -18.4 | -2.1 | -4.3 | -13.5 | -12.0 | -13.1 | -11.6 | -12.8 | -14.4 | -10.4 | -18.6 | -19.5 | -14.3 | **-13.1** | **-13.9** | **-11.6** | **-14.7** |
| ACx(25°E) | -54.0 | -59.3 | -18.9 | -24.5 | -62.3 | -58.9 | -51.4 | -50.7 | -39.7 | -37.4 | -57.1 | -65.5 | -18.9 | -27.5 | -11.6 | -50.3 | -22.5 | -34.4 | -75.4 | -64.2 | -51.8 | -24.5 | -36.4 | -36.6 | -53.9 | **-50.3** | **-57.1** | **-27.5** | **-43.1** |
| KC(140°E) | 48.2 | 42.2 | 52.6 | 51.2 | 42.7 | 42.2 | 40.7 | 41.0 | 36.8 | 37.7 | 43.5 | 49.2 | 38.7 | 12.0 | 14.3 | 40.6 | 43.2 | 48.4 | 44.3 | 54.9 | 49.2 | 37.9 | 40.9 | 44.2 | 39.7 | **42.2** | **39.7** | **48.2** | **36.0** |
| KC(150°E) | 46.9 | 41.2 | 47.5 | 47.4 | 42.0 | 40.7 | 41.1 | 42.4 | 38.7 | 41.5 | 45.4 | 51.3 | 37.5 | 52.3 | 49.9 | 44.9 | 44.3 | 47.1 | 50.7 | 52.1 | 54.6 | 38.0 | 43.8 | 45.8 | 41.6 | **44.9** | **41.5** | **47.5** | **44.3** |
| GS(300°E) | 34.8 | 35.9 | 45.0 | 33.4 | 34.8 | 33.9 | 37.8 | 34.6 | 34.2 | 34.5 | 32.3 | 31.9 | 44.9 | 30.3 | 23.5 | 29.2 | 36.8 | 36.4 | 40.1 | 43.2 | 40.0 | 28.4 | 39.2 | 42.1 | 31.2 | **34.8** | **32.3** | **39.2** | **40.2** |
| TASL(146°E) | -2.9 | -6.7 | -2.7 | -1.5 | -2.7 | -2.7 | -3.6 | -8.9 | -0.9 | -2.0 | -0.8 | -4.0 | 0.2 | -5.2 | -3.4 | 4.0 | 7.1 | -1.5 | -5.4 | 5.3 | 10.1 | -2.3 | -7.5 | -5.6 | -1.1 | **-2.7** | **-4.0** | **-0.9** | **-5.3** |
| EAC(30-25°S) | -17.5 | -14.3 | -20.9 | -14.6 | -20.8 | -21.3 | -19.0 | -14.9 | -23.3 | -23.7 | -21.1 | -18.8 | -20.8 | -19.9 | -22.3 | -25.9 | -30.8 | -21.4 | -19.5 | -18.4 | -27.0 | -12.9 | -14.9 | -13.8 | -22.1 | **-20.8** | **-22.1** | **-17.5** | **-11.7** |
| EACx(40-35°S) | -4.6 | -7.9 | -13.6 | -5.3 | -6.1 | -5.7 | -4.4 | -11.2 | -2.6 | -2.5 | -7.0 | -3.0 | 0.3 | -15.2 | 1.2 | 1.0 | 6.5 | 1.7 | -7.5 | -1.4 | 2.5 | -5.4 | -7.0 | -4.3 | -5.3 | **-4.6** | **-7.0** | **-1.4** | **-5.5** |
| MC(7-10°N) | -18.0 | -27.4 | -32.3 | -17.7 | -28.5 | -26.9 | -27.9 | -25.1 | -25.7 | -25.5 | -23.8 | -20.1 | -9.3 | -0.8 | -2.0 | -24.7 | -26.1 | -24.0 | -18.1 | -4.5 | -20.0 | -10.6 | -20.9 | -23.5 | -22.6 | **-23.5** | **-25.7** | **-18.0** | **-17.9** |
| KC(20-25°N) | 31.0 | 25.9 | 35.2 | 26.9 | 23.8 | 25.1 | 27.8 | 17.2 | 20.8 | 33.0 | 22.6 | 33.8 | 18.5 | 2.8 | 8.7 | 23.4 | 43.3 | 37.6 | 28.2 | 32.4 | 24.5 | 16.5 | 18.1 | 15.5 | 28.0 | **25.1** | **18.5** | **31.0** | **22.0** |
| KC(25-30°N) | 39.4 | 35.0 | 32.7 | 26.2 | 26.2 | 36.7 | 42.4 | 36.4 | 40.0 | 31.4 | 34.6 | 52.6 | 29.7 | 1.2 | 3.5 | 45.6 | 43.7 | 49.6 | 38.7 | 30.2 | 47.5 | 26.2 | 42.7 | 44.7 | 37.3 | **36.7** | **30.2** | **42.7** | **38.8** |
| KC(35-40°S) | 14.1 | 20.9 | 21.2 | 23.1 | 18.5 | 18.2 | 11.8 | 12.5 | 5.2 | 4.7 | 15.1 | 12.6 | 15.6 | 4.1 | 4.0 | 7.1 | 1.4 | 5.6 | 7.8 | 18.4 | 7.3 | 15.6 | 10.4 | 9.7 | 13.9 | **12.5** | **7.1** | **15.6** | **8.1** |
| OC(50-55°N) | -10.2 | -7.9 | -6.4 | -9.4 | -9.4 | -9.5 | -7.5 | -11.8 | -12.3 | -12.1 | -11.0 | -15.2 | -6.5 | 0.1 | -7.2 | -15.0 | -10.0 | -11.7 | -10.2 | -11.9 | -12.5 | -5.3 | -10.8 | -11.0 | -11.4 | **-10.2** | **-11.8** | **-7.9** | **-12.8** |
| GPC(15-13°S) | 16.4 | 17.0 | 4.0 | 17.9 | 13.6 | 14.4 | 10.5 | 21.9 | 23.6 | 23.4 | 17.2 | 20.0 | 20.9 | 24.4 | 17.5 | 16.9 | 5.7 | 4.7 | 24.8 | 7.3 | 24.3 | 10.1 | 22.6 | 14.2 | 23.4 | **17.2** | **13.6** | **22.6** | **12.2** |
| NZ(38-34°S) | -19.4 | -16.9 | -22.9 | -16.1 | -12.1 | -11.9 | -11.9 | -12.8 | -14.5 | -13.8 | -11.6 | -8.8 | -17.0 | -6.6 | -13.5 | -14.1 | -23.9 | -11.2 | -15.6 | -19.6 | -18.9 | -9.9 | -7.3 | -7.8 | -17.0 | **-13.8** | **-17.0** | **-11.6** | **-4.4** |
| NZ(50-46°S) | 9.8 | 9.1 | 7.8 | 6.2 | 12.0 | 7.1 | 10.0 | 14.6 | 17.0 | 15.7 | 12.5 | 12.2 | 11.7 | -5.5 | 28.0 | 13.7 | 13.5 | 13.5 | 12.7 |  | 12.0 | 9.6 | 7.3 | 7.5 | 15.2 | **12.0** | **8.8** | **13.6** | **16.2** |
| NGC(10-5°S) | 24.9 | 21.5 | 19.9 | 16.6 | 18.5 | 19.2 | 17.6 | 17.4 | 29.3 | 27.2 | 19.9 | 16.7 | 16.0 | 19.5 | 14.7 | 28.0 | 25.5 | 17.9 | 24.6 | 28.0 | 26.8 | 14.2 | 18.1 | 16.3 | 28.0 | **19.5** | **17.4** | **25.5** | **11.8** |
| BC(40-35°S) | -20.9 | -21.9 | -12.8 | -21.9 | -29.3 | -28.7 | -25.2 | -15.0 | -14.5 | -13.5 | -30.4 | -25.8 | -4.7 | -30.5 | -3.6 | -20.6 | -6.4 | -12.4 | -27.3 | -45.1 | -15.6 | -13.9 | -18.0 | -18.6 | -15.3 | **-18.6** | **-25.8** | **-13.9** | **-18.5** |
| NBC(10-5°S) | 22.3 | 20.9 | 29.1 | 19.2 | 22.5 | 22.8 | 20.2 | 23.8 | 21.5 | 21.8 | 17.8 | 18.4 | 29.1 | 18.2 | 27.2 | 17.7 | 21.1 | 21.4 | 26.2 | 25.9 | 23.2 | 12.4 | 23.3 | 23.1 | 20.3 | **21.8** | **20.2** | **23.3** | **15.2** |
| GS(28-33°N) | 44.2 | 41.4 | 53.7 | 38.1 | 38.2 | 29.7 | 38.0 | 36.3 | 39.6 | 40.2 | 36.2 | 39.8 | 46.0 | 31.3 | 31.1 | 39.9 | 43.8 | 45.6 | 42.2 | 52.5 | 42.3 | 33.3 | 41.1 | 42.6 | 39.8 | **39.9** | **38.0** | **42.6** | **43.3** |
| LAB(52-5°N) | -2.8 | -4.0 | 6.7 | -1.0 | -11.8 | -15.6 | -7.1 | -7.1 | -8.4 | -8.1 | -6.1 | -8.3 | -11.5 | -13.7 | -7.2 | -7.2 | -11.0 | -9.9 | -15.4 |  | -5.5 | -8.3 | -15.2 | -23.6 | -9.3 | **-8.3** | **-11.6** | **-6.8** | **-6.3** |
| AC(34-30°S) | -60.4 | -60.3 | -65.5 | -58.2 | -50.6 | -50.8 | -45.0 | -57.0 | -61.4 | -61.1 | -57.1 | -63.2 | -64.8 | -35.5 | -14.1 | -63.0 | -55.6 | -58.1 | -61.5 | -38.2 | -59.3 | -49.2 | -61.6 | -66.0 | -67.6 | **-59.3** | **-61.6** | **-50.8** | **-51.6** |
| AC(20-15°S) | -18.5 | -17.0 | -16.6 | -16.7 | -22.3 | -23.2 | -23.6 | -25.2 | -24.7 | -24.8 | -16.0 | -20.7 | -28.3 | -18.5 | -16.0 | -24.5 | -20.1 | -21.9 | -13.8 | -20.5 | -24.9 | -13.0 | -23.0 | -24.2 | -26.9 | **-21.9** | **-24.5** | **-17.0** | **-16.4** |
| EACC(10-5°S) | 18.2 | 17.3 | 14.6 | 13.8 | 20.3 | 19.4 | 20.6 | 23.6 | 17.9 | 17.4 | 19.9 | 16.6 | 12.1 | 19.6 | 17.0 | 21.5 | 17.9 | 16.5 | 18.9 | 22.1 | 18.4 | 14.0 | 15.2 | 16.5 | 21.2 | **17.9** | **16.5** | **19.9** | **13.6** |
| MNC(14-11°S) | 20.0 | 19.4 | 20.2 | 20.2 | 22.4 | 21.3 | 21.1 | 25.6 | 19.4 | 19.2 | 23.4 | 17.0 | 18.5 | 11.2 | 16.5 | 22.7 | 27.6 | 25.6 | 21.3 | 29.2 | 20.7 | 8.3 | 19.9 | 21.7 | 25.2 | **20.7** | **19.4** | **22.7** | **19.1** |
| SMC(28-23°S) | -16.3 | -16.5 | -13.9 | -16.3 | -10.7 | -9.8 | -8.9 | -12.3 | -8.2 | -6.0 | -14.1 | -14.5 | -10.2 | -1.1 | -10.2 | -10.5 | -18.2 | -15.2 | -23.1 | -19.5 | -13.7 | -15.7 | -13.4 | -15.6 | -6.6 | **-13.7** | **-15.7** | **-10.2** | **-13.2** |
| YC(18-22°N) | 7.1 | 13.6 | 17.8 | 17.0 | 17.8 | 21.3 | 17.1 | 23.3 | 34.4 | 30.8 | 16.8 | 37.4 | 8.0 | 24.3 | 18.5 | 36.1 | 7.6 | 8.6 | 17.7 | 8.4 | 18.5 | 12.8 | 16.9 | 15.1 | 25.3 | **17.7** | **13.6** | **23.3** | **18.6** |
| ATL25(25-30°N) | 43.4 | 39.5 | 49.6 | 37.2 | 35.7 | 24.4 | 36.4 | 28.5 | 36.5 | 35.7 | 31.4 | 16.5 | 48.6 | 34.0 | 31.9 | 35.1 | 43.1 | 40.0 | 40.1 | 35.0 | 39.9 | 27.2 | 38.8 | 42.1 | 30.0 | **36.4** | **31.9** | **40.0** | **42.3** |

*Table S4 0-1000m integrated projected transport change (ssp126) for currents described in the text (CMIP6 models) . Median change is significant at 95% level using a two-sided Wilcoxon signed rank test unless in parenthesis*

| SSP126 change | ACCESS-CM2 | ACCESS-ESM1-5 | BCC-CSM2-MR | CAMS-CSM1-0 | CESM2-WACCM | CESM2 | CIESM | CMCC-CM2-SR5 | CNRM-CM6-1 | CNRM-ESM2-1 | CanESM5 | EC-Earth3-Veg | GISS-E2-1-G | INM-CM4-8 | INM-CM5-0 | IPSL-CM6A-LR | MIROC-ES2L | MIROC6 | MPI-ESM1-2-HR | MPI-ESM1-2-LR | MRI-ESM2-0 | NESM3 | NorESM2-LM | NorESM2-MM | UKESM1-0-LL | **Median** | **25th percentile** | **75th percentile** |
|---|---|---|---|---|---|---|---|---|---|---|---|---|---|---|---|---|---|---|---|---|---|---|---|---|---|---|---|---|
| ITF | 1.6 | 1.6 | 0.6 | 0.6 | 2.7 | 2.5 | 2.6 | 2.5 | 2.3 | 2.2 | 1.4 | 2.0 | 3.0 | 1.5 | 0.3 | 2.7 | 2.5 | 1.8 | 2.4 | 2.3 | 3.8 | 1.2 | 3.4 | 3.8 | 2.6 | **2.3** | **1.6** | **2.6** |
| ACx(25°E) | -4.7 | -2.8 | -2.7 | -2.3 | 4.0 | 2.8 | 3.3 | -8.4 | -4.5 | -5.3 | -8.2 | -2.7 | -6.7 | -4.8 | -0.9 | -4.7 | -8.1 | -7.5 | -1.6 | -4.5 | 0.1 | -4.1 | -6.1 | -6.7 | -5.7 | **-4.5** | **-6.1** | **-2.3** |
| KC(140°E) | -0.8 | 0.7 | 0.3 | -0.2 | -0.6 | -1.5 | 1.1 | -0.2 | -0.5 | -0.6 | -0.7 | -1.1 | -2.6 | 2.7 | 0.5 | -0.3 | 0.2 | -0.2 | -1.5 | 1.3 | -0.4 | -0.8 | -2.9 | -2.3 | -0.5 | **(-0.5)** | **-0.8** | **0.2** |
| KC(150°E) | -0.9 | -0.5 | -0.3 | -0.1 | -0.1 | -1.1 | 2.2 | -0.6 | 0.1 | -0.5 | -0.2 | 0.3 | -3.0 | 0.4 | -1.9 | -0.1 | 1.6 | 0.7 | 1.2 | 2.4 | 1.1 | -0.3 | -3.8 | -2.1 | 0.4 | **(-0.1)** | **-0.6** | **0.4** |
| GS(300°E) | -5.8 | -5.6 | -5.2 | -1.1 | -10.9 | -10.4 | -13.5 | -6.9 | -6.1 | -4.9 | -3.7 | -2.5 | -17.9 | -1.9 | 0.2 | -1.9 | -5.0 | -7.4 | -4.6 | -4.9 | -18.5 | -2.9 | -12.2 | -10.3 | -5.1 | **-5.2** | **-10.3** | **-3.7** |
| TASL(146°E) | -4.1 | -1.2 | -1.9 | -1.6 | -2.8 | -3.4 | -3.0 | -0.5 | -1.6 | -3.4 | -4.7 | -1.5 | -2.1 | -2.0 | -6.2 | -4.5 | -4.9 | -4.0 | -3.1 | -6.1 | -2.7 | -1.2 | -2.2 | -2.8 | -4.6 | **-2.8** | **-4.1** | **-1.9** |
| EAC(30-25°S) | 0.5 | -1.4 | 0.9 | 0.1 | -0.6 | -0.2 | -1.0 | -0.8 | -1.3 | -0.6 | 1.4 | -1.5 | -0.1 | 1.4 | 3.0 | 0.2 | 0.8 | 0.6 | 0.3 | 0.9 | 0.9 | -1.2 | 0.6 | 0.7 | -0.6 | **(0.2)** | **-0.6** | **0.8** |
| EACx(40-35°S) | -4.7 | -2.6 | -0.4 | -2.4 | -3.0 | -3.5 | -2.6 | -1.7 | -1.9 | -2.9 | -2.2 | -1.8 | -5.0 | -2.8 | -1.8 | -3.0 | -4.5 | -3.1 | -3.9 | -1.5 | -1.6 | -1.6 | -2.4 | -2.4 | -3.4 | **-2.6** | **-3.1** | **-1.8** |
| MC(7-10°N) | 1.9 | 1.7 | 1.1 | 0.2 | 3.7 | 3.2 | -1.3 | 4.9 | 1.4 | 2.1 | 2.4 | 2.8 | 1.7 | 0.0 | 0.0 | 3.4 | 2.9 | 2.5 | 1.1 | 0.4 | 0.1 | 2.3 | 2.1 | 4.0 | 3.7 | **2.1** | **1.1** | **2.9** |
| KC(20-25°N) | -1.3 | -0.6 | 0.0 | -0.8 | -1.7 | -0.9 | 1.7 | -1.0 | -1.2 | -2.0 | -0.8 | -1.5 | -1.8 | -0.3 | -0.3 | -1.4 | -1.4 | -2.6 | 0.0 | 0.3 | 0.0 | -2.5 | -2.3 | -2.2 | -2.0 | **-1.2** | **-1.8** | **-0.3** |
| KC(25-30°N) | -0.4 | 0.9 | 0.5 | 0.0 | 0.5 | 0.2 | 1.1 | 1.7 | -0.4 | -0.3 | 0.5 | -0.5 | -1.3 | -3.0 | -1.1 | -0.4 | 0.4 | -1.3 | 1.2 | 0.9 | 0.0 | 0.5 | -2.3 | -1.8 | 0.2 | **(0.0)** | **-0.5** | **0.5** |
| KC(35-40°S) | 0.3 | -2.1 | 0.6 | 1.6 | 1.4 | -0.1 | -0.9 | -0.3 | 1.3 | 1.6 | 0.7 | -1.5 | 3.2 | 1.6 | 1.2 | 0.4 | -0.4 | 1.2 | 0.3 | 0.5 | 0.1 | 0.3 | 2.8 | 2.4 | -0.6 | **0.5** | **-0.1** | **1.4** |
| OC(50-55°N) | -0.4 | -0.3 | -0.1 | -0.3 | -1.2 | -0.8 | -2.6 | -0.1 | -1.6 | -1.0 | -1.1 | -0.2 | -0.2 | -0.6 | -0.2 | 0.0 | 1.1 | -0.7 | -0.7 | -2.0 | -2.6 | -2.6 | -0.2 | -0.4 | -2.3 | **-0.6** | **-1.2** | **-0.2** |
| GPC(15-13°S) | 2.2 | 2.5 | 0.7 | 2.4 | 3.3 | 2.4 | 5.6 | -1.0 | 0.9 | 1.6 | 0.0 | 2.0 | 4.2 | -0.5 | -0.2 | 0.7 | 1.7 | 1.0 | 1.3 | 0.7 | 1.2 | 2.3 | -2.9 | 0.5 | 0.6 | **1.2** | **0.6** | **2.3** |
| NZ(38-34°S) | 3.6 | 0.0 | 1.6 | 1.1 | 1.5 | 2.0 | 2.6 | 0.8 | -1.5 | -0.7 | 1.1 | -1.5 | 3.7 | 4.4 | 6.1 | -0.1 | 1.4 | 1.7 | 3.0 | 3.9 | 2.3 | -0.1 | 1.3 | 1.1 | 1.5 | **1.5** | **0.8** | **2.6** |
| NZ(50-46°S) | -0.9 | -0.2 | 0.0 | -0.1 | -0.9 | -1.4 | -2.8 | 0.2 | 1.3 | 1.5 | -0.7 | 1.6 | -1.5 | 4.0 | 1.0 | 0.9 | 3.3 | -1.2 | -4.1 | 0.0 | -1.4 | 1.1 | -1.1 | -0.6 | 0.3 | **(-0.1)** | **-1.1** | **1.0** |
| NGC(10-5°S) | 1.7 | 1.8 | 0.8 | 2.1 | 4.0 | 3.7 | 3.0 | 0.1 | 1.1 | 1.8 | 1.4 | 2.8 | 4.2 | 1.4 | 1.6 | 0.5 | 3.4 | 3.0 | 0.9 | 1.0 | 0.3 | 3.4 | 1.6 | 1.0 | 1.6 | **1.6** | **1.0** | **3.0** |
| BC(40-35°S) | -4.0 | -2.7 | -3.6 | -1.3 | -5.9 | -4.5 | -6.5 | -5.0 | -3.3 | -2.1 | -4.8 | -2.6 | -4.9 | -7.6 | -0.9 | -2.4 | -4.2 | -3.1 | -5.2 | -4.8 | -7.4 | -2.8 | -3.9 | -4.3 | -2.0 | **-4.0** | **-4.9** | **-2.7** |
| NBC(10-5°S) | -2.1 | -1.5 | -2.9 | -0.5 | -5.2 | -5.0 | -6.8 | -3.9 | -3.0 | -2.6 | -1.4 | -0.1 | -7.5 | -1.5 | -1.4 | -1.2 | -2.3 | -1.9 | -2.2 | -1.6 | -8.9 | 1.0 | -5.4 | -4.6 | -1.5 | **-2.2** | **-4.6** | **-1.5** |
| GS(28-33°N) | -5.7 | -4.4 | -4.6 | -0.2 | -9.6 | -8.9 | -10.1 | -5.3 | -4.1 | -4.5 | -3.8 | -2.1 | -12.2 | -1.7 | -2.7 | -2.9 | -5.9 | -6.9 | -3.3 | -6.2 | -16.7 | -2.9 | -11.5 | -8.8 | -5.0 | **-5.0** | **-8.8** | **-3.3** |
| LAB(52-5°N) | 1.6 | -0.1 | -5.5 | -0.2 | 4.4 | 9.0 | 1.0 | 0.3 | 0.2 | -0.4 | 0.3 | -1.1 | 5.7 | 1.7 | 1.3 | 0.8 | 0.6 | 0.8 | -6.0 | 0.0 | 0.3 | 2.0 | 2.4 | 6.6 | 1.7 | **0.8** | **0.0** | **1.7** |
| AC(34-30°S) | 4.4 | 3.3 | 1.8 | 1.2 | 4.5 | 4.5 | 5.0 | 4.2 | 1.8 | 1.5 | 3.9 | 1.8 | 5.6 | 2.7 | 0.2 | 3.6 | 3.1 | 5.6 | 4.5 | 1.9 | 11.5 | 1.4 | 8.4 | 9.7 | 4.8 | **3.9** | **1.8** | **4.8** |
| AC(20-15°S) | 1.9 | 0.3 | 2.1 | 1.3 | 4.3 | 4.3 | 4.4 | 2.8 | 3.7 | 3.5 | 0.8 | 2.1 | 4.6 | 0.1 | 0.3 | 3.9 | 3.8 | 2.9 | 2.8 | 3.0 | 6.1 | 2.0 | 4.3 | 3.8 | 2.9 | **2.9** | **2.0** | **3.9** |
| EACC(10-5°S) | 0.4 | 0.4 | -0.2 | -0.3 | -1.0 | -0.5 | -1.1 | -1.1 | 0.7 | 0.5 | -1.0 | 0.1 | 1.0 | -0.4 | -0.4 | -0.6 | 0.7 | 0.9 | 0.7 | 0.3 | -0.2 | 0.9 | 1.3 | 1.1 | -0.3 | **(0.1)** | **-0.4** | **0.7** |
| MNC(14-11°S) | -1.3 | -1.6 | -1.2 | -0.7 | -3.2 | -2.6 | -2.7 | -3.1 | -1.2 | -0.9 | -1.7 | -1.2 | -1.7 | -1.6 | -0.9 | -2.2 | -2.4 | -1.2 | -1.4 | -2.3 | -2.0 | -0.3 | -1.4 | -2.4 | -2.8 | **-1.6** | **-2.4** | **-1.2** |
| SMC(28-23°S) | 1.1 | 2.0 | 0.6 | 0.4 | 1.0 | 0.7 | 0.7 | 1.3 | 0.0 | 0.0 | 0.5 | 0.8 | 1.0 | 0.3 | 1.8 | 0.4 | 1.1 | 0.4 | 0.9 | 0.7 | 0.7 | 0.9 | 0.8 | 1.5 | 0.6 | **0.7** | **0.5** | **1.0** |
| ATL18(18-22°N) | -0.8 | -1.8 | -1.5 | -0.8 | -2.8 | -3.4 | -2.5 | -3.1 | -6.3 | -5.6 | -0.9 | -2.2 | -1.0 | -2.3 | -1.7 | -3.4 | -0.3 | -0.6 | -1.3 | -0.2 | -4.5 | -1.1 | -2.9 | -2.0 | -2.6 | **-2.0** | **-2.9** | **-1.0** |
| ATL25(25-30°N) | -5.5 | -3.8 | -4.3 | 0.0 | -9.0 | -8.7 | -10.3 | -4.9 | -5.5 | -5.0 | -3.2 | -1.1 | -14.1 | -3.7 | -3.8 | -3.9 | -5.3 | -6.3 | -4.3 | -3.5 | -14.9 | -3.0 | -11.3 | -10.0 | -3.9 | **-4.9** | **-8.7** | **-3.8** |

*Table S5 0-1000m integrated projected transport change (ssp585) for currents described in the text (CMIP6 models). Median change is significant at 95% level using a two-sided Wilcoxon signed rank test unless in parenthesis*

| SSP585 change | ACCESS-CM2 | ACCESS-ESM1-5 | BCC-CSM2-MR | CAMS-CSM1-0 | CESM2-WACCM | CESM2 | CIESM | CMCC-CM2-SR5 | CNRM-CM6-1 | CNRM-ESM2-1 | CanESM5 | EC-Earth3-Veg | GISS-E2-1-G | INM-CM4-8 | INM-CM5-0 | IPSL-CM6A-LR | MIROC-ES2L | MIROC6 | MPI-ESM1-2-HR | MPI-ESM1-2-LR | MRI-ESM2-0 | NESM3 | NorESM2-LM | NorESM2-MM | UKESM1-0-LL | **Median** | **25th percentile** | **75th percentile** | |
|---|---|---|---|---|---|---|---|---|---|---|---|---|---|---|---|---|---|---|---|---|---|---|---|---|---|---|---|---|---|
| ITF | 2.4 | 2.0 | 0.7 | 1.1 | 3.1 | 3.0 | 3.2 | 2.8 | 3.0 | 2.4 | 5.7 | 2.7 | 3.3 | 2.3 | 0.9 | 3.9 | 3.0 | 2.9 | 3.0 | 3.1 | 6.5 | 0.9 | 4.0 | 4.4 | 3.2 | **3.0** | **2.4** | **3.2** | |
| ACx(25°E) | -6.9 | -3.4 | -6.0 | -5.0 | 2.5 | 1.9 | 2.3 | -9.8 | -7.6 | -7.2 | -3.6 | -4.1 | -7.7 | -4.3 | -1.3 | -7.4 | -10.6 | -9.6 | -2.7 | -6.9 | -6.0 | -4.0 | -8.9 | -9.7 | -10.1 | **-6.0** | **-7.7** | **-3.6** | |
| KC(140°E) | -2.8 | -0.9 | 0.8 | -1.5 | -2.6 | -3.3 | -1.4 | -2.9 | -1.8 | -0.9 | -16.9 | -3.6 | -3.7 | 1.6 | -0.9 | -1.6 | -0.9 | -1.6 | -3.5 | -0.7 | -9.3 | -2.3 | -5.9 | -4.9 | -2.6 | **-2.3** | **-3.5** | **-0.9** | |
| KC(150°E) | -2.9 | -1.9 | 0.4 | -1.2 | -2.1 | -2.8 | -0.1 | -3.8 | -1.3 | -0.5 | -23.2 | -2.5 | -5.1 | -2.6 | -1.6 | -1.4 | 0.3 | -1.1 | 0.0 | -0.2 | -8.9 | -1.6 | -7.2 | -4.7 | -1.4 | **-1.6** | **-2.9** | **-1.1** | |
| GS(300°E) | -6.7 | -6.7 | -9.2 | -2.0 | -10.9 | -10.5 | -14.0 | -6.9 | -6.8 | -4.7 | -17.6 | -3.1 | -19.6 | -3.0 | -1.0 | -2.7 | -6.9 | -8.9 | -6.6 | -7.4 | -20.2 | -2.4 | -14.1 | -13.3 | -6.6 | **-6.9** | **-10.9** | **-4.7** | |
| TASL(146°E) | -8.0 | -2.7 | -3.1 | -3.0 | -5.4 | -6.2 | -5.3 | -1.6 | -4.6 | -4.3 | -17.9 | -4.6 | -2.9 | -4.4 | -7.3 | -8.3 | -8.0 | -7.4 | -4.5 | -7.4 | -14.4 | -1.7 | -4.4 | -4.3 | -9.9 | **-4.6** | **-7.4** | **-4.3** | |
| EAC(30-25°S) | 1.2 | -1.7 | 1.5 | 0.3 | 0.9 | -0.6 | -1.0 | -0.7 | -1.3 | -1.5 | 5.9 | -1.5 | 0.3 | 1.7 | 3.6 | 0.3 | 0.6 | 0.5 | 0.3 | 1.1 | 4.2 | -1.4 | 0.0 | 0.3 | 1.3 | **(0.3)** | **-0.7** | **1.2** | |
| EACx(40-35°S) | -8.2 | -5.1 | -2.3 | -4.3 | -9.1 | -6.5 | -4.9 | -3.2 | -5.0 | -5.4 | -11.2 | -4.7 | -7.7 | -4.6 | -2.1 | -6.6 | -7.0 | -6.1 | -5.9 | -2.5 | -11.7 | -3.6 | -5.3 | -4.8 | -8.2 | **-5.3** | **-7.0** | **-4.6** | |
| MC(7-10°N) | 3.4 | 3.4 | 2.4 | 0.9 | 8.2 | 4.5 | 0.9 | 6.6 | 3.5 | 3.3 | 12.0 | 4.4 | 2.3 | 0.3 | 0.5 | 5.8 | 4.5 | 4.1 | 2.3 | 0.8 | 9.2 | 2.9 | 4.3 | 5.6 | 5.9 | **3.5** | **2.3** | **5.6** | |
| KC(20-25°N) | -3.2 | -2.1 | -0.6 | -1.7 | -4.2 | -2.6 | 0.4 | -2.4 | -2.7 | -3.7 | -9.2 | -4.5 | -3.0 | -0.2 | 0.0 | -2.7 | -3.8 | -3.9 | -2.0 | -1.3 | -7.1 | -4.3 | -5.0 | -3.9 | -4.4 | **-3.0** | **-4.2** | **-2.0** | |
| KC(25-30°N) | -2.0 | -0.2 | 0.9 | -0.6 | 0.6 | -1.3 | -1.8 | 0.2 | -2.1 | -0.9 | -10.0 | -3.7 | -1.8 | -5.0 | -1.7 | -1.7 | -0.7 | -2.4 | 0.2 | 0.4 | -8.4 | -0.8 | -5.6 | -4.1 | -1.4 | **-1.7** | **-2.4** | **-0.6** | |
| KC(35-40°S) | 1.8 | -2.5 | -0.4 | 2.3 | 2.8 | 0.4 | -0.7 | 0.6 | 2.0 | 2.7 | 4.9 | 0.7 | 5.1 | 3.3 | 2.2 | 0.8 | 1.3 | 2.9 | 2.2 | 2.6 | 4.6 | 2.2 | 4.5 | 4.1 | -0.2 | **2.2** | **0.7** | **2.9** | |
| OC(50-55°N) | -0.6 | 0.0 | -0.6 | -0.1 | -1.1 | -0.2 | -2.7 | 0.6 | -0.8 | -0.7 | 0.8 | 0.1 | 1.2 | -0.3 | 1.2 | -0.9 | 0.0 | -0.7 | -1.4 | -1.7 | -1.9 | -2.1 | 1.1 | 0.6 | -2.9 | **(-0.6)** | **-1.1** | **0.1** | |
| GPC(15-13°S) | 2.3 | 2.3 | 1.5 | 3.2 | 2.2 | 2.8 | 5.1 | -2.0 | 2.3 | 3.1 | -5.4 | 2.5 | 5.7 | -1.2 | -0.6 | 1.7 | 2.8 | 1.6 | 2.1 | 1.7 | 0.0 | 3.3 | -2.0 | 0.6 | 1.2 | **2.1** | **0.6** | **2.8** | |
| NZ(38-34°S) | 6.1 | 2.4 | 3.2 | 2.2 | 7.7 | 4.1 | 4.1 | 1.6 | -1.1 | -1.7 | 13.0 | -1.2 | 5.1 | 6.0 | 7.2 | 1.6 | 1.8 | 2.8 | 4.0 | 5.1 | 8.2 | -1.3 | 2.1 | 2.5 | 4.0 | **3.2** | **1.8** | **5.1** | |
| NZ(50-46°S) | -1.7 | -0.7 | -0.1 | -0.1 | -8.3 | -3.3 | -3.7 | 0.0 | 1.9 | 2.7 | -11.3 | 2.1 | -2.0 | 3.3 | 1.5 | 0.6 | 4.5 | -2.2 | -5.5 | 0.0 | -4.4 | 2.0 | -1.4 | -1.9 | 0.1 | **(-0.1)** | **-2.2** | **1.5** | |
| NGC(10-5°S) | 1.9 | 3.7 | 0.7 | 2.4 | 12.1 | 6.0 | 3.5 | 0.8 | 2.4 | 2.8 | 5.3 | 4.6 | 4.9 | 2.5 | 2.0 | 1.7 | 6.1 | 4.7 | 1.9 | 1.8 | 5.1 | 5.3 | 3.2 | 2.9 | 1.3 | **2.9** | **1.9** | **4.9** | |
| BC(40-35°S) | -5.5 | -3.6 | -5.4 | -2.7 | -10.5 | -5.4 | -7.6 | -5.2 | -4.2 | -3.0 | -5.9 | -4.4 | -6.0 | -7.1 | -1.8 | -4.5 | -5.2 | -4.5 | -6.9 | -7.4 | -17.4 | -4.1 | -5.0 | -5.1 | -2.6 | **-5.2** | **-6.0** | **-4.2** | |
| NBC(10-5°S) | -2.5 | -1.5 | -4.0 | -0.8 | -9.8 | -4.7 | -5.5 | -3.5 | -3.1 | -2.1 | -5.3 | 0.2 | -9.0 | -1.8 | -2.0 | -1.2 | -2.3 | -2.5 | -2.2 | -1.7 | -9.5 | 2.1 | -5.3 | -4.7 | -1.2 | **-2.5** | **-4.7** | **-1.7** | |
| GS(28-33°N) | -7.3 | -5.6 | -9.6 | -1.6 | -18.0 | -9.4 | -10.8 | -5.6 | -4.3 | -4.3 | -19.2 | -3.6 | -16.1 | -3.2 | -5.1 | -4.9 | -7.2 | -8.6 | -5.6 | -9.5 | -19.0 | -4.0 | -13.3 | -11.3 | -6.9 | **-7.2** | **-10.8** | **-4.9** | |
| LAB(52-5°N) | -0.8 | -0.6 | -4.3 | -0.9 | 8.1 | 8.3 | 0.7 | -0.1 | 0.0 | -1.2 | 7.8 | -0.9 | 5.5 | 3.6 | -0.4 | 1.4 | 1.1 | 0.8 | -6.1 | 0.0 | -0.4 | 2.4 | 2.4 | 7.1 | 2.5 | **(0.7)** | **-0.6** | **2.5** | |
| AC(34-30°S) | 7.8 | 5.0 | 3.1 | 3.4 | 14.0 | 5.6 | 5.7 | 4.6 | 2.1 | 0.8 | 20.3 | 3.4 | 7.2 | 3.5 | 0.7 | 4.7 | 4.0 | 7.6 | 5.8 | 2.6 | 15.5 | -0.1 | 9.7 | 11.2 | 7.2 | **5.0** | **3.4** | **7.6** | |
| AC(20-15°S) | 3.2 | 1.0 | 3.5 | 2.4 | 7.2 | 5.2 | 4.5 | 3.8 | 4.9 | 3.8 | 5.9 | 3.3 | 5.8 | 0.1 | 0.4 | 6.0 | 4.9 | 4.4 | 3.4 | 4.6 | 10.7 | 2.4 | 5.1 | 5.3 | 5.3 | **4.5** | **3.3** | **5.3** | |
| EACC(10-5°S) | -0.8 | -0.4 | -0.3 | -0.7 | -4.4 | -1.0 | -2.5 | -2.3 | 0.1 | 0.7 | -7.7 | -0.7 | -0.3 | -1.2 | -0.5 | -1.7 | 0.5 | 0.6 | 0.5 | -0.3 | -2.2 | 1.2 | 0.9 | 0.4 | -2.2 | **-0.5** | **-1.7** | **0.4** | |
| MNC(14-11°S) | -2.9 | -2.8 | -2.0 | -1.7 | -9.0 | -3.6 | -3.9 | -4.6 | -2.0 | -1.0 | -11.4 | -2.2 | -3.2 | -2.4 | -1.3 | -3.9 | -3.4 | -2.3 | -2.1 | -3.8 | -5.5 | -0.2 | -2.2 | -3.4 | -5.0 | **-2.9** | **-3.9** | **-2.1** | |
| SMC(28-23°S) | 2.1 | 2.9 | 1.1 | 1.0 | 4.4 | 1.1 | 1.4 | 1.6 | -0.1 | 0.1 | 7.0 | 1.4 | 1.8 | 0.8 | 1.6 | 0.8 | 1.5 | 0.8 | 1.6 | 0.9 | 3.3 | 0.1 | 1.6 | 2.0 | 1.1 | **1.4** | **0.9** | **1.8** | |
| ATL18(18-22°N) | -1.2 | -2.3 | -3.2 | -1.6 | -5.6 | -3.6 | -3.0 | -3.3 | -7.1 | -6.3 | -4.9 | -4.6 | -1.4 | -3.9 | -2.7 | -5.2 | -0.6 | -0.7 | -2.0 | -0.3 | -6.5 | -2.2 | -3.6 | -2.8 | -3.4 | **-3.2** | **-4.6** | **-2.0** | |
| ATL25(25-30°N) | -7.8 | -5.4 | -9.0 | -1.5 | -17.1 | -9.2 | -11.2 | -5.4 | -5.9 | -5.3 | -15.5 | -1.7 | -18.2 | -6.5 | -6.4 | -6.1 | -6.9 | -7.9 | -7.0 | -5.9 | -17.9 | -5.1 | -12.7 | -12.7 | -5.7 | **-6.9** | **-11.2** | **-5.7** | |

*Table S6 0-100m integrated historical transports for currents described in the text (CMIP5 models)*

| **Historical** | ACCESS1-0 | ACCESS1-3 | CCSM4 | CESM1-BGC | CESM1-CAM5-1-FV2 | CESM1-CAM5 | CMCC-CESM | CMCC-CMS | CMCC-CM | CNRM-CM5 | CanESM2 | FIO-ESM | GFDL-CM3 | GFDL-ESM2G | GFDL-ESM2M | HadGEM2-AO | IPSL-CM5A-LR | IPSL-CM5A-MR | IPSL-CM5B-LR | MIROC-ESM-CHEM | MIROC-ESM | MIROC5 | MPI-ESM-LR | MPI-ESM-MR | MRI-CGCM3 | MRI-ESM1 | NorESM1-ME | NorESM1-M | **Median** | **25th percentile** | **75th percentile** | **Reanalysis mean** |
|---|---|---|---|---|---|---|---|---|---|---|---|---|---|---|---|---|---|---|---|---|---|---|---|---|---|---|---|---|---|---|---|---|
| ITF | -7.9 | -8.6 | -6.7 | -6.8 | -5.5 | -6.2 | -6.2 | -8.2 | -7.6 | -4.8 | -7.0 | -5.2 | -6.8 | -6.0 | -6.1 | -6.8 | -7.4 | -7.4 | -7.5 | -7.8 | -7.8 | -7.1 | -5.6 | -8.3 | -5.8 | -5.7 | -7.4 | -6.5 | **-6.8** | **-7.5** | **-6.1** | **6.2** |
| ACx(25°E) | -8.6 | -8.6 | -9.9 | -11.0 | -10.3 | -9.9 | -3.8 | -4.3 | -4.8 | -6.3 | -6.2 | -8.6 | -7.3 | -5.4 | -4.9 | -7.8 | -1.0 | -1.3 | -2.3 | -5.1 | -6.1 | -5.4 | -9.9 | -11.7 | -5.3 | -5.4 | -5.5 | -4.6 | **-5.8** | **-8.6** | **-4.9** | **8.9** |
| KC(140°E) | 7.4 | 7.3 | 9.4 | 9.5 | 9.4 | 9.0 | 9.6 | 8.8 | 10.0 | 8.8 | 10.5 | 9.7 | 7.8 | 9.2 | 8.2 | 7.0 | 10.1 | 10.3 | 9.7 | 10.4 | 10.3 | 11.0 | 10.6 | 8.7 | 7.8 | 7.9 | 12.2 | 12.5 | **9.5** | **8.6** | **10.3** | **-8.2** |
| KC(150°E) | 8.4 | 9.1 | 10.6 | 10.5 | 10.8 | 10.4 | 11.2 | 11.7 | 12.1 | 10.5 | 12.9 | 13.9 | 9.8 | 9.8 | 10.3 | 8.8 | 10.8 | 11.0 | 11.0 | 14.2 | 14.2 | 12.2 | 12.2 | 10.8 | 9.8 | 10.1 | 14.3 | 14.3 | **10.8** | **10.2** | **12.2** | **-10.4** |
| GS(300°E) | 8.3 | 8.1 | 9.3 | 9.3 | 8.0 | 8.4 | 7.2 | 6.9 | 6.7 | 6.8 | 10.1 | 6.1 | 7.7 | 6.9 | 8.0 | 5.7 | 6.4 | 7.2 | 3.9 | 10.2 | 10.0 | 9.7 | 9.0 | 7.1 | 5.5 | 5.4 | 11.8 | 11.9 | **7.9** | **6.8** | **9.3** | **-8.5** |
| TASL(146°E) | 0.9 | 0.5 | 0.5 | 0.4 | 0.2 | 0.3 | 1.0 | 1.4 | 1.7 | 1.8 | 1.5 | 2.4 | 1.5 | 0.1 | -0.2 | 0.2 | 1.0 | 1.7 | 1.1 | 3.7 | 4.4 | 1.7 | 3.4 | 1.0 | 0.9 | 1.3 | 0.7 | 0.7 | **1.0** | **0.5** | **1.7** | **0.5** |
| EAC(30-25°S) | -2.9 | -2.3 | -3.2 | -3.1 | -3.8 | -3.4 | -4.1 | -4.0 | -3.5 | -3.4 | -4.2 | -4.1 | -4.6 | -2.4 | -4.6 | -2.0 | -6.0 | -5.6 | -5.4 | -3.1 | -3.3 | -4.8 | -7.1 | -2.4 | -2.9 | -3.0 | -4.3 | -4.7 | **-3.6** | **-4.6** | **-3.1** | **1.5** |
| EACx(40-35°S) | 0.7 | 0.6 | -0.2 | -0.1 | -0.4 | 0.0 | 0.7 | 0.7 | 0.2 | -0.1 | -0.1 | 0.4 | 0.5 | -0.1 | 0.2 | -0.3 | 1.0 | 0.5 | 1.8 | 1.7 | 1.9 | 0.7 | 1.4 | 0.0 | 0.5 | 0.4 | -0.6 | -0.6 | **0.4** | **-0.1** | **0.7** | **1.1** |
| MC(7-10°N) | -6.3 | -9.0 | -7.4 | -6.6 | -8.9 | -7.8 | -5.0 | -6.4 | -7.6 | -11.7 | -6.1 | -9.9 | -3.5 | -1.3 | -3.9 | -4.1 | -4.6 | -4.2 | -3.4 | -8.0 | -7.5 | -3.5 | 1.7 | -4.9 | -2.2 | -2.2 | -10.6 | -10.1 | **-6.2** | **-7.9** | **-3.8** | **8.6** |
| KC(20-25°N) | 5.6 | 3.9 | 7.7 | 8.0 | 8.1 | 7.0 | 4.0 | 4.6 | 4.5 | 6.7 | 6.4 | 9.0 | 5.1 | 5.3 | 6.4 | 2.9 | 4.4 | 4.6 | 4.9 | 9.5 | 8.7 | 8.2 | 8.8 | 6.2 | 5.3 | 4.0 | 7.9 | 8.0 | **6.3** | **4.6** | **8.0** | **-5.4** |
| KC(25-30°N) | 5.7 | 6.9 | 8.2 | 8.7 | 10.0 | 9.7 | 6.3 | 7.3 | 7.5 | 9.4 | 8.8 | 10.0 | 7.0 | 8.7 | 7.4 | 3.5 | 5.8 | 6.7 | 6.1 | 11.3 | 11.8 | 10.8 | 10.6 | 8.5 | 8.2 | 7.0 | 11.6 | 11.5 | **8.3** | **6.9** | **10.0** | **-7.3** |
| KC(35-40°S) | 3.6 | 4.6 | 3.2 | 3.0 | 3.2 | 3.2 | 2.4 | 1.8 | 2.0 | 2.9 | 3.7 | 0.7 | 2.3 | 3.4 | 3.4 | 1.8 | 0.8 | 1.9 | -1.9 | 4.5 | 4.5 | 0.9 | 3.8 | 2.3 | 0.7 | 0.7 | 1.8 | 1.5 | **2.4** | **1.7** | **3.4** | **-1.6** |
| OC(50-55°N) | -1.8 | -1.8 | -1.2 | -1.2 | -0.5 | -1.7 | -1.2 | -1.2 | -1.3 | -2.4 | -2.3 | -2.4 | -1.1 | -1.3 | -1.0 | -1.5 | -2.1 | -2.2 | -2.6 | -1.9 | -1.8 | -0.9 | -1.5 | -1.4 | -2.1 | -2.2 | -0.6 | -0.6 | **-1.5** | **-2.1** | **-1.2** | **2.3** |
| GPC(15-13°S) | 0.2 | 1.6 | 2.3 | 2.5 | 4.2 | 2.9 | 3.1 | 0.2 | 0.9 | 2.3 | 3.5 | 5.3 | 2.4 | 2.4 | 2.3 | 4.2 | 0.7 | 0.8 | -1.0 | -1.3 | -1.3 | -0.4 | -0.5 | 1.7 | 3.4 | 3.4 | 0.0 | 0.5 | **2.0** | **0.2** | **2.9** | **-0.3** |
| NZ(38-34°S) | -2.1 | -1.7 | -1.2 | -1.5 | -1.6 | -1.7 | -3.4 | -2.8 | -2.2 | -2.0 | -2.9 | -2.5 | -3.0 | -0.6 | -3.0 | -0.9 | -2.6 | -2.6 | -1.5 | -3.4 | -3.3 | -2.6 | -1.4 | -2.4 | -1.4 | -1.4 | -1.7 | -1.6 | **-2.0** | **-2.7** | **-1.5** | **-0.3** |
| NZ(50-46°S) | 2.7 | 2.4 | 2.9 | 3.6 | 2.4 | 3.1 | 2.7 | 2.7 | 2.8 | 4.1 | 4.1 | 4.8 | 1.5 | 2.5 | 2.4 | 2.7 | 2.3 | 2.5 | 0.9 | 5.4 | 5.2 | 2.0 | 1.1 | 3.1 | 3.4 | 3.2 | 2.6 | 2.5 | **2.7** | **2.4** | **3.3** | **-2.8** |
| NGC(10-5°S) | 2.4 | 2.6 | 1.6 | 1.2 | 0.9 | -1.0 | -5.6 | -1.2 | 2.8 | 4.1 | -0.3 | 9.2 | -0.8 | -1.4 | -0.5 | 0.3 | -1.7 | -2.4 | -1.3 | 2.6 | 2.5 | -1.2 | -5.6 | -3.7 | 0.2 | 0.0 | 2.1 | 1.9 | **0.1** | **-1.2** | **2.2** | **2.2** |
| BC(40-35°S) | -2.5 | -2.8 | -3.5 | -3.4 | -3.9 | -3.8 | -1.3 | -1.2 | -1.0 | -2.0 | -1.6 | -1.7 | -1.8 | 0.6 | 1.2 | -0.8 | -1.7 | -1.7 | -1.5 | -1.5 | -1.5 | -2.6 | -3.1 | -2.4 | -0.6 | -0.6 | -0.7 | -1.3 | **-1.6** | **-2.5** | **-1.2** | **2.3** |
| NBC(10-5°S) | 1.6 | 2.9 | 5.9 | 6.2 | 6.9 | 5.0 | 6.5 | 6.0 | 6.0 | 5.0 | 4.2 | 3.7 | 6.7 | 8.0 | 6.6 | 1.6 | 5.2 | 6.0 | 1.1 | 5.8 | 5.5 | 5.3 | 7.3 | 7.4 | 3.0 | 3.1 | 8.1 | 8.1 | **5.8** | **4.1** | **6.6** | **-2.8** |
| GS(28-33°N) | 9.1 | 8.5 | 8.6 | 8.5 | 8.3 | 8.6 | 4.9 | 4.2 | 5.4 | 6.3 | 8.7 | 6.6 | 6.7 | 7.1 | 7.0 | 5.2 | 4.8 | 7.2 | 3.4 | 7.8 | 7.8 | 10.1 | 8.5 | 7.3 | 5.3 | 5.5 | 10.2 | 10.3 | **7.2** | **5.5** | **8.6** | **-7.7** |
| LAB(52-5°N) | -2.6 | -2.5 | -2.9 | -3.2 | -2.7 | -2.7 | -2.1 | -1.6 | -1.6 | -2.9 | -2.1 | -2.5 | -2.5 | -0.6 | -1.6 | -2.7 | -2.4 | -2.2 | -1.4 | -3.3 | -2.9 | -2.0 |  | -2.2 | -1.3 | -1.4 | -3.5 | -3.2 | **-2.5** | **-2.8** | **-1.8** | **0.5** |
| AC(34-30°S) | -6.7 | -6.8 | -8.6 | -8.7 | -8.2 | -8.3 | -8.6 | -8.7 | -8.7 | -7.4 | -10.9 | -7.7 | -9.2 | -12.3 | -10.0 | -6.9 | -8.2 | -9.0 | -5.9 | -9.8 | -10.1 | -10.0 | -4.5 | -8.6 | -8.1 | -8.0 | -12.2 | -12.4 | **-8.6** | **-9.9** | **-8.0** | **7.6** |
| AC(20-15°S) | -5.3 | -5.1 | -4.6 | -4.4 | -4.3 | -4.3 | -3.7 | -4.6 | -4.4 | -5.0 | -5.0 | -4.5 | -4.2 | -5.0 | -4.2 | -2.3 | -5.3 | -5.2 | -4.0 | -4.1 | -4.1 | -4.3 | -3.8 | -4.3 | -3.3 | -3.3 | -5.1 | -5.2 | **-4.4** | **-5.0** | **-4.1** | **4.4** |
| EACC(10-5°S) | 8.0 | 8.5 | 8.2 | 7.9 | 9.4 | 9.2 | 5.7 | 3.8 | 4.1 | 7.7 | 9.5 | 10.8 | 7.0 | 9.7 | 8.0 | 5.7 | 6.6 | 6.9 | 3.5 | 8.7 | 8.8 | 6.7 | 8.6 | 9.9 | 3.9 | 3.8 | 8.6 | 8.3 | **8.0** | **6.4** | **8.8** | **-8.1** |
| MNC(14-11°S) | 2.3 | 2.8 | 4.3 | 4.1 | 6.2 | 4.7 | 7.0 | 2.9 | 2.9 | 2.9 | 3.8 | 5.5 | 5.0 | 6.1 | 4.9 | 2.2 | 4.2 | 4.6 | 1.8 | 4.2 | 3.6 | 3.8 | 6.8 | 4.8 | 2.8 | 2.4 | 4.4 | 4.0 | **4.2** | **2.9** | **4.8** | **-4.6** |
| SMC(28-23°S) | -1.9 | -2.0 | -2.4 | -2.7 | -3.0 | -2.9 | -2.1 | -1.4 | -1.9 | -0.6 | -1.4 | -1.4 | -1.5 | -1.4 | -1.3 | -2.0 | -0.3 | -0.3 | 0.2 | -0.5 | -0.5 | -1.4 | -1.8 | -1.7 | -1.9 | -1.7 | -1.8 | -2.2 | **-1.7** | **-2.0** | **-1.4** | **1.6** |
| ATL18(18-22°N) | 3.7 | 3.3 | 5.5 | 7.4 | 7.2 | 7.3 | 2.6 | 6.5 | 2.2 | -1.4 | 7.5 | 7.4 | 7.7 | 5.9 | 8.1 | 7.5 | 3.8 | 6.3 | 5.4 | 5.6 | 5.5 | 8.0 | 4.6 | 6.8 | 6.8 | 6.6 | 6.0 | 6.1 | **6.2** | **5.2** | **7.3** | **-6.0** |
| ATL25(25-30°N) | 8.9 | 8.4 | 8.4 | 8.4 | 8.3 | 8.5 | 4.7 | 4.3 | 5.1 | 6.7 | 7.3 | 8.2 | 5.8 | 6.5 | 5.9 | 4.6 | 2.9 | 6.4 | 2.2 | 7.0 | 7.0 | 7.8 | 7.2 | 6.9 | 4.6 | 4.9 | 10.1 | 10.2 | **6.9** | **5.1** | **8.3** | **-7.7** |

*Table S7 0-100m integrated projected transport change (RCP8.5) for currents described in the text (CMIP5 models)*

| Change | ACCESS1-0 | ACCESS1-3 | CCSM4 | CESM1-BGC | CESM1-CAM5-1-FV2 | CESM1-CAM5 | CMCC-CESM | CMCC-CMS | CMCC-CM | CNRM-CM5 | CanESM2 | FIO-ESM | GFDL-CM3 | GFDL-ESM2G | GFDL-ESM2M | HadGEM2-AO | IPSL-CM5A-LR | IPSL-CM5A-MR | IPSL-CM5B-LR | MIROC-ESM-CHEM | MIROC-ESM | MIROC5 | MPI-ESM-LR | MPI-ESM-MR | MRI-CGCM3 | MRI-ESM1 | NorESM1-ME | NorESM1-M | **Median** | **25th percentile** | **75th percentile** |
|---|---|---|---|---|---|---|---|---|---|---|---|---|---|---|---|---|---|---|---|---|---|---|---|---|---|---|---|---|---|---|---|
| ITF | 0.7 | 0.7 | 0.0 | 0.1 | 0.2 | 0.2 | -0.7 | -0.2 | 0.0 | 0.4 | -0.2 | 0.1 | 0.0 | 0.3 | -0.3 | 0.4 | -0.2 | -0.2 | 0.2 | 1.4 | 1.2 | 0.4 | -0.5 | 0.0 | 0.9 | 0.7 | 0.1 | -0.1 | **0.1** | **-0.1** | **0.4** |
| ACx(25°E) | -1.7 | -1.6 | -0.2 | 0.1 | 0.2 | 0.3 | -1.8 | -2.1 | -1.8 | -0.8 | -3.6 | 0.2 | -2.4 | -2.0 | -4.3 | -1.4 | -3.3 | -3.3 | -0.7 | -3.2 | -3.2 | -2.9 | 0.0 | 1.0 | -2.0 | -2.0 | -1.4 | -1.6 | **-1.7** | **-2.5** | **-0.5** |
| KC(140°E) | 0.8 | 1.4 | 0.5 | 0.7 | 0.6 | 0.9 | 0.4 | -0.5 | -0.6 | 0.2 | 1.2 | 0.8 | 0.7 | 0.2 | 0.6 | 0.7 | 0.2 | -0.1 | -0.2 | 0.6 | 0.2 | 0.1 | 0.5 | 0.8 | 0.5 | 0.5 | 0.4 | 0.1 | **0.5** | **0.2** | **0.7** |
| KC(150°E) | 0.9 | 1.4 | 0.5 | 0.6 | 0.7 | 1.0 | 1.8 | 0.9 | 0.0 | 0.6 | 1.4 | 1.3 | 1.3 | 0.5 | 0.8 | 0.7 | 0.7 | 0.5 | -0.2 | 0.7 | 0.5 | 0.2 | 1.2 | 1.8 | 0.9 | 0.9 | 0.3 | 0.3 | **0.7** | **0.5** | **1.0** |
| GS(300°E) | -3.4 | -3.2 | -1.5 | -1.5 | -1.9 | -2.1 | -0.8 | -0.7 | -0.9 | -1.8 | -3.1 | -3.0 | -2.4 | -2.4 | -2.8 | -1.4 | -0.6 | -1.7 | -0.1 | -2.7 | -2.5 | -3.4 | -2.4 | -2.2 | -0.1 | 0.0 | -1.3 | -1.2 | **-1.8** | **-2.6** | **-1.1** |
| TASL(146°E) | -0.9 | -0.2 | -0.2 | -0.2 | -0.6 | -0.4 | -0.4 | -0.9 | -0.8 | -0.5 | -1.2 | -1.0 | -1.0 | -1.1 | -0.4 | -0.4 | -0.4 | -1.1 | 0.1 | -1.5 | -1.7 | -1.1 | -2.1 | -0.5 | -0.7 | -0.6 | -0.4 | -0.4 | **-0.6** | **-1.0** | **-0.4** |
| EAC(30-25°S) | -0.1 | -0.5 | -0.3 | -0.3 | -0.1 | -0.5 | -0.6 | -0.2 | 0.1 | 0.1 | 0.0 | -0.4 | -0.2 | 0.2 | 0.2 | -0.4 | 1.0 | 1.3 | -0.6 | 0.2 | 0.2 | 0.5 | 1.5 | -0.3 | -0.2 | -0.4 | 0.0 | 0.5 | **-0.1** | **-0.3** | **0.2** |
| EACx(40-35°S) | -0.4 | -0.3 | -0.5 | -0.6 | -1.0 | -0.7 | -0.5 | -0.9 | -0.8 | -0.4 | -1.2 | -0.4 | -0.2 | -0.2 | -0.5 | -0.9 | -0.9 | -1.1 | -0.3 | -1.1 | -1.2 | -1.2 | -0.8 | -0.7 | -0.6 | -0.5 | -0.3 | -0.5 | **-0.6** | **-0.9** | **-0.4** |
| MC(7-10°N) | -2.1 | -2.5 | 0.1 | 0.1 | -1.5 | -0.7 | -2.7 | -0.4 | 0.2 | 1.0 | 0.9 | -2.8 | -0.2 | -0.3 | 0.1 | 0.1 | 2.2 | 1.7 | -0.5 | -3.4 | -3.2 | -0.5 | -1.2 | -1.0 | -0.2 | -0.3 | -0.6 | -0.5 | **-0.4** | **-1.3** | **0.1** |
| KC(20-25°N) | 0.4 | 0.7 | 0.5 | 0.3 | 0.7 | 0.5 | -0.2 | -0.1 | -0.4 | -0.2 | 0.0 | 0.8 | 0.1 | -0.3 | -0.1 | -0.2 | -0.3 | -0.2 | -0.3 | 1.2 | 1.2 | 0.2 | 0.3 | 0.2 | 0.5 | 0.2 | 0.5 | 0.5 | **0.2** | **-0.2** | **0.5** |
| KC(25-30°N) | 0.1 | 0.8 | -0.2 | 0.1 | 0.1 | 0.2 | 0.3 | 0.3 | -0.2 | 0.1 | 0.9 | 0.7 | 0.5 | -0.3 | 0.1 | 0.1 | 0.0 | 0.1 | 0.0 | 0.8 | 0.6 | 0.3 | 0.3 | 0.5 | 0.8 | 0.5 | 0.2 | -0.1 | **0.2** | **0.1** | **0.5** |
| KC(35-40°S) | 0.8 | 0.4 | 0.2 | 0.2 | 0.5 | 0.1 | 1.2 | 1.6 | 1.5 | -0.1 | -0.2 | 0.1 | 0.9 | 0.7 | 0.7 | -0.1 | 0.9 | 1.7 | 0.7 | 0.9 | 1.1 | 0.8 | 1.2 | 1.0 | 0.1 | 0.0 | 0.0 | 0.2 | **0.7** | **0.1** | **0.9** |
| OC(50-55°N) | -0.5 | -0.6 | -0.3 | -0.3 | 0.0 | -0.2 | -0.1 | -0.2 | -0.2 | -0.3 | -1.2 | -0.8 | -0.6 | -0.5 | -0.4 | -0.3 | -0.7 | -0.5 | -0.4 | -1.2 | -1.4 | -0.1 | -0.5 | -0.7 | -0.3 | -0.4 | 0.1 | 0.0 | **-0.4** | **-0.6** | **-0.2** |
| GPC(15-13°S) | 1.2 | 1.6 | -0.1 | 0.4 | 0.4 | 0.7 | 1.2 | 1.8 | 1.4 | 1.2 | 0.0 | 1.6 | 1.7 | 0.8 | 0.7 | 0.7 | 1.3 | 0.3 | 1.5 | 0.1 | -0.1 | 0.2 | 3.5 | 2.0 | 0.5 | 0.7 | -0.1 | 0.0 | **0.7** | **0.2** | **1.4** |
| NZ(38-34°S) | 0.7 | 0.5 | 0.5 | 0.5 | 0.6 | 0.4 | -0.5 | 0.0 | -0.1 | 0.1 | 1.0 | 0.6 | 0.7 | 0.2 | 1.0 | 0.3 | 0.3 | 0.7 | -0.4 | 0.8 | 0.7 | 1.1 | 0.6 | 0.8 | 0.2 | 0.2 | 0.0 | 0.0 | **0.5** | **0.2** | **0.7** |
| NZ(50-46°S) | 0.0 | -0.3 | -0.7 | -1.0 | -0.6 | -0.1 | 0.9 | 0.4 | 0.3 | 0.3 | -0.1 | -0.5 | 0.2 | 0.3 | 0.4 | 0.2 | 0.6 | 0.2 | 0.9 | 0.4 | 0.6 | -0.1 | -0.3 | -0.2 | 0.2 | 0.2 | 0.0 | -0.1 | **0.2** | **-0.2** | **0.4** |
| NGC(10-5°S) | 1.8 | 3.4 | 0.2 | 0.6 | 2.6 | 2.6 | 8.1 | 3.3 | 1.3 | 0.3 | 3.3 | 1.7 | 1.0 | 0.9 | 0.3 | 1.5 | -0.2 | -0.1 | 2.5 | 3.1 | 2.6 | 3.8 | 3.7 | 0.5 | 3.2 | 3.2 | -0.2 | -0.4 | **1.7** | **0.4** | **3.2** |
| BC(40-35°S) | -1.1 | -0.8 | -1.6 | -1.6 | -1.0 | -1.2 | 0.0 | -0.3 | -0.3 | -0.2 | -0.4 | -1.1 | -1.3 | -1.6 | -1.6 | -1.4 | -0.1 | 0.0 | 0.0 | -1.0 | -1.0 | -0.8 | -0.8 | -1.8 | -0.4 | -0.4 | 0.6 | 1.4 | **-0.8** | **-1.2** | **-0.3** |
| NBC(10-5°S) | 0.3 | 0.1 | -0.4 | -0.7 | -0.8 | -0.3 | -0.8 | -0.1 | -0.1 | -0.5 | -0.1 | -1.2 | -1.3 | -1.1 | -1.3 | -0.4 | -0.8 | -0.9 | -0.3 | 0.4 | 0.9 | -0.4 | -0.2 | 0.3 | -0.5 | -0.6 | -1.0 | -0.9 | **-0.5** | **-0.9** | **-0.1** |
| GS(28-33°N) | -3.0 | -2.5 | -0.8 | -0.8 | -0.9 | -1.4 | -0.2 | 0.0 | -0.2 | -1.1 | -2.4 | -3.3 | -1.6 | -2.0 | -1.9 | -1.0 | -0.4 | -0.9 | 0.1 | -1.4 | -1.3 | -2.4 | -3.1 | -2.2 | 0.4 | 0.7 | -1.1 | -1.1 | **-1.1** | **-2.1** | **-0.7** |
| LAB(52-5°N) | 0.4 | 0.1 | 0.6 | 1.2 | 1.3 | 1.6 | -0.1 | -0.2 | -0.2 | 0.0 | -0.1 | 0.4 | 0.3 | -0.7 | 0.3 | 1.0 | -0.4 | 0.1 | 0.4 | -0.7 | -0.5 | 0.2 | 0.0 | -0.8 | 0.1 | -0.1 | 1.3 | 1.0 | **0.1** | **-0.1** | **0.5** |
| AC(34-30°S) | 0.8 | 0.8 | -0.1 | 0.1 | -0.3 | 0.0 | -0.4 | -0.1 | 0.2 | 0.2 | 0.9 | 0.5 | 1.8 | 1.5 | 2.3 | 0.5 | -0.4 | 0.4 | -0.1 | 0.3 | 0.3 | 1.9 | 0.1 | 1.2 | 0.4 | 0.3 | 0.9 | 0.7 | **0.4** | **0.0** | **0.8** |
| AC(20-15°S) | 0.3 | -0.2 | 0.0 | 0.0 | 0.1 | -0.2 | -0.1 | 0.2 | 0.2 | 0.5 | 0.0 | 0.3 | 0.4 | 0.9 | 0.4 | 0.0 | 0.4 | 0.2 | 0.0 | -0.3 | -0.4 | 0.2 | 0.2 | 0.1 | 0.2 | 0.1 | 0.3 | 0.2 | **0.2** | **0.0** | **0.3** |
| EACC(10-5°S) | 0.0 | -0.5 | -1.4 | -1.3 | -0.8 | -1.0 | -0.1 | 0.7 | 0.2 | -0.3 | -2.5 | -1.2 | 0.0 | 1.2 | -0.5 | -0.1 | 0.2 | -0.4 | 0.3 | 0.8 | 0.8 | 0.7 | 0.5 | -0.1 | -0.1 | -0.1 | 0.1 | -0.3 | **-0.1** | **-0.5** | **0.2** |
| MNC(14-11°S) | -1.0 | -1.8 | -1.0 | -0.9 | -0.5 | -1.8 | -0.3 | 0.4 | 0.0 | 0.0 | -1.6 | -1.0 | -0.5 | 0.3 | -0.6 | -0.9 | 0.5 | 0.1 | 0.2 | 0.9 | 0.9 | -0.6 | -1.4 | -0.5 | -0.9 | -0.6 | 0.6 | 0.3 | **-0.5** | **-0.9** | **0.2** |
| SMC(28-23°S) | 0.2 | 0.7 | 0.3 | 0.4 | 0.2 | 0.6 | 0.0 | -0.3 | -0.3 | -0.2 | -0.1 | 0.2 | -0.5 | -0.4 | 0.2 | 0.1 | 0.0 | -0.4 | -0.1 | -0.2 | -0.2 | 0.1 | 0.1 | -0.2 | 0.1 | 0.1 | 0.0 | 0.2 | **0.1** | **-0.2** | **0.2** |
| ATL18(18-22°N) | -0.8 | -0.4 | -0.4 | -0.6 | -0.8 | -0.7 | -0.1 | 0.0 | 0.0 | 0.3 | 0.2 | -0.9 | -1.9 | -0.4 | -1.4 | -0.8 | 0.0 | 0.0 | 0.1 | 0.0 | 0.0 | -0.8 | 0.7 | -0.2 | -0.2 | -0.1 | -0.3 | -0.2 | **-0.2** | **-0.7** | **0.0** |
| ATL25(25-30°N) | -3.2 | -2.6 | -1.1 | -1.0 | -1.1 | -1.5 | -0.2 | -0.1 | -0.2 | -1.5 | -2.3 | -3.9 | -1.7 | -2.0 | -1.6 | -1.1 | -0.6 | -1.1 | -0.1 | -1.7 | -1.5 | -2.0 | -1.9 | -2.1 | 0.3 | 0.5 | -1.1 | -1.1 | **-1.3** | **-1.9** | **-0.9** |

*Table S8 0-100m integrated historical transports for currents described in the text (CMIP6 models)*

| **Historical** | ACCESS-CM2 | ACCESS-ESM1-5 | BCC-CSM2-MR | CAMS-CSM1-0 | CESM2-WACCM | CESM2 | CIESM | CMCC-CM2-SR5 | CNRM-CM6-1 | CNRM-ESM2-1 | CanESM5 | EC-Earth3-Veg | GISS-E2-1-G | INM-CM4-8 | INM-CM5-0 | IPSL-CM6A-LR | MIROC-ES2L | MIROC6 | MPI-ESM1-2-HR | MPI-ESM1-2-[illegible] | MRI-ESM2-0 | NESM3 | NorESM2-LM | NorESM2-MM | UKESM1-0-LL | **Median** | **25th percentile** | **75th percentile** | **Reanalysis mean** |
|---|---|---|---|---|---|---|---|---|---|---|---|---|---|---|---|---|---|---|---|---|---|---|---|---|---|---|---|---|
| ITF | -8.8 | -8.4 | -8.0 | -6.4 | -7.9 | -7.8 | -6.9 | -6.7 | -7.1 | -6.8 | -6.5 | -6.8 | -5.7 | -5.3 | -6.2 | -8.1 | -4.1 | -6.4 | -6.4 | -4.0 | -6.8 | -6.0 | -7.2 | -7.4 | -8.3 | **-6.8** | **-7.8** | **-6.4** | **6.2** |
| ACx(25°E) | -9.3 | -10.3 | -4.0 | -5.3 | -10.1 | -9.3 | -7.3 | -9.0 | -7.1 | -6.9 | -9.3 | -11.3 | -2.9 | -4.5 | -1.1 | -8.9 | -4.3 | -5.9 | -10.8 | -8.4 | -8.5 | -4.2 | -5.2 | -5.6 | -9.2 | **-7.3** | **-9.3** | **-5.2** | **8.9** |
| KC(140°E) | 9.1 | 7.8 | 9.6 | 8.8 | 10.6 | 10.6 | 9.5 | 8.9 | 7.7 | 8.0 | 8.3 | 9.0 | 7.5 | 3.6 | 3.2 | 7.2 | 8.5 | 8.6 | 9.0 | 10.1 | 8.6 | 5.9 | 8.9 | 9.3 | 8.3 | **8.6** | **7.8** | **9.1** | **-8.2** |
| KC(150°E) | 10.2 | 8.8 | 11.0 | 10.4 | 10.6 | 10.5 | 9.6 | 11.0 | 9.8 | 10.2 | 10.1 | 12.0 | 9.2 | 15.9 | 13.5 | 9.6 | 9.2 | 9.4 | 10.7 | 11.6 | 10.4 | 7.2 | 11.5 | 11.7 | 10.6 | **10.4** | **9.6** | **11.0** | **-10.4** |
| GS(300°E) | 6.3 | 6.6 | 10.6 | 6.1 | 7.6 | 7.4 | 9.6 | 6.9 | 9.0 | 8.7 | 6.6 | 7.1 | 9.7 | 6.9 | 5.3 | 6.8 | 6.1 | 6.3 | 7.2 | 7.9 | 7.9 | 5.5 | 9.9 | 10.8 | 6.3 | **7.1** | **6.3** | **8.7** | **-8.5** |
| TASL(146°E) | 0.7 | 0.0 | 0.9 | 0.4 | 0.8 | 0.7 | 0.4 | -0.3 | 1.0 | 0.8 | 1.0 | 0.8 | 0.7 | 0.6 | 0.5 | 1.6 | 1.6 | 0.8 | 0.5 | 1.7 | 1.9 | 0.3 | 0.1 | 0.1 | 1.0 | **0.7** | **0.4** | **1.0** | **0.5** |
| EAC(30-25°S) | -3.3 | -2.5 | -3.4 | -2.7 | -3.7 | -3.7 | -3.0 | -2.8 | -3.6 | -3.7 | -3.3 | -3.5 | -4.1 | -2.6 | -3.3 | -4.3 | -3.9 | -3.3 | -1.7 | -2.5 | -3.2 | -1.9 | -3.6 | -3.3 | -3.8 | **-3.3** | **-3.7** | **-2.8** | **1.5** |
| EACx(40-35°S) | 0.6 | 0.3 | -1.1 | -0.2 | -0.1 | 0.0 | 0.1 | -1.1 | 0.2 | 0.2 | -0.2 | 0.2 | 0.2 | -1.4 | 0.8 | 0.6 | 0.8 | 0.3 | -0.1 | -0.1 | 0.8 | -0.1 | -0.7 | -0.3 | -0.3 | **0.0** | **-0.2** | **0.3** | **1.1** |
| MC(7-10°N) | -5.3 | -9.1 | -6.7 | -1.7 | -8.3 | -7.9 | -7.8 | -9.2 | -7.1 | -7.5 | -6.8 | -5.1 | -0.8 | -2.1 | -1.2 | -5.3 | -10.0 | -8.0 | -5.7 | -1.1 | -3.0 | 1.4 | -5.1 | -6.3 | -6.8 | **-6.3** | **-7.8** | **-3.0** | **8.6** |
| KC(20-25°N) | 6.0 | 4.3 | 7.8 | 5.7 | 6.6 | 7.0 | 7.2 | 3.7 | 3.3 | 8.1 | 5.0 | 6.9 | 4.5 | 0.7 | 1.8 | 3.4 | 7.7 | 6.3 | 5.5 | 6.9 | 4.3 | 3.4 | 4.6 | 3.5 | 5.9 | **5.5** | **3.7** | **6.9** | **-5.4** |
| KC(25-30°N) | 7.5 | 7.1 | 7.5 | 5.7 | 8.2 | 9.2 | 8.4 | 10.1 | 6.9 | 10.0 | 7.2 | 9.3 | 5.8 | -1.1 | 0.6 | 6.5 | 9.6 | 9.6 | 7.0 | 7.2 | 7.9 | 5.5 | 8.7 | 9.0 | 9.7 | **7.5** | **6.9** | **9.2** | **-7.3** |
| KC(35-40°S) | 3.3 | 5.0 | 5.2 | 4.9 | 3.6 | 3.6 | 2.3 | 2.9 | 0.9 | 0.8 | 3.3 | 2.1 | 2.4 | -1.6 | -0.2 | 1.0 | -0.1 | 0.5 | 1.8 | 3.3 | 1.5 | 2.3 | 2.2 | 2.0 | 3.3 | **2.3** | **1.0** | **3.3** | **-1.6** |
| OC(50-55°N) | -2.2 | -1.7 | -1.2 | -1.8 | -1.5 | -1.5 | -1.2 | -2.1 | -1.7 | -1.5 | -1.8 | -2.3 | -1.0 | -0.3 | -1.3 | -2.2 | -1.5 | -1.8 | -1.7 | -2.0 | -1.8 | -1.1 | -1.8 | -1.8 | -2.0 | **-1.7** | **-1.8** | **-1.5** | **2.3** |
| GPC(15-13°S) | 2.5 | 2.3 | 0.6 | 3.3 | 0.4 | 0.7 | -0.6 | 3.6 | 3.8 | 4.2 | 2.0 | 2.6 | 3.6 | 2.3 | 0.9 | 1.2 | -0.3 | -1.1 | 3.9 | 1.2 | 2.2 | 0.1 | 2.2 | -1.3 | 3.5 | **2.2** | **0.6** | **3.3** | **-0.3** |
| NZ(38-34°S) | -2.4 | -1.7 | -2.9 | -1.6 | -1.6 | -1.5 | -1.2 | -1.1 | -1.3 | -1.2 | -1.0 | -0.8 | -1.9 | -0.3 | -0.2 | -1.3 | -3.0 | -1.2 | -1.4 | -2.2 | -2.1 | -0.7 | -1.1 | -1.0 | -1.6 | **-1.3** | **-1.7** | **-1.1** | **-0.3** |
| NZ(50-46°S) | 2.4 | 2.2 | 1.9 | 1.7 | 3.2 | 1.7 | 2.3 | 3.1 | 3.9 | 3.7 | 3.0 | 2.6 | 2.5 | -0.7 | 4.3 | 3.2 | 2.6 | 2.4 | 2.8 |  | 2.6 | 2.1 | 1.4 | 1.5 | 3.5 | **2.5** | **2.0** | **3.1** | **-2.8** |
| NGC(10-5°S) | 4.2 | 1.3 | 1.6 | -3.0 | -0.7 | -0.2 | -0.8 | 1.3 | 3.2 | 3.7 | 0.1 | -1.5 | -0.4 | -0.5 | -0.5 | 0.7 | 2.6 | -0.3 | 1.3 | 0.6 | 1.6 | -4.7 | -0.5 | -2.3 | 1.4 | **0.1** | **-0.5** | **1.4** | **2.2** |
| BC(40-35°S) | -2.5 | -3.1 | -1.2 | -2.5 | -3.8 | -3.4 | -2.8 | -1.7 | -1.4 | -1.5 | -3.6 | -3.4 | 0.1 | -5.6 | -0.1 | -1.9 | -0.7 | -1.1 | -2.8 | -4.7 | -1.0 | -1.7 | -2.2 | -2.2 | -1.8 | **-2.2** | **-3.1** | **-1.4** | **2.3** |
| NBC(10-5°S) | 3.2 | 3.9 | 5.7 | 5.0 | 3.1 | 3.1 | 2.7 | 6.4 | 4.3 | 4.7 | 4.6 | 5.1 | 6.6 | 5.9 | 6.6 | 3.1 | 3.9 | 3.0 | 7.7 | 7.4 | 2.9 | 1.8 | 3.0 | 2.2 | 2.5 | **3.9** | **3.0** | **5.7** | **-2.8** |
| GS(28-33°N) | 7.7 | 6.9 | 8.1 | 6.2 | 7.7 | 4.3 | 7.7 | 7.0 | 9.1 | 8.9 | 7.1 | 7.9 | 9.3 | 5.9 | 6.2 | 8.4 | 6.6 | 6.6 | 6.9 | 6.7 | 8.1 | 6.1 | 8.3 | 8.6 | 7.5 | **7.5** | **6.6** | **8.1** | **-7.7** |
| LAB(52-5°N) | -1.7 | -1.9 | 1.3 | -1.3 | -2.6 | -2.9 | -2.1 | -3.0 | -1.5 | -1.5 | -1.3 | -2.0 | -2.7 | -1.6 | -0.7 | -1.7 | -1.5 | -2.8 | -3.9 |  | -2.3 | -2.0 | -3.4 | -3.6 | -1.9 | **-2.0** | **-2.7** | **-1.5** | **0.5** |
| AC(34-30°S) | -6.7 | -7.0 | -9.0 | -7.4 | -7.5 | -7.6 | -6.6 | -9.2 | -9.6 | -9.7 | -7.3 | -9.5 | -9.9 | -5.6 | -1.5 | -9.3 | -7.4 | -7.0 | -7.4 | -3.9 | -7.6 | -6.6 | -7.9 | -8.7 | -9.7 | **-7.5** | **-9.2** | **-7.0** | **7.6** |
| AC(20-15°S) | -5.1 | -4.7 | -5.0 | -4.8 | -5.3 | -5.5 | -5.6 | -6.0 | -5.8 | -5.7 | -4.1 | -5.3 | -5.2 | -4.3 | -3.6 | -5.8 | -3.6 | -4.1 | -3.6 | -4.0 | -5.0 | -3.1 | -4.8 | -4.9 | -6.1 | **-5.0** | **-5.5** | **-4.1** | **4.4** |
| EACC(10-5°S) | 8.2 | 8.6 | 5.9 | 5.8 | 7.4 | 6.9 | 7.0 | 11.3 | 7.0 | 6.9 | 10.1 | 6.1 | 5.0 | 8.3 | 7.2 | 8.8 | 6.3 | 5.7 | 9.6 | 9.7 | 5.9 | 4.8 | 6.3 | 6.8 | 8.3 | **7.0** | **6.1** | **8.3** | **-8.1** |
| MNC(14-11°S) | 3.6 | 4.2 | 3.3 | 3.9 | 2.6 | 2.0 | 1.7 | 6.1 | 2.8 | 2.7 | 6.8 | 2.6 | 2.6 | 4.1 | 3.7 | 3.8 | 4.5 | 3.0 | 5.8 | 8.3 | 1.5 | 1.0 | 3.0 | 3.3 | 4.1 | **3.3** | **2.6** | **4.1** | **-4.6** |
| SMC(28-23°S) | -1.6 | -2.6 | -1.5 | -1.1 | -0.9 | -0.7 | -0.4 | -1.4 | -0.1 | -0.2 | -1.8 | -1.0 | -1.3 | -0.4 | -1.7 | -0.5 | -1.2 | -0.6 | -2.6 | -2.1 | 0.0 | -1.7 | -1.0 | -1.1 | -0.6 | **-1.1** | **-1.6** | **-0.6** | **1.6** |
| ATL18(18-22°N) | 2.6 | 3.4 | 5.4 | 4.3 | 5.0 | 6.0 | 4.6 | 6.6 | 8.0 | 6.4 | 5.5 | 8.3 | 3.5 | 6.4 | 5.8 | 8.5 | 4.4 | 4.7 | 6.9 | 4.6 | 4.7 | 4.7 | 5.5 | 4.8 | 6.7 | **5.4** | **4.6** | **6.4** | **-6.0** |
| ATL25(25-30°N) | 7.5 | 6.9 | 8.0 | 6.0 | 7.5 | 3.7 | 7.4 | 5.0 | 7.9 | 7.5 | 6.7 | 2.3 | 8.4 | 5.7 | 6.8 | 6.8 | 5.8 | 5.1 | 6.5 | 5.9 | 7.3 | 4.4 | 8.1 | 8.7 | 5.0 | **6.8** | **5.7** | **7.5** | **-7.7** |

*Table S9 0-100m integrated projected transport change (ssp126) for currents described in the text (CMIP6 models) . Median change is significant at 95% level using a two-sided Wilcoxon signed rank test unless in parenthesis*

| SSP126 change | ACCESS-CM2 | ACCESS-ESM1-5 | BCC-CSM2-MR | CAMS-CSM1-0 | CESM2-WACCM | CESM2 | CIESM | CMCC-CM2-SR5 | CNRM-CM6-1 | CNRM-ESM2-1 | CanESM5 | EC-Earth3-Veg | GISS-E2-1-G | INM-CM4-8 | INM-CM5-0 | IPSL-CM6A-LR | MIROC-ES2L | MIROC6 | MPI-ESM1-2-HR | MPI-ESM1-2-[illegible] | MRI-ESM2-0 | NESM3 | NorESM2-LM | NorESM2-MM | UKESM1-0-LL | **Median** | **25th percentile** | **75th percentile** | |
|---|---|---|---|---|---|---|---|---|---|---|---|---|---|---|---|---|---|---|---|---|---|---|---|---|---|---|---|---|---|
| ITF | 0.1 | -0.1 | 0.3 | 0.0 | 0.8 | 0.6 | 0.6 | 0.1 | 0.6 | 0.6 | -0.2 | 0.3 | 0.2 | -0.3 | -0.2 | 0.3 | 0.1 | 0.1 | 0.2 | 0.0 | 0.6 | -0.1 | 0.4 | 0.2 | 0.4 | **0.2** | **0.0** | **0.4** | |
| ACx(25°E) | -0.8 | -0.5 | -0.7 | -0.5 | 0.6 | 0.5 | 0.7 | -1.2 | -0.7 | -0.9 | -0.8 | -0.3 | -1.1 | -0.7 | 0.1 | -0.5 | -1.3 | -0.9 | -0.1 | -0.4 | 0.9 | -0.5 | -0.7 | -0.8 | -0.9 | **-0.7** | **-0.8** | **-0.3** | |
| KC(140°E) | 0.5 | 1.2 | 0.5 | 0.2 | 0.3 | 0.3 | 0.5 | 0.9 | -0.2 | -0.2 | 0.5 | 0.4 | 0.0 | 1.3 | 0.3 | 0.2 | 0.5 | -0.1 | 0.4 | 0.6 | 0.0 | 0.5 | -0.2 | -0.1 | 0.4 | **0.4** | **0.0** | **0.5** | |
| KC(150°E) | 0.6 | 0.9 | 0.5 | 0.3 | 0.5 | 0.4 | 0.6 | 0.9 | 0.2 | 0.2 | 0.9 | 0.8 | 0.4 | 0.5 | 0.1 | 0.5 | 0.8 | 0.3 | 1.0 | 1.0 | 0.2 | 0.8 | 0.0 | 0.2 | 0.7 | **0.5** | **0.3** | **0.8** | |
| GS(300°E) | -0.8 | -0.9 | -0.6 | -0.1 | -2.3 | -2.2 | -3.6 | -1.1 | -1.5 | -1.3 | -1.0 | -0.7 | -3.1 | -0.6 | -0.1 | -0.4 | -0.7 | -1.3 | -1.3 | -1.0 | -4.1 | -0.4 | -2.9 | -2.5 | -1.2 | **-1.1** | **-2.2** | **-0.7** | |
| TASL(146°E) | -0.4 | -0.2 | -0.3 | -0.1 | -0.2 | -0.3 | -0.2 | 0.0 | -0.3 | -0.6 | -0.6 | -0.2 | -0.3 | 0.3 | -0.5 | -0.6 | -0.5 | -0.3 | -0.2 | -1.1 | -0.2 | 0.0 | -0.2 | -0.3 | -0.6 | **-0.3** | **-0.5** | **-0.2** | |
| EAC(30-25°S) | 0.2 | -0.1 | 0.2 | 0.3 | -0.3 | -0.3 | -0.5 | -0.2 | -0.1 | 0.0 | 0.2 | -0.1 | -0.1 | 0.1 | 0.4 | 0.2 | 0.0 | 0.2 | -0.1 | 0.1 | -0.2 | -0.4 | 0.4 | 0.4 | -0.1 | **(0.0)** | **-0.1** | **0.2** | |
| EACx(40-35°S) | -0.4 | -0.2 | -0.1 | -0.1 | -0.3 | -0.3 | -0.3 | -0.5 | -0.1 | -0.2 | -0.3 | -0.1 | -0.5 | -0.3 | -0.5 | -0.2 | -0.6 | -0.2 | -0.5 | -0.3 | -0.2 | -0.2 | -0.2 | -0.2 | -0.4 | **-0.3** | **-0.4** | **-0.2** | |
| MC(7-10°N) | -0.1 | -1.4 | -0.4 | -0.2 | 0.1 | -0.1 | -1.4 | 0.2 | -0.2 | -0.1 | -0.6 | 0.0 | -0.4 | -0.2 | -0.3 | 0.1 | 0.2 | 0.7 | -0.5 | -0.2 | -0.3 | 0.0 | -1.0 | -0.3 | 0.4 | **-0.2** | **-0.4** | **0.0** | |
| KC(20-25°N) | 0.2 | 0.4 | 0.2 | 0.0 | 0.1 | 0.3 | 0.6 | 0.2 | -0.2 | -0.2 | 0.2 | 0.1 | -0.1 | 0.0 | 0.0 | 0.2 | 0.2 | -0.3 | 0.4 | 0.4 | 0.0 | -0.1 | -0.1 | -0.1 | 0.0 | **(0.1)** | **-0.1** | **0.2** | |
| KC(25-30°N) | 0.3 | 0.6 | 0.2 | 0.1 | 0.5 | 0.6 | 0.4 | 1.0 | -0.1 | 0.0 | 0.6 | 0.5 | -0.1 | -0.3 | -0.1 | 0.2 | 0.5 | -0.3 | 0.5 | 0.3 | 0.0 | 0.4 | -0.1 | 0.0 | 0.4 | **0.3** | **0.0** | **0.5** | |
| KC(35-40°S) | 0.3 | 0.0 | 0.4 | 0.4 | 0.4 | 0.1 | -0.2 | 0.4 | 0.2 | 0.3 | 0.4 | -0.2 | 0.8 | 0.9 | 0.6 | 0.1 | 0.0 | 0.3 | 0.2 | 0.3 | 0.0 | 0.3 | 0.6 | 0.6 | 0.0 | **0.3** | **0.1** | **0.4** | |
| OC(50-55°N) | -0.2 | -0.1 | 0.1 | -0.1 | -0.3 | -0.2 | -0.6 | -0.1 | -0.4 | -0.4 | -0.4 | -0.2 | -0.1 | -0.1 | 0.1 | -0.2 | 0.2 | -0.2 | -0.2 | -0.4 | -0.6 | -0.5 | -0.1 | -0.2 | -0.6 | **-0.2** | **-0.4** | **-0.1** | |
| GPC(15-13°S) | 0.9 | 0.8 | 0.1 | 0.8 | 1.1 | 0.8 | 1.6 | -0.3 | 0.4 | 0.6 | -0.4 | 0.8 | 1.2 | -0.6 | -0.4 | 0.1 | 0.3 | 0.1 | 0.5 | 0.2 | 0.5 | 0.4 | -1.1 | 0.3 | 0.4 | **0.4** | **0.1** | **0.8** | |
| NZ(38-34°S) | 0.7 | -0.1 | 0.2 | 0.2 | 0.1 | 0.2 | 0.3 | -0.1 | -0.1 | 0.0 | 0.1 | -0.2 | 0.5 | 0.0 | 0.8 | 0.1 | 0.3 | 0.3 | 0.3 | 0.4 | 0.3 | 0.0 | 0.3 | 0.1 | 0.3 | **0.2** | **0.0** | **0.3** | |
| NZ(50-46°S) | 0.0 | 0.0 | 0.1 | 0.0 | -0.1 | -0.2 | -0.4 | 0.1 | 0.3 | 0.3 | 0.0 | 0.3 | -0.1 | 0.6 | 0.0 | 0.1 | 0.7 | -0.1 | -0.6 | 0.0 | -0.1 | 0.3 | -0.1 | -0.1 | 0.1 | **(0.0)** | **-0.1** | **0.1** | |
| NGC(10-5°S) | 1.5 | 1.0 | 0.5 | 1.6 | 2.7 | 2.2 | 2.0 | 0.6 | 0.9 | 1.4 | -0.2 | 1.3 | 2.1 | 0.6 | 0.7 | 0.3 | 1.9 | 1.2 | 1.1 | 0.9 | 1.2 | 1.2 | 0.4 | 0.6 | 1.6 | **1.2** | **0.6** | **1.6** | |
| BC(40-35°S) | -0.5 | -0.3 | -0.5 | -0.2 | -0.9 | -0.7 | -1.0 | -0.9 | -0.5 | -0.3 | -0.5 | -0.3 | -0.6 | -1.1 | -0.2 | -0.3 | -0.6 | -0.4 | -0.6 | -0.6 | -0.8 | -0.1 | -0.6 | -0.7 | 0.1 | **-0.5** | **-0.7** | **-0.3** | |
| NBC(10-5°S) | -0.3 | 0.0 | 0.0 | 0.2 | -0.2 | -0.2 | -0.4 | -0.8 | -0.5 | -0.5 | -0.7 | 0.0 | -1.5 | -0.3 | 0.1 | -0.7 | -0.2 | -0.4 | 0.0 | -0.1 | -1.0 | 0.4 | -1.0 | -0.4 | -0.5 | **-0.3** | **-0.5** | **0.0** | |
| GS(28-33°N) | -0.4 | -0.1 | -0.1 | 0.1 | -1.5 | -1.4 | -2.4 | -1.0 | -1.1 | -1.1 | -0.9 | -0.4 | -1.8 | -0.5 | -0.7 | -0.6 | -0.7 | -1.0 | -1.2 | -1.3 | -3.7 | -0.4 | -2.3 | -1.9 | -0.9 | **-1.0** | **-1.4** | **-0.5** | |
| LAB(52-5°N) | 0.4 | -0.2 | -1.3 | -0.1 | 0.7 | 1.4 | 0.0 | 0.0 | 0.0 | -0.1 | -0.1 | -0.3 | 1.0 | 0.2 | 0.1 | 0.0 | -0.1 | 0.0 | -0.8 | 0.0 | 0.0 | 0.2 | 0.4 | 0.8 | 0.2 | **(0.0)** | **-0.1** | **0.2** | |
| AC(34-30°S) | 0.4 | 0.4 | 0.2 | 0.1 | 0.0 | 0.0 | 0.0 | 0.8 | 0.3 | 0.2 | 0.6 | 0.3 | 0.7 | 0.5 | 0.0 | 0.7 | 0.7 | 1.0 | 0.4 | 0.1 | 1.9 | 0.3 | 1.2 | 1.6 | 0.7 | **0.4** | **0.2** | **0.7** | |
| AC(20-15°S) | 0.0 | -0.4 | 0.2 | 0.1 | 0.3 | 0.2 | 0.4 | 0.1 | 0.5 | 0.4 | -0.2 | 0.1 | 0.1 | -0.2 | -0.2 | 0.5 | 0.3 | 0.2 | 0.1 | 0.3 | 0.5 | 0.1 | 0.3 | 0.2 | 0.3 | **0.2** | **0.1** | **0.3** | |
| EACC(10-5°S) | 0.3 | -0.2 | -0.3 | -0.3 | -0.3 | -0.2 | -0.9 | -0.5 | 0.4 | 0.2 | -0.6 | -0.1 | 0.1 | -0.3 | -0.1 | 0.0 | 0.3 | 0.2 | 0.3 | 0.4 | -0.6 | 0.2 | 0.4 | 0.4 | -0.1 | **(-0.1)** | **-0.3** | **0.3** | |
| MNC(14-11°S) | -0.4 | -1.1 | -0.2 | -0.1 | -0.4 | -0.2 | -0.6 | -1.0 | -0.1 | 0.1 | -0.9 | -0.5 | -0.2 | 0.0 | -0.2 | -0.5 | -0.4 | -0.2 | -0.4 | -0.7 | -0.2 | 0.0 | -0.2 | -0.7 | -1.0 | **-0.4** | **-0.6** | **-0.2** | |
| SMC(28-23°S) | 0.0 | 0.5 | -0.1 | -0.3 | 0.1 | 0.1 | 0.0 | 0.1 | -0.1 | 0.0 | 0.0 | 0.2 | 0.2 | -0.1 | 0.1 | 0.0 | 0.1 | 0.0 | -0.1 | 0.2 | -0.2 | 0.2 | 0.0 | 0.2 | 0.1 | **(0.0)** | **0.0** | **0.1** | |
| ATL18(18-22°N) | -0.1 | -0.3 | -0.2 | -0.1 | -0.4 | -0.6 | -0.2 | -0.6 | -1.6 | -1.3 | -0.1 | -0.5 | -0.1 | -0.6 | -0.2 | -0.6 | -0.1 | -0.3 | -0.2 | 0.1 | -0.4 | -0.2 | -0.4 | -0.2 | -0.2 | **-0.2** | **-0.5** | **-0.2** | |
| ATL25(25-30°N) | -0.4 | -0.1 | -0.1 | 0.1 | -1.6 | -1.6 | -2.6 | -0.7 | -1.3 | -1.1 | -0.7 | -0.1 | -1.7 | -0.7 | -0.8 | -0.7 | -0.6 | -0.9 | -1.2 | -0.8 | -3.4 | -0.4 | -2.3 | -2.1 | -0.6 | **-0.8** | **-1.6** | **-0.6** | |

*Table S10 0-100m integrated projected transport change (ssp585) for currents described in the text (CMIP6 models) . Median change is significant at 95% level using a two-sided Wilcoxon signed rank test unless in parenthesis*

| SSP585 change | ACCESS-CM2 | ACCESS-ESM1-5 | BCC-CSM2-MR | CAMS-CSM1-0 | CESM2-WACCM | CESM2 | CIESM | CMCC-CM2-SR5 | CNRM-CM6-1 | CNRM-ESM2-1 | CanESM5 | EC-Earth3-Veg | GISS-E2-1-G | INM-CM4-8 | INM-CM5-0 | IPSL-CM6A-LR | MIROC-ES2L | MIROC6 | MPI-ESM1-2-HR | MPI-ESM1-2-LR | MRI-ESM2-0 | NESM3 | NorESM2-LM | NorESM2-MM | UKESM1-0-LL | **Median** | **25th percentile** | **75th percentile** | |
|---|---|---|---|---|---|---|---|---|---|---|---|---|---|---|---|---|---|---|---|---|---|---|---|---|---|---|---|---|
| ITF | 0.3 | 0.1 | 0.4 | 0.0 | 0.8 | 0.9 | 0.5 | 0.2 | 0.9 | 0.8 | 1.0 | 0.5 | 0.3 | -0.4 | -0.2 | 0.7 | 0.1 | 0.4 | 0.3 | 0.0 | 1.1 | -0.4 | 0.4 | 0.3 | 0.7 | **0.4** | **0.1** | **0.7** | |
| ACx(25°E) | -1.2 | -0.8 | -1.4 | -1.0 | 0.3 | 0.3 | 0.5 | -1.5 | -1.3 | -1.3 | -0.5 | -0.6 | -1.4 | -0.9 | 0.1 | -0.9 | -1.7 | -1.2 | -0.2 | -0.7 | 0.7 | -0.6 | -1.2 | -1.4 | -1.6 | **-0.9** | **-1.3** | **-0.5** | |
| KC(140°E) | 0.3 | 1.3 | 0.9 | 0.2 | 0.3 | 0.1 | 0.7 | 0.6 | -0.6 | -0.5 | -1.2 | 0.0 | 0.1 | 1.3 | 0.3 | 0.2 | 0.4 | 0.1 | 0.1 | 0.4 | -0.5 | 0.1 | -0.5 | -0.3 | 0.3 | **(0.2)** | **0.0** | **0.4** | |
| KC(150°E) | 0.4 | 1.0 | 0.9 | 0.3 | 0.4 | 0.4 | 1.0 | 0.8 | -0.1 | 0.2 | -2.7 | 0.6 | 0.3 | 0.3 | 0.4 | 0.8 | 0.8 | 0.5 | 0.9 | 0.7 | 0.5 | 0.4 | -0.3 | 0.1 | 0.7 | **0.4** | **0.3** | **0.8** | |
| GS(300°E) | -1.2 | -0.9 | -1.4 | -0.2 | -2.4 | -2.4 | -3.5 | -1.3 | -1.7 | -1.3 | -4.1 | -1.0 | -3.6 | -0.9 | -0.6 | -0.6 | -0.8 | -1.6 | -1.9 | -1.6 | -4.5 | -0.3 | -3.3 | -3.3 | -1.7 | **-1.6** | **-2.4** | **-0.9** | |
| TASL(146°E) | -0.7 | -0.4 | -0.4 | -0.2 | -0.4 | -0.5 | -0.5 | -0.2 | -0.8 | -0.9 | -2.3 | -0.7 | -0.4 | -0.2 | -0.5 | -1.2 | -1.1 | -0.9 | -0.5 | -1.2 | -1.4 | 0.0 | -0.5 | -0.5 | -1.2 | **-0.5** | **-0.9** | **-0.4** | |
| EAC(30-25°S) | 0.3 | -0.1 | 0.3 | 0.4 | -1.7 | -0.5 | -0.8 | -0.3 | -0.2 | -0.2 | -0.7 | 0.0 | 0.0 | 0.1 | 0.4 | 0.2 | -0.1 | 0.1 | -0.2 | 0.1 | -0.2 | -0.4 | 0.4 | 0.5 | 0.1 | **(0.0)** | **-0.2** | **0.2** | |
| EACx(40-35°S) | -0.8 | -0.5 | -0.3 | -0.2 | -1.6 | -0.7 | -0.5 | -0.8 | -0.6 | -0.6 | -2.3 | -0.5 | -0.9 | -0.5 | -0.6 | -0.5 | -1.0 | -0.5 | -0.5 | -0.5 | -1.1 | -0.3 | -0.5 | -0.3 | -1.1 | **-0.5** | **-0.8** | **-0.5** | |
| MC(7-10°N) | -0.3 | -1.7 | -0.6 | -0.5 | -0.8 | 0.0 | -1.8 | 0.2 | 0.1 | -0.1 | 0.2 | 0.1 | -0.5 | 0.0 | -0.1 | 0.1 | 0.1 | 0.5 | -0.6 | -0.2 | -0.3 | 0.1 | -0.9 | -0.2 | 0.5 | **-0.1** | **-0.5** | **0.1** | |
| KC(20-25°N) | 0.0 | 0.4 | 0.3 | 0.0 | 0.8 | 0.2 | 1.0 | 0.1 | -0.3 | -0.5 | -0.3 | -0.2 | -0.1 | -0.1 | 0.1 | 0.2 | 0.2 | 0.1 | 0.1 | 0.3 | -0.1 | -0.2 | -0.5 | -0.2 | -0.3 | **(0.0)** | **-0.2** | **0.2** | |
| KC(25-30°N) | 0.2 | 0.7 | 0.4 | 0.0 | 1.5 | 0.5 | 0.2 | 1.0 | -0.3 | -0.2 | 0.2 | 0.2 | -0.1 | -0.8 | -0.1 | 0.3 | 0.5 | 0.0 | 0.4 | 0.3 | 0.2 | 0.3 | -0.5 | -0.2 | 0.2 | **0.2** | **-0.1** | **0.4** | |
| KC(35-40°S) | 0.7 | 0.1 | 0.3 | 0.7 | 1.2 | 0.3 | 0.0 | 0.6 | 0.3 | 0.4 | 1.8 | 0.3 | 1.2 | 1.5 | 0.9 | 0.2 | 0.3 | 0.7 | 0.6 | 0.7 | 1.2 | 0.6 | 1.1 | 1.0 | 0.2 | **0.6** | **0.3** | **1.0** | |
| OC(50-55°N) | -0.3 | -0.2 | -0.1 | 0.0 | -0.6 | -0.2 | -0.7 | -0.1 | -0.4 | -0.5 | -1.0 | -0.2 | 0.1 | -0.1 | 0.3 | -0.5 | -0.1 | -0.3 | -0.4 | -0.4 | -1.1 | -0.5 | 0.1 | 0.0 | -0.8 | **-0.3** | **-0.5** | **-0.1** | |
| GPC(15-13°S) | 1.0 | 0.9 | 0.3 | 1.1 | 1.0 | 1.0 | 1.4 | -0.6 | 1.2 | 1.3 | -1.8 | 1.0 | 1.9 | -0.9 | -0.5 | 0.5 | 0.5 | 0.3 | 0.8 | 0.6 | 1.0 | 0.5 | -0.7 | 0.3 | 0.9 | **0.8** | **0.3** | **1.0** | |
| NZ(38-34°S) | 1.1 | 0.1 | 0.4 | 0.2 | 0.1 | 0.4 | 0.3 | -0.1 | 0.0 | -0.1 | 1.1 | -0.1 | 0.7 | 0.2 | 0.9 | 0.3 | 0.4 | 0.5 | 0.4 | 0.4 | 1.2 | -0.1 | 0.3 | 0.2 | 0.7 | **0.3** | **0.1** | **0.5** | |
| NZ(50-46°S) | -0.1 | 0.0 | 0.1 | 0.0 | -1.3 | -0.4 | -0.5 | 0.1 | 0.4 | 0.6 | -2.2 | 0.4 | -0.1 | 0.4 | 0.1 | 0.1 | 1.0 | -0.2 | -0.8 | 0.0 | -0.5 | 0.5 | -0.2 | -0.2 | 0.1 | **(0.0)** | **-0.2** | **0.1** | |
| NGC(10-5°S) | 2.0 | 2.3 | 0.3 | 2.2 | 8.2 | 3.8 | 2.2 | 1.1 | 2.6 | 2.5 | 4.3 | 2.5 | 2.9 | 1.4 | 1.1 | 1.3 | 3.2 | 2.0 | 2.1 | 1.6 | 4.1 | 1.8 | 1.2 | 1.4 | 2.0 | **2.1** | **1.4** | **2.6** | |
| BC(40-35°S) | -0.7 | -0.6 | -0.9 | -0.4 | -1.9 | -1.0 | -1.3 | -0.8 | -0.7 | -0.5 | -1.2 | -0.6 | -0.7 | -1.1 | -0.1 | -0.5 | -0.7 | -0.5 | -0.9 | -0.9 | -1.9 | -0.3 | -0.8 | -0.8 | 0.1 | **-0.7** | **-0.9** | **-0.5** | |
| NBC(10-5°S) | 0.2 | 0.4 | -0.1 | 0.2 | 0.1 | 0.1 | 0.2 | -0.3 | -0.2 | -0.1 | -1.6 | 0.5 | -1.5 | -0.2 | 0.2 | -0.8 | 0.2 | -0.3 | 0.4 | 0.6 | -2.1 | 1.4 | -0.9 | -0.2 | 0.2 | **(0.1)** | **-0.3** | **0.2** | |
| GS(28-33°N) | -0.9 | -0.2 | -0.6 | 0.0 | -4.2 | -1.5 | -2.6 | -1.2 | -1.0 | -1.2 | -4.2 | -0.8 | -2.7 | -0.8 | -1.3 | -1.0 | -0.6 | -1.1 | -1.9 | -2.0 | -4.3 | -0.7 | -2.5 | -2.4 | -1.3 | **-1.2** | **-2.4** | **-0.8** | |
| LAB(52-5°N) | -0.5 | -0.4 | -1.0 | 0.0 | 1.2 | 1.2 | -0.1 | -0.2 | -0.1 | -0.3 | 1.4 | -0.3 | 0.9 | 0.4 | -0.3 | 0.1 | -0.2 | 0.0 | -0.8 | 0.0 | -0.4 | 0.2 | 0.4 | 0.8 | 0.4 | **(0.0)** | **-0.3** | **0.4** | |
| AC(34-30°S) | 0.5 | 0.3 | 0.3 | 0.4 | -0.3 | -0.2 | -0.2 | 0.6 | 0.2 | -0.1 | 0.6 | 0.4 | 0.8 | 0.4 | 0.0 | 0.8 | 0.8 | 1.2 | 0.4 | 0.0 | 1.9 | -0.1 | 1.2 | 1.5 | 0.7 | **0.4** | **0.0** | **0.8** | |
| AC(20-15°S) | 0.1 | -0.3 | 0.3 | 0.1 | -0.5 | 0.2 | 0.1 | 0.1 | 0.7 | 0.4 | -1.1 | 0.2 | 0.1 | -0.5 | -0.3 | 0.7 | 0.4 | 0.2 | 0.1 | 0.4 | 0.4 | 0.1 | 0.3 | 0.2 | 0.5 | **(0.2)** | **0.1** | **0.4** | |
| EACC(10-5°S) | -0.4 | -0.6 | -0.4 | -0.4 | -2.4 | -0.2 | -1.8 | -0.9 | 0.2 | 0.7 | -4.7 | -0.2 | -0.5 | -0.6 | -0.1 | -0.4 | 0.3 | 0.2 | 0.3 | 0.5 | -1.8 | 0.5 | 0.2 | 0.2 | -0.8 | **-0.4** | **-0.6** | **0.2** | |
| MNC(14-11°S) | -0.7 | -1.5 | -0.3 | -0.2 | -1.6 | -0.2 | -1.0 | -1.3 | -0.2 | 0.3 | -3.8 | -0.6 | -0.5 | -0.1 | -0.3 | -0.9 | -0.5 | -0.2 | -0.6 | -0.8 | -0.9 | 0.1 | -0.2 | -0.7 | -1.1 | **-0.6** | **-0.9** | **-0.2** | |
| SMC(28-23°S) | 0.1 | 0.6 | -0.2 | -0.5 | 0.8 | 0.1 | 0.1 | 0.2 | -0.3 | -0.1 | 1.4 | 0.2 | 0.3 | 0.1 | -0.1 | -0.1 | 0.1 | -0.1 | -0.2 | 0.0 | 0.2 | -0.1 | 0.1 | 0.1 | 0.1 | **(0.1)** | **-0.1** | **0.2** | |
| ATL18(18-22°N) | -0.1 | -0.4 | -0.5 | -0.2 | -0.8 | -0.5 | -0.3 | -0.5 | -1.8 | -1.4 | -0.1 | -0.9 | -0.1 | -0.7 | -0.4 | -0.8 | -0.2 | -0.3 | -0.5 | 0.2 | -0.6 | -0.4 | -0.6 | -0.4 | -0.2 | **-0.4** | **-0.6** | **-0.2** | |
| ATL25(25-30°N) | -1.1 | -0.2 | -0.7 | 0.0 | -3.8 | -1.7 | -2.5 | -0.9 | -1.3 | -1.2 | -3.2 | -0.2 | -2.5 | -1.1 | -1.4 | -1.0 | -0.5 | -1.0 | -1.8 | -1.3 | -3.8 | -0.7 | -2.4 | -2.7 | -0.9 | **(-1.2)** | **-2.4** | **-0.9** | |

*Table S 11 Transports derived from Ocean Reanalysis.*

| | 100m | | | | 1000m | | | |
|---|---|---|---|---|---|---|---|---|
| | ORAS5 | CCOR | GODAS | **Mean** | ORAS5 | CCOR | GODAS | **Mean** |
| ITF | -17.4 | -17.9 | -8.7 | **-14.7** | -6.4 | -6.6 | -5.6 | **6.2** |
| ACx(25°E) | -49.2 | -43.5 | -36.5 | **-43.1** | -10.9 | -10.7 | -5.0 | **8.9** |
| KC(140°E) | 36.0 | 33.4 | 38.6 | **36.0** | 8.1 | 8.1 | 8.5 | **-8.2** |
| KC(150°E) | 46.5 | 46.7 | 39.7 | **44.3** | 10.4 | 10.5 | 10.3 | **-10.4** |
| GS(300°E) | 45.3 | 37.8 | 37.5 | **40.2** | 8.4 | 7.9 | 9.1 | **-8.5** |
| TASL(146oE) | -5.4 | -10.0 | -0.6 | **-5.3** | -0.4 | -1.2 | 0.2 | **0.5** |
| EAC(30-25°S) | -7.1 | -10.7 | -17.3 | **-11.7** | -0.2 | -1.4 | -2.9 | **1.5** |
| EACx(40-35°S) | -7.1 | -7.7 | -1.8 | **-5.5** | -1.3 | -1.4 | -0.4 | **1.1** |
| MC(7-10°N) | -17.3 | -14.6 | -21.8 | **-17.9** | -7.7 | -7.2 | -11.1 | **8.6** |
| KC(20-25°N) | 17.8 | 24.7 | 23.6 | **22.0** | 4.0 | 6.6 | 5.7 | **-5.4** |
| KC(25-30°N) | 39.1 | 38.6 | 38.7 | **38.8** | 8.0 | 7.3 | 6.7 | **-7.3** |
| KC(35-40°S) | 7.9 | 9.6 | 6.7 | **8.1** | 1.8 | 1.6 | 1.3 | **-1.6** |
| OC(50-55°N) | -10.6 | -11.5 | -16.2 | **-12.8** | -2.2 | -2.3 | -2.3 | **2.3** |
| GPC(15-13°S) | 13.1 | 10.1 | 13.3 | **12.2** | 0.2 | 0.3 | 0.5 | **-0.3** |
| NZECC(38-34°S) | -4.6 | 0.3 | -9.1 | **-4.4** | 0.4 | 0.8 | -0.4 | **-0.3** |
| NZSC(50-46°S) | 18.9 | 14.6 | 15.1 | **16.2** | 3.3 | 2.5 | 2.7 | **-2.8** |
| NGC(10-5°S) | 6.0 | 8.6 | 20.8 | **11.8** | -2.5 | -3.1 | -1.1 | **2.2** |
| BC(40-35°S) | -32.5 | -14.3 | -8.7 | **-18.5** | -4.5 | -1.7 | -0.8 | **2.3** |
| NBC(10-5°S) | 11.5 | 16.1 | 18.1 | **15.2** | 2.3 | 3.6 | 2.5 | **-2.8** |
| GS(28-33°N) | 45.9 | 46.7 | 37.2 | **43.3** | 9.1 | 8.2 | 5.8 | **-7.7** |
| LAB(52-5°N) | 2.4 | -9.3 | -11.9 | **-6.3** | 1.0 | -1.2 | -1.3 | **0.5** |
| AC(34-30°S) | -53.7 | -53.7 | -47.3 | **-51.6** | -8.1 | -8.5 | -6.3 | **7.6** |
| AC(20-15°S) | -21.1 | -16.5 | -11.8 | **-16.4** | -4.9 | -5.1 | -3.1 | **4.4** |
| EACC(10-5°S) | 7.4 | 10.5 | 23.0 | **13.6** | 6.7 | 7.2 | 10.4 | **-8.1** |
| MNC(14-11°S) | 21.5 | 14.6 | 21.2 | **19.1** | 4.9 | 3.8 | 5.1 | **-4.6** |
| SMC(28-23°S) | -10.9 | -15.6 | -13.0 | **-13.2** | -1.2 | -1.9 | -1.7 | **1.6** |
| ATLYC(18-22°N) | 25.7 | 21.9 | 8.4 | **18.6** | 7.2 | 6.8 | 4.0 | **-6.0** |
| ATL25(25-30°N) | 46.1 | 48.2 | 32.5 | **42.3** | 8.7 | 8.4 | 6.0 | **-7.7** |

*Table S12 Observational transport estimates from literature.*

| Description | Latitude | Transport (Sv) | Reference |
|---|---|---|---|
| Kuroshio Current (KC) | ~28ºN | Core transport minimum in Autumn | [2] |
| | KC extension + southern recirculation | Surface transport maximum in summer/autumn, minimum in winter/spring | [3] |
| | ~24oN | Maximum in summer (July) minimum in winter (December) | |
| | ~26°N | 21.3 | [4] |
| | 24°N | 21.5 | [5] |
| | 18°N | 15 | [6] |
| | 18°N | 14 | [7] |
| | 12°N | −16 | [8] |
| Mindanao Current (MC) | 8.5°N | −36.1 | [9] |
| | 8°N | −27 | [7] |
| | 7.5°N | −18.2 | [9] |
| | 7.5°N | −28 | [6] |
| | 7°N | −26 | [8] |
| | 5°N | −25 | [10] |
| | | 10 | [11] |
| Indonesian Throughflow (ITF) | | 13 | [12] |
| | | 16 | [13] |
| | | 15 (strongest in Jul [17.9 Sv], weakest in Feb) | [14] |
| | ~1°S | 23.8 | [15] |
| New Guinea Coastal Undercurrent (NGCU) | 2.5°S | 21.4 (stronger in boreal summer, weaker in winter) | [16] |
| | ~12°S | 29 | [17] |
| | | (stronger in winter [13.2 Sv], weaker in summer [7.5 Sv]) | [18] |
| Gulf of Papua Current (GPC) | ~11°S | 24 | [19] |
| | 27°S | −29 | [20] |
| East Australian Current (EAC) | 27°S | 22.1 | [21] |
| | 27°S | −19.5 | [22] |
| | 29°S | −25.2 | [20] |

| | | | |
|---|---|---|---|
| | 30°S | −22.1 | 23 |
| | 33°S | −37 | 20 |
| | 28°S | 36.3 Sv<br>27.4 Sv<br>(*strongest in summer weakest in winter)* | 24 |
| | 43°S | 6.3-9.2 | 25 |
| EAC extension | 43°S | ~−7.4 | 20 |
| | 44°S | 7.1 | 24 |
| | | 8 +/-13 Sv | 26 |
| Tasman Leakage (TASL) | ~32°S | -22 | 27 |
| East Cape Current (ECC) | ~40°S | -13.4 | 28 |
| | | −11.2 to -11.8 | 20 |
| East Auckland Current (EAUC) | ~34°S | 11-34 | 27 |
| | | −19.3 | 20 |
| | | 9 | 29 |
| | | 9.5 | 30 |
| | 31.75°S | 8 | 31 |
| | 33°S | 15 | 31 |
| | | 8 | 28 |
| | | 8.3 (3.4-12.9) | 32 |
| Southland Current (SC) | | 10 | 28 |
| | 0° | 10 (21 in NH summer) | 33 |
| East African Coast Current (EACC) | ~1-2°S | 15* | 34 |
| | 4-5°S | 19.9 | 35 |
| | 12°S | 29.6* | 36 |
| North Madagascar Current (NMC) | 12°S | 26.9* (No observed seasonality) | 37 |
| | 12°S | 48* | 38 |
| | 23°S | −20.6 | 36 |
| South Madagascar | | −22 | 38 |
| | 23°S | −20.3* (No observed seasonality) | 37 |

| | | | |
|---|---|---|---|
| Current (SMC) | 23°S | −17.8 | 39 |
| | 25°S | −20* | 40 |
| | 25°S | −30* | 41 |
| | 17°S | −15* | 42 |
| Mozambique Channel (MZC) | 17°S | −16.7 (strongest in Sep and weakest in Mar) | 43 |
| | 24°S | −19* | 44 |
| | 25°S | −17.7* | 40 |
| | 31°S | −69.7 | 45 |
| Agulhas Current (AC) | 32°S | −76* | 40 |
| | 34°S | −77 (strongest in Mar-Apr, weakest in Aug) | 46 |
| | | 10 | 47 |
| Agulhas Leakage | | 15 | 48 |
| | | 2-10 | 49 |
| | | ~9 | 50 |
| | | 16 | 51 |
| | | 15 | 52 |
| | 2.5ºS | 18 (stronger Jul-Aug, weaker Apr-May) | 53 |
| North Brazil Current (NBC) | 4ºN | 26 | 54 |
| | 11ºS | 23 | 55 |
| | 11ºS | 26 | 55 |
| | 20ºS | -4 (stronger in summer, weaker in winter) | 56 |
| Brazil Current (BC) | 25ºS | -5.5 | 57 |
| | 28ºS | -16 | 58 |
| | 32ºS | -19 | 59 |
| | 36ºS | -23 | 60 |
| | 37ºS | -16 | 61 |
| | 38ºS | -20 | 60 |
| | 38ºS | -18 | 62 |
| | 38ºS | -19 | 63 |
| | 36ºS | -23 | 64 |

| | | | |
|---|---|---|---|
| | 25ºS | -3.8 | [65] |
| | 32ºS | -13.9 | [65] |
| | 22ºS | -4.6 | [66] |
| | 73ºW (~36ºN) | 87 (2000m)<br>(stronger in fall, weaker in spring) | [67] |
| Gulf Stream (GS) | 26.5oN | Maximum transport in July minimum in November | [68] |
| | 68ºW (~38ºN) | 113 (2000m) | [69] |
| | 55ºW | 93 | [70] |
| | 60ºW | 160 | [71] |
| | 27ºN | 32.4 | [72] |
| | 25ºN | 32 | [73] |
| | 23ºN | 25 | [74] |
| | 20ºN | 23.8 | [75] |
| Yucatan Channel | 20ºN | 23 | [76] |
| | 20ºN[2] | 30.0 (re-evaluation of past estimates) | [77] |
| | | | |

*Table S 13 Median transports and corresponding projected changes for CMIP5 and CMIP6 models. Bold numbers indicate differences between CMIP5 and CMIP6 are significant at the 95% level based on a 2-sided Wilcoxon rank sum test*

| | Historical Transport | | | Projected change | | |
|---|---|---|---|---|---|---|
| | CMIP5 MMM | CMIP6 MMM | Difference | CMIP5 MMM | CMIP6 MMM | Difference |
| ITF | -13.0 | -13.1 | -0.1 | 3.2 | 3.0 | -0.2 |
| ACx (25°E) | -29.0 | -50.3 | -21.3 | -9.7 | -6.0 | **3.8** |
| KC (140°E) | 45.0 | 42.2 | -2.8 | -2.6 | -2.3 | 0.3 |
| KC (150°E) | 46.2 | 44.9 | -1.3 | -2.0 | -1.6 | 0.3 |
| GS (300°E) | 37.4 | 34.8 | -2.6 | -9.4 | -6.9 | 2.6 |
| TASL (146°E) | 2.4 | -2.7 | **-5.1** | -5.7 | -4.6 | 1.0 |
| EAC (30-25°S) | -22.7 | -20.8 | 2.0 | 0.5 | 0.3 | -0.2 |
| EACx (40-35°S) | -1.7 | -4.6 | -3.0 | -5.6 | -5.3 | 0.2 |
| MC (7-10°N) | -24.5 | -23.5 | 1.0 | 3.3 | 3.5 | 0.1 |
| KC (20-25°N) | 28.1 | 25.1 | -3.0 | -2.6 | -3.0 | -0.4 |
| KC (25-30°N) | 40.4 | 36.7 | -3.8 | -1.4 | -1.7 | -0.3 |
| KC (35-40°S) | 12.7 | 12.5 | -0.1 | 2.0 | 2.2 | 0.2 |
| OC (50-55°N) | -8.5 | -10.2 | -1.7 | -0.9 | -0.6 | 0.3 |
| GPC (15-13°S) | 18.4 | 17.2 | -1.2 | 2.3 | 2.1 | -0.2 |
| NZ (38-34°S) | -16.6 | -13.8 | 2.8 | 2.5 | 3.2 | 0.6 |
| NZ (50-46°S) | 11.6 | 12.0 | 0.5 | 0.1 | -0.4 | -0.6 |
| NGC (10-5°S) | 21.9 | 19.5 | -2.4 | 3.1 | 2.9 | -0.2 |
| BC (40-35°S) | -14.5 | -18.6 | -4.1 | -6.4 | -5.2 | 1.3 |
| NBC (10-5°S) | 21.3 | 21.8 | 0.4 | -3.4 | -2.5 | 1.0 |
| GS (28-33°N) | 40.8 | 39.9 | -0.9 | -8.5 | -7.2 | 1.3 |
| LAB (52-5°N) | -9.2 | -8.3 | 0.9 | 2.0 | 0.8 | -1.2 |
| AC (34-30°S) | -59.0 | -59.3 | -0.3 | 5.2 | 5.0 | -0.2 |
| AC (20-15°S) | -19.3 | -21.9 | -2.6 | 4.6 | 4.5 | -0.1 |
| EACC (10-5°S) | 18.1 | 17.9 | -0.2 | -0.1 | -0.5 | -0.4 |
| MNC (14-11°S) | 23.7 | 20.7 | -3.0 | -3.1 | -2.9 | 0.2 |
| SMC (28-23°S) | -15.7 | -13.7 | **2.1** | 1.6 | 1.4 | -0.2 |
| ATL18 (18-22°N) | 16.5 | 17.7 | 1.3 | -2.3 | -3.2 | -0.9 |
| ATL25 (25-30°N) | 38.3 | 36.4 | -1.9 | -8.8 | -6.9 | 1.9 |

Supplementary Information References


1. Geophysical Data Center. 2-minute Gridded Global Relief Data (ETOPO2) v2. https://doi.org/10.7289/V5J1012Q (2006).
2. Wei, Y., Pei, Y. & Zhang, R.-H. Seasonal variability of the Kuroshio Current at the PN Section in the East China Sea based on in-situ observation from 1987 to 2010. *Acta Oceanol. Sin.* **34**, 12–21 (2015).
3. Yang, Y. & San Liang, X. On the Seasonal Eddy Variability in the Kuroshio Extension. *J. Phys. Oceanogr.* **48**, 1675–1689 (2018).
4. Bingxian, G. Analysis of the variations of volume transports of the Kuroshio in the East China Sea. *Chin. J. Oceanol. Limnol.* **1**, 156–165 (1983).
5. Johns, W. *et al.* The Kuroshio east of Taiwan: Moored transport Observations from the WOCE PCM-1 array. *J. Phys. Oceanogr. - J PHYS Ocean.* **31**, 1031–1053 (2001).
6. Ju, X., Ma, C., Xiong, X., Guo, Y. & Yu, L. Circulation and Heat Flux along the Western Boundary of the North Pacific. *J. Ocean Univ. China* (2019) doi:10.1007/s11802-020-4168-z.
7. Qu, T., Mitsudera, H. & Yamagata, T. On the western boundary currents in the Philippine Sea. *J. Geophys. Res. Oceans* **103**, 7537–7548 (1998).
8. Lukas, R. *et al.* Observations of the Mindanao Current during the Western Equatorial Pacific Ocean Circulation Study. *J. Geophys. Res.* **96**, 7089–7104 (1991).
9. Schönau, M. *et al.* The Mindanao Current: Mean Structure and Connectivity. *Oceanography* **28**, 34–45 (2015).
10. Wijffels, S., Firing, E. & Toole, J. The mean structure and variability of the Mindanao Current at 8°N. *J. Geophys. Res. Oceans* **100**, 18421–18435 (1995).
11. Godfrey, J. & Golding, T. The Sverdrup Relation in the Indian Ocean, and the Effect of Pacific-Indian Ocean Throughflow on Indian Ocean Circulation and on the East Australian Current. *J. Phys. Oceanogr.* **11**, 771–779 (1981).
12. Godfrey, J. S. A sverdrup model of the depth-integrated flow for the world ocean allowing for island circulations. *Geophys. Astrophys. Fluid Dyn.* **45**, 89–112 (1989).

13. Ganachaud, A. & Wunsch, C. Improved estimates of global ocean circulation, heat transport and mixing from hydrographic data. *Nature* **408**, 453–457 (2000).
14. Sprintall, J., Wijffels, S. E., Molcard, R. & Jaya, I. Direct estimates of the Indonesian Throughflow entering the Indian Ocean: 2004–2006. *J. Geophys. Res. Oceans* **114**, n/a-n/a (2009).
15. Gouriou, Y. & Toole, J. Mean circulation of the upper layers of the western equatorial Pacific Ocean. *J. Geophys. Res. Oceans* **98**, 22495–22520 (1993).
16. Ueki, I. Observation of current variations off the New Guinea coast including the 1997–1998 El Niño period and their relationship with Sverdrup transport. *J. Geophys. Res.* **108**, (2003).
17. Gasparin, F., Ganachaud, A., Maes, C., Marin, F. & Eldin, G. Oceanic transports through the Solomon Sea: The bend of the New Guinea Coastal Undercurrent. *Geophys. Res. Lett.* **39**, (2012).
18. Ridgway, K. R., Benthuysen, J. A. & Steinberg, C. Closing the Gap Between the Coral Sea and the Equator: Direct Observations of the North Australian Western Boundary Currents. *J. Geophys. Res. Oceans* **123**, 9212–9231 (2018).
19. Kessler, W. S. & Cravatte, S. Mean circulation of the Coral Sea. *J. Geophys. Res. Oceans* **118**, 6385–6410 (2013).
20. Ridgway, K. R. & Dunn, J. R. Mesoscale structure of the mean East Australian Current System and its relationship with topography. *Prog. Oceanogr.* **56**, 189–222 (2003).
21. Sloyan, B., Ridgway, K. & Cowley, R. The East Australian Current and Property Transport at 27 S from 2012-2013. *J. Phys. Oceanogr.* **46**, 160108151222003 (2016).
22. Zilberman, N. V., Roemmich, D. H., Gille, S. T. & Gilson, J. Estimating the Velocity and Transport of Western Boundary Current Systems: A Case Study of the East Australian Current near Brisbane. *J. Atmospheric Ocean. Technol.* **35**, 1313–1329 (2018).
23. Mata, M. M., Tomczak, M., Wijffels, S. & Church, J. A. East Australian Current volume transports at 30°S: Estimates from the World Ocean Circulation Experiment hydrographic sections PR11/P6 and the PCM3 current meter array. *J. Geophys. Res. Oceans* **105**, 28509–28526 (2000).
24. Ridgway, K. R. & Godfrey, J. S. Seasonal cycle of the East Australian Current. *J. Geophys. Res.-Oceans* **102**, 22921–22936 (1997).

25. Chiswell, S. M., Toole, J. & Church, J. Transports across the Tasman Sea from WOCE repeat sections: The East Australian Current 1990–94. *N. Z. J. Mar. Freshw. Res.* **31**, 469–475 (1997).
26. Rintoul, S. R. & Sokolov, S. Baroclinic transport variability of the Antarctic Circumpolar Current south of Australia (WOCE repeat section SR3). *J. Geophys. Res.-Oceans* **106**, 2815–2832 (2001).
27. Stanton, B. R., Sutton, P. J. H. & Chiswell, S. M. The East Auckland Current, 1994–95. *N. Z. J. Mar. Freshw. Res.* **31**, 537–549 (1997).
28. Fernandez, D., Bowen, M. & Sutton, P. Variability, coherence and forcing mechanisms in the New Zealand ocean boundary currents. *Prog. Oceanogr.* **165**, 168–188 (2018).
29. Roemmich, D. & Sutton, P. The mean and variability of ocean circulation past northern New Zealand: Determining the representativeness of hydrographic climatologies. *J. Geophys. Res.* **1031**, 13041–13054 (1998).
30. Stanton, B. & Sutton, P. Velocity measurements in the East Auckland Current north-east of North Cape, New Zealand. *N. Z. J. Mar. Freshw. Res.* **37**, 195–204 (2003).
31. Bowen, M., Sutton, P. & Roemmich, D. Estimating mean dynamic topography in boundary currents and the use of Argo trajectories. *J. Geophys. Res. Oceans* **119**, 8422–8437 (2014).
32. Sutton, P. The Southland Current: A subantarctic current. *N. Z. J. Mar. Freshw. Res.* **37**, 645–652 (2003).
33. Schott, F., Swallow, J. C. & Fieux, M. The Somali current at the equator: annual cycle of currents and transports in the upper 1000 m and connection to neighbouring latitudes. *Deep Sea Res. Part Oceanogr. Res. Pap.* **37**, 1825–1848 (1990).
34. Leetmaa, A., Quadfasel, D. R. & Wilson, D. Development of the Flow Field during the Onset of the Somali Current, 1979. *J Phys Ocean. U. S.* **12:12**, (1982).
35. Swallow, J. C., Schott, F. & Fieux, M. Structure and transport of the East African Coastal Current. *J. Geophys. Res. Oceans* **96**, 22245–22257 (1991).
36. Swallow, J., Fieux, M. & Schott, F. The boundary currents east and north of Madagascar: 1. Geostrophic currents and transports. *J. Geophys. Res. Oceans* **93**, 4951–4962 (1988).

37. Schott, F., Fieux, M., Kindle, J., Swallow, J. & Zantopp, R. The boundary currents east and north of Madagascar: 2. Direct measurements and model comparisons. *J. Geophys. Res. Oceans* **93**, 4963–4974 (1988).

38. Voldsund, A., Aguiar-González, B., Gammelsrød, T., Krakstad, J. & Ullgren, J. Observations of the East Madagascar Current system: Dynamics and volume transports. *J. Mar. Res.* **75**, 531–555 (2017).

39. Ponsoni, L., Aguiar-González, B., Ridderinkhof, H. & Maas, L. R. M. The East Madagascar Current: Volume Transport and Variability Based on Long-Term Observations. *J. Phys. Oceanogr.* **46**, 1045–1065 (2016).

40. Donohue, K. A. & Toole, J. M. A near-synoptic survey of the Southwest Indian Ocean. *Deep Sea Res. Part II Top. Stud. Oceanogr.* **50**, 1893–1931 (2003).

41. Nauw, J. J., Aken, H. M. van, Webb, A., Lutjeharms, J. R. E. & Ruijter, W. P. M. de. Observations of the southern East Madagascar Current and undercurrent and countercurrent system. *J. Geophys. Res. Oceans* **113**, (2008).

42. de Ruijter, W. P. M. de, Ridderinkhof, H., Lutjeharms, J. R. E., Schouten, M. W. & Veth, C. Observations of the flow in the Mozambique Channel. *Geophys. Res. Lett.* **29**, 140-1-140–3 (2002).

43. Ridderinkhof, H. *et al.* Seasonal and interannual variability in the Mozambique Channel from moored current observations. *J. Geophys. Res. Oceans* **115**, (2010).

44. DiMarco, S. F. *et al.* Volume transport and property distributions of the Mozambique Channel. *Deep Sea Res. Part II Top. Stud. Oceanogr.* **49**, 1481–1511 (2002).

45. Bryden, H. L., Beal, L. M. & Duncan, L. M. Structure and Transport of the Agulhas Current and Its Temporal Variability. *J. Oceanogr.* **61**, 479–492 (2005).

46. Beal, L. M., Elipot, S., Houk, A. & Leber, G. M. Capturing the Transport Variability of a Western Boundary Jet: Results from the Agulhas Current Time-Series Experiment (ACT). *J. Phys. Oceanogr.* **45**, 1302–1324 (2015).

47. Gordon, A. L., Lutjeharms, J. R. E. & Gründlingh, M. L. Stratification and circulation at the Agulhas Retroflection. *Deep Sea Res. Part Oceanogr. Res. Pap.* **34**, 565–599 (1987).

48. Gordon, A. L., Weiss, R. F., Smethie, W. M. & Warner, M. J. Thermocline and Intermediate Water Communication between the South-Atlantic and Indian Oceans. *J. Geophys. Res.-Oceans* **97**, 7223–7240 (1992).

49. de Ruijter, W. P. M. de *et al.* Indian-Atlantic interocean exchange: Dynamics, estimation and impact. *J. Geophys. Res. Oceans* **104**, 20885–20910 (1999).

50. Boebel, O. *et al.* The Cape Cauldron: a regime of turbulent inter-ocean exchange. *Deep Sea Res. Part II Top. Stud. Oceanogr.* **50**, 57–86 (2003).

51. Mercier, H., Arhan, M. & Lutjeharms, J. R. E. Upper-layer circulation in the eastern Equatorial and South Atlantic Ocean in January–March 1995. *Deep Sea Res. Part Oceanogr. Res. Pap.* **50**, 863–887 (2003).

52. Richardson, P. L. Agulhas leakage into the Atlantic estimated with subsurface floats and surface drifters. *Deep Sea Res. Part Oceanogr. Res. Pap.* **54**, 1361–1389 (2007).

53. Garzoli, S. L., Ffield, A., Johns, W. E. & Yao, Q. North Brazil Current retroflection and transports. *J. Geophys. Res. Oceans* **109**, (2004).

54. Johns, W. E. *et al.* Annual Cycle and Variability of the North Brazil Current. *J. Phys. Oceanogr.* **28**, 103–128 (1998).

55. Hummels, R. *et al.* Interannual to decadal changes in the western boundary circulation in the Atlantic at 11°S. *Geophys. Res. Lett.* **42**, 7615–7622 (2015).

56. Stramma, L., Ikeda, Y. & Peterson, R. G. Geostrophic transport in the Brazil current region north of 20°S. *Deep Sea Res. Part Oceanogr. Res. Pap.* **37**, 1875–1886 (1990).

57. Evans, D. L. & Signorini, S. S. Vertical structure of the Brazil Current. *Nature* **315**, 48–50 (1985).

58. Müller, T. J., Ikeda, Y., Zangenberg, N. & Nonato, L. V. Direct measurements of western boundary currents off Brazil between 20°S and 28°S. *J. Geophys. Res. Oceans* **103**, 5429–5437 (1998).

59. Stramma, L. The Brazil current transport south of 23°S. *Deep Sea Res. Part Oceanogr. Res. Pap.* **36**, 639–646 (1989).

60. Garzoli, S. L. Geostrophic velocity and transport variability in the Brazil-Malvinas Confluence. *Deep Sea Res. Part Oceanogr. Res. Pap.* **40**, 1379–1403 (1993).

61. Lentini, C. A. D., Goni, G. J. & Olson, D. B. Investigation of Brazil Current rings in the confluence region. *J. Geophys. Res. Oceans* **111**, (2006).

62. Garzoli, S. L. & Garraffo, Z. Transports, frontal motions and eddies at the Brazil-Malvinas currents confluence. *Deep Sea Res. Part Oceanogr. Res. Pap.* **36**, 681–703 (1989).

63. Gordon, A. L. & Greengrove, C. L. Geostrophic circulation of the Brazil-Falkland confluence. *Deep Sea Res. Part Oceanogr. Res. Pap.* **33**, 573–585 (1986).

64. Artana, C. *et al.* The Malvinas Current at the Confluence With the Brazil Current: Inferences From 25 Years of Mercator Ocean Reanalysis. *J. Geophys. Res. Oceans* **124**, 7178–7200 (2019).

65. Schmid, C. & Majumder, S. Transport variability of the Brazil Current from observations and a data assimilation model. *Ocean Sci.* **14**, 417–436 (2018).

66. Goes, M., Cirano, M., Mata, M. M. & Majumder, S. Long-Term Monitoring of the Brazil Current Transport at 22°S From XBT and Altimetry Data: Seasonal, Interannual, and Extreme Variability. *J. Geophys. Res. Oceans* **124**, 3645–3663 (2019).

67. Halkin, D. & Rossby, T. The Structure and Transport of the Gulf Stream at 73°W. *J. Phys. Oceanogr.* **15**, 1439–1452 (1985).

68. Zhao, J. & Johns, W. Wind-Driven Seasonal Cycle of the Atlantic Meridional Overturning Circulation. *J. Phys. Oceanogr.* **44**, 1541–1562 (2014).

69. Johns, W. E., Shay, T. J., Bane, J. M. & Watts, D. R. Gulf Stream structure, transport, and recirculation near 68°W. *J. Geophys. Res. Oceans* **100**, 817–838 (1995).

70. Richardson, P. Average velocity and transport of the Gulf Stream near 55W. *J. Mar. Res.* **43**, 83–111 (1985).

71. Hogg, N. G. On the transport of the gulf stream between cape hatteras and the grand banks. *Deep Sea Res. Part Oceanogr. Res. Pap.* **39**, 1231–1246 (1992).

72. Beal, L. M. *et al.* Five years of Florida Current structure and transport from the Royal Caribbean Cruise Ship Explorer of the Seas. *J. Geophys. Res. Oceans* **113**, (2008).

73. Sturges, W. & Hong, B. Gulf Stream Transport Variability at Periods of Decades. *J. Phys. Oceanogr.* **31**, 1304–1312 (2001).

74. Hamilton, P., Larsen, J. C., Leaman, K. D., Lee, T. N. & Waddell, E. Transports through the Straits of Florida. *J. Phys. Oceanogr.* **35**, 308–322 (2005).

75. Sheinbaum, J., Candela, J., Badan, A. & Ochoa, J. Flow structure and transport in the Yucatan Channel. *Geophys. Res. Lett.* **29**, 10-1-10–4 (2002).

76. Candela, J., Tanahara, S., Crepon, M., Barnier, B. & Sheinbaum, J. Yucatan Channel flow: Observations versus CLIPPER ATL6 and MERCATOR PAM models. *J. Geophys. Res. Oceans* **108**, (2003).

77. Rousset, C. & Beal, L. M. Observations of the Florida and Yucatan Currents from a Caribbean Cruise Ship. *J. Phys. Oceanogr.* **40**, 1575–1581 (2010).
